# Engineered Transformer Base Editor with Enhanced Editing Efficiency

**DOI:** 10.1002/advs.77657

**Published:** 2026-09-10

**Authors:** Bowen Chen, Letong Liang, Rui Xu, Tianyi Wang, Hongyan Chen, Daru Lu

**Affiliations:** ^1^ State Key Laboratory of Genetics and Development of Complex Phenotypes and MOE Engineering Research Center of Gene Technology, School of Life Sciences, and Taizhou Institute of Health Science Fudan University Shanghai People's Republic of China

**Keywords:** CRISPR‐Cas9, cytidine deaminase, cytosine base editor, genome editing, off‐target, protein engineering, rational design, transformer base editor

## Abstract

Canonical cytosine base editors (CBEs) achieve precise C‐to‐T conversions without inducing DNA double‐strand breaks (DSBs), yet their clinical potential remains hampered by substantial off‐target (OT) mutations. The recently developed transformer base editor (tBE) significantly reduces both genomic and transcriptomic OT mutations by using a cleavable deoxycytidine deaminase inhibitor (dCDI) domain. However, the modest base editing efficiency limits its broader applications. Here, through rational deaminase engineering and fusion of a uracil DNA glycosylase inhibitor (UGI) domain, we developed the engineered tBE (etBE). The etBE exhibited substantially enhanced editing efficiencies compared with the parental tBE (up to 35.11‐fold improvement), while maintaining high editing fidelity and background levels of OT mutations. As a therapeutic proof‐of‐concept, dual adeno‐associated virus (AAV)‐mediated delivery of etBE targeting proprotein convertase subtilisin/kexin type 9 (*PCSK9*), a well‐established therapeutic target for cardiovascular diseases, was evaluated in a humanized mouse model. The treatment achieved efficient in vivo base editing (up to 35.13%), resulting in substantial reductions in plasma PCSK9 protein (24%) and low‐density lipoprotein cholesterol (LDL‐C) levels (33%), while inducing only minimal OT mutations. Collectively, etBE represents a highly efficient and specific base editing platform with enormous potential for both basic research and clinical applications.

## Graphical abstract

A highly efficient transformer base editor system achieves robust genomic editing in a humanized mouse model. This work establishes a versatile and translatable platform, opening new avenues for precision gene therapy.

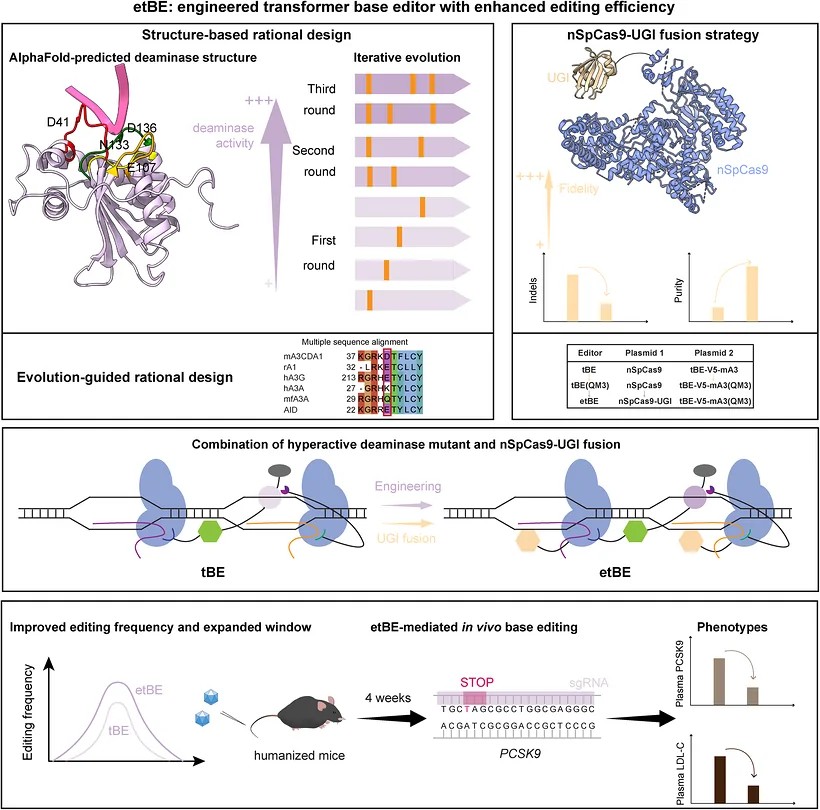

## 1 Introduction

The CRISPR‐Cas systems, such as Type II Cas9 [1] and Type V Cas12 [2], are widely used for gene knockout and homology‐directed repair. However, the DNA double‐strand breaks (DSBs) induced by the nucleases can trigger undesirable cellular events, including p53‐mediated DNA damage responses [3, 4], large fragment deletions [5, 6], and chromothripsis [7]. To overcome these restrictions, cytosine base editors (CBEs) were developed by fusing the apolipoprotein B mRNA‐editing enzyme, catalytic polypeptide‐like (APOBEC) family of cytidine deaminases (CDAs) with *Streptococcus pyogenes* Cas9 D10A nickase (nSpCas9) and uracil DNA glycosylase inhibitor (UGI) domain, enabling programmable C‐to‐T transitions without generating DSBs [8, 9, 10]. Nevertheless, canonical CBEs induce pronounced genomic [11, 12, 13] and transcriptomic [14, 15] off‐target (OT) mutations, which severely limit their therapeutic applications. To address the safety concerns, Wang et al. previously established the transformer base editor (tBE) [16]. They initially found that mouse APOBEC3 (mA3) contains two CDA domains. The N‐terminal domain (mA3CDA1) is catalytically active and capable of cytosine deamination, whereas the C‐terminal domain (mA3CDA2) functions as a deoxycytidine deaminase inhibitor (dCDI) that specifically suppresses mA3CDA1 activity. Due to its inhibitory function, mA3CDA2 is hereafter referred to as mA3dCDI. The tBE system is constructed by exploiting the natural regulatory mechanism.

The tBE system utilizes a dual‐plasmid design (Figure S1A). One plasmid expresses the nSpCas9 protein, which serves as both the DNA‐binding module and R‐loop generator. The second plasmid (tBE‐V5‐mA3) encodes two single‐guide RNAs (sgRNAs) and two fusion proteins. Orthogonal RNA aptamer‐protein recruitment systems—MS2‐MS2 coat protein (MCP) [17] and boxB‐N peptide from bacteriophage P22 (N22p) [18]—enable specific assembly of the editing machinery. Specifically, one sgRNA containing an MS2 hairpin, which is denoted as helper sgRNA (hsgRNA), recruits a cleavable MCP‐UGI‐mA3CDA1‐mA3dCDI fusion protein. In the fusion protein, the UGI domain inhibits endogenous uracil DNA glycosylase (UNG) to prevent excision of the deaminated uracil, the mA3CDA1 domain catalyzes target cytosine deamination, while the mA3dCDI domain represses mA3CDA1 activity. The mA3CDA1 and mA3dCDI domains are connected by a tobacco etch virus (TEV) protease cleavage site. The second sgRNA, incorporated with two boxB hairpins, recruits the N22p‐TEV protease fusion protein and generates the R‐loop for the intended editing. At on‐target sites where both hsgRNA and sgRNA bind, the recruited TEV protease cleaves off the inhibitory mA3dCDI domain, thereby activating mA3CDA1 for targeted editing (Figure S1B). In contrast, at OT loci where either hsgRNA or sgRNA is absent, the mA3dCDI domain suppresses mA3CDA1 activity, rendering tBE inactive (Figure S1C–E). Consequently, tBE achieves highly specific base editing with background levels of OT mutations. However, the relatively modest editing efficiency limits its further applications.

The enzymatic properties of CBEs are largely determined by the CDAs employed. Previous studies have shown that engineering CDAs is an effective strategy to modulate CBE performance [19, 20, 21, 22]. In this study, we rationally designed and screened 142 single‐amino‐acid substitutions in mA3CDA1, identifying 18 beneficial variants that enhanced deaminase activity. Through iterative combinatorial engineering of six top‐performing mutations, we generated a quadruple mutant, designated mA3CDA1(QM3), which substantially improved base editing efficiency across many tested genomic sites. Although subsequent characterization revealed that the increased base editing efficiency was accompanied by a rise in editing byproducts, fusing a single UGI domain to the C‐terminus of nSpCas9 effectively suppressed byproduct formation and restored editing fidelity. We therefore combined nSpCas9‐UGI with mA3CDA1(QM3) to create the engineered tBE (etBE). The etBE displayed markedly higher C‐to‐T editing efficiencies across diverse genomic loci and cell types, while exhibiting minimal OT mutations. Furthermore, dual adeno‐associated virus (AAV)‐mediated delivery of etBE targeting proprotein convertase subtilisin/kexin type 9 (*PCSK9*) achieved efficient in vivo editing in humanized C57BL/6J mice, along with substantial reductions in both plasma PCSK9 protein and low‐density lipoprotein cholesterol (LDL‐C) levels. Collectively, etBE represents a robust and safe base editing platform with considerable potential.

## 2 Results

### 2.1 Rational Design of Beneficial Substitutions in mA3CDA1

**FIGURE 1:**
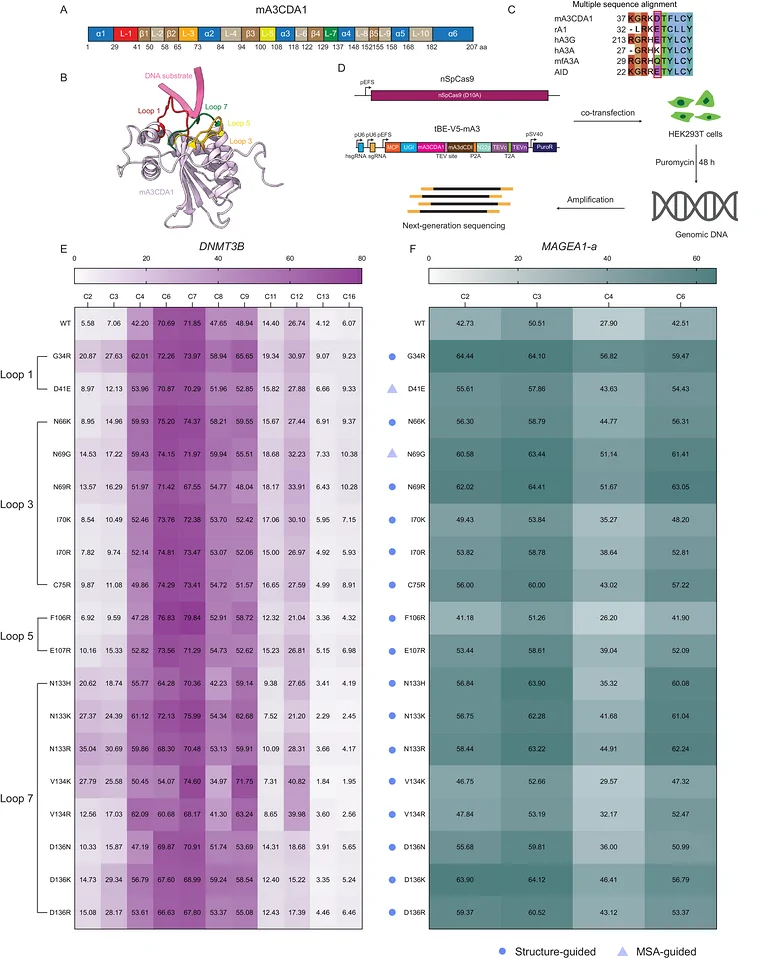
Rational approaches to identify beneficial amino acid substitutions in mA3CDA1. (A) Domain architecture of mA3CDA1. The mA3CDA1 comprises six α‐helices (blue), five β‐sheets (dark brown), and ten loops, shown to scale. Loops 1, 3, 5, and 7 are colored red, orange, yellow, and green, respectively, while the remaining loops are light brown. aa, amino acid. (B) Predicted structure of mA3CDA1 in complex with substrate DNA, generated by AlphaFold 2. The mA3CDA1 domain is shown in pink, substrate DNA in magenta, and loops 1, 3, 5, and 7 in red, orange, yellow, and green, respectively. (C) Multiple sequence alignment of mA3CDA1 with rat APOBEC1 (rA1), human APOBEC3G (hA3G), human APOBEC3A (hA3A), macaca fascicularis APOBEC3A (mfA3A), and human activation‐induced cytidine deaminase (AID). A representative region is shown, with the candidate residue for mutagenesis highlighted in a red box. The full sequence alignment is provided in Figure S2D. (D) Workflow for evaluating base editing activities of mA3CDA1(WT) and variants. (E and F) Base editing efficiencies of mA3CDA1(WT) and beneficial single‐substitution mutants at the *DNMT3B* (E) and *MAGEA1‐a* (F) endogenous loci in HEK293T cells. Data represent mean values from two independent biological replicates. Amino acid substitutions identified through structure‐guided design are highlighted by blue solid circles, while those identified through multiple sequence alignment are marked by light blue triangles.

The mA3CDA1 domain, comprising 207 amino acids organized into six α‐helices, five β‐sheets, and ten loops (Figure 1A), is the primary determinant of tBE editing performance. The DNA substrate‐binding groove of CDAs plays a critical role in substrate recognition and catalysis. Consequently, mutations within this region can strongly modulate deaminase activity [23, 24]. We therefore performed rational engineering of mA3CDA1, focusing primarily on the DNA‐binding groove, to enhance tBE activity. Because a crystal structure for mA3CDA1 was unavailable, we first generated a high‐confidence structural model using AlphaFold 2 [25] (Figure 1B). Comparing this model with the crystal structures of human APOBEC3A (hA3A) [26] and APOBEC3G (hA3G) [27] confirmed a conserved overall fold (Figure S2A,B) [28]. The DNA substrate‐binding groove of mA3CDA1 is predominantly formed by loops 1, 3, 5, and 7 (Figure S2C), which served as our primary target regions. Since CDA‐mediated deamination is a multi‐turnover process, increasing the binding affinity of CDAs for substrate DNA could prolong deaminase residence time, improve processivity, and thereby enhance base editing efficiency. Thus, we targeted residues in close proximity to the DNA substrate based on our predicted structural model and introduced positively charged amino acids at these positions (e.g., G34R and D136K) to strengthen electrostatic interactions with the DNA backbone.

Although the predicted structural model provides direct spatial information, it may not capture all relevant conformational states. Thus, we employed a second, complementary method. We identified additional candidate substitutions through a multiple sequence alignment (MSA) of mA3CDA1 with other active CDA orthologs (Figure S2D). Here, we introduced evolutionarily conserved substitutions (e.g., D41E and I74L) (Figure 1C) to improve thermostability. Frequently occurring consensus residues at a given position within a protein family generally confer greater structural stability than rare variants, a strategy that has previously proven effective for increasing IscB activity [29]. Guided by the structural and MSA analyses, we designed a total of 104 single‐amino‐acid substitution mutants within the catalytic pocket (Figure S3A–D). Concurrently, amino acid substitutions outside the catalytic pocket can influence deaminase activity by modulating substrate access channels and overall protein conformational dynamics, so we selected and screened an additional 38 mutants outside the pocket using MSA‐based analysis (Figure S3E).

We selected an endogenous NGG protospacer adjacent motif (PAM) locus (*DNMT3B*) harboring 11 cytosines distributed across the protospacer to comprehensively evaluate editing performance across the PAM‐distal (C1–C3), canonical (C4–C8), and PAM‐proximal (C9–C20) regions (PAM at positions 21–23). The tBE‐V5‐mA3 constructs carrying mA3CDA1 mutants or the wild‐type (WT) control were co‐transfected with the nSpCas9‐expressing plasmid into human embryonic kidney 293T (HEK293T) cells. Successfully transfected cells were enriched by puromycin selection (4 µg/mL) 24 h post‐transfection, as the tBE‐V5‐mA3 plasmid contains an SV40 promoter‐driven puromycin resistance gene (PuroR) expression cassette. Selected cells were harvested 48 h post‐selection and subjected to next‐generation sequencing (NGS) (Figure 1D). Sequencing data were analyzed using CRISPResso2 [30]. C‐to‐T editing efficiency is defined as the percentage of sequencing reads in which the target cytosine is converted to thymine at the intended position. It is calculated as T / (A + T + C + G), where A, T, C, and G represent the respective nucleotide read counts at the position.

NGS analysis of the 142 variants identified 18 beneficial substitutions at 11 amino acid positions (Figure 1E). All these substitutions were located within the substrate‐binding groove (two in loop 1, six in loop 3, two in loop 5, and eight in loop 7), highlighting its critical role in deaminase activity. Of the 18 beneficial substitutions, 16 were identified through structure‐guided screening (G34R, N66K, N69R, I70K/R, C75R, F106R, E107R, N133H/K/R, V134K/R, and D136H/K/R), while 2 were selected via MSA (D41E and N69G). We observed limited improvement at C6 and C7 positions at the *DNMT3B* locus, likely due to near‐saturation of editing efficiency (∼70%) caused by transfection limitations in HEK293T cells. Improvements at PAM‐proximal positions (particularly C13 and C16) were also modest, possibly owing to steric hindrance [31, 32] and R‐loop stability [33, 34]. Beyond these positions, the 18 beneficial substitutions could be classified into three categories based on their editing patterns: (1) substitutions that primarily enhanced editing in the C2–C4 region (D41E, I70K/R, N133H, and D136N); (2) substitutions that mainly improved editing in the C8–C11 region (C75R, F106R, V134K/R); and (3) substitutions that enhanced editing in both regions (G34R, N66K, N69G/R, E107R, N133K/R, and D136K/R). Among them, N133R showed the largest improvement, increasing editing at C2 by 6.28‐fold (35.04% vs. 5.58%) (Figure 1E). These variants were further validated at an additional endogenous NGG PAM locus (*MAGEA1*‐*a*) in HEK293T cells under the same conditions (Figure 1F). The same substitutions consistently outperformed the mA3CDA1(WT) control, with G34R showing the strongest enhancement at C4 (56.82% vs. 27.90%). Notably, multiple variants exhibited substantial improvements at the efficiently modified C2, C3, and C6 positions at the *MAGEA1*‐*a* locus, further confirming the robustness of these beneficial substitutions. Besides, we observed notable sequence‐context‐dependent effects at the tested genomic loci. For example, editing efficiency at C12 was substantially higher than at C11 and C13 at the *DNMT3B* locus, while editing at C4 was notably lower than at C3 and C6 at the *MAGEA1*‐*a* locus. Collectively, guided by the predicted structure model and MSA, we identified 18 beneficial substitutions that significantly enhance deaminase activity.

### 2.2 Combinatorial Engineering of Beneficial Substitutions Generated a Quadruple Mutant with Improved Base Editing Efficiency

Previous studies have shown that combining multiple beneficial substitutions can produce additive or synergistic effects, further enhancing deaminase activities [35, 36]. We therefore applied this strategy to the six top‐performing substitutions (G34R, D41E, N69G, E107R, N133R, and D136K) identified in the initial screen. Given that editing efficiencies at the *DNMT3B* site may have approached saturation, we selected one non‐NGG PAM genomic locus (*CUL3*), where our recently developed tBE‐SpRY [37] (replacing the SpCas9 with the PAM‐flexible SpRY [38] variant) exhibited modest base editing frequency.

**FIGURE 2:**
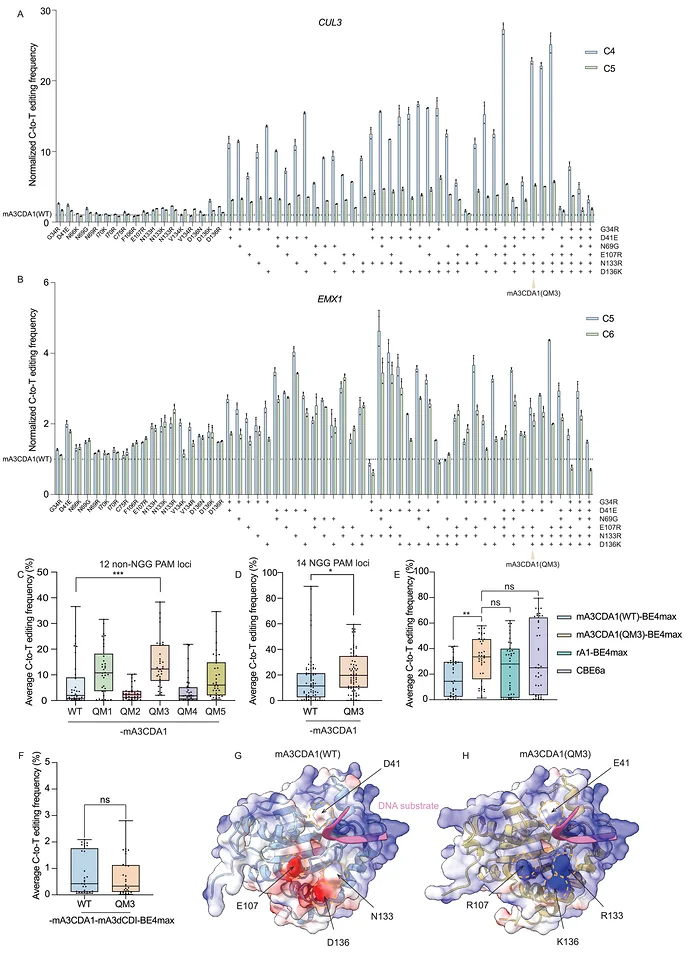
Comprehensive evaluation of combinatorial mA3CDA1 variants. (A and B) Fold changes in base editing efficiencies of mA3CDA1 variants harboring single, double, triple, quadruple, quintuple, and sextuple amino acid substitutions at the *CUL3* (A) and *EMX1* (B) loci, normalized to mA3CDA1(WT). The horizontal dashed line indicates mA3CDA1(WT) performance (fold change = 1). The mA3CDA1(QM3) mutant is highlighted with an orange triangle. Data represent the mean ± SD from two independent experiments. (C) Summary of C‐to‐T editing efficiencies of mA3CDA1(QM1), mA3CDA1(QM2), mA3CDA1(QM3), mA3CDA1(QM4), mA3CDA1(QM5), and mA3CDA1(WT) within tBE architecture at 12 non‐NGG PAM endogenous loci in HEK293T cells. Statistical significance was determined by one‐way ANOVA with Tukey's multiple comparisons (^***^ *p* < 0.001). (D) Summary of C‐to‐T editing efficiencies of mA3CDA1(WT) and mA3CDA1(QM3) within tBE architecture at 14 NGG PAM endogenous loci in HEK293T cells. Statistical significance was measured by using an unpaired two‐tailed Student's *t*‐test (^*^ *p* < 0.05). (E) Summary of base editing frequencies of mA3CDA1(WT)‐BE4max, mA3CDA1(QM3)‐BE4max, rA1‐BE4max, and CBE6a across all 12 tested sites in HEK293T cells. Statistical significance was determined by one‐way ANOVA with Tukey's multiple comparisons (ns, not significant; ^**^ *p* < 0.01). The full dataset is shown in Figure S7B. (F) Summarized average C‐to‐T editing efficiencies of mA3CDA1(WT)‐mA3dCDI‐BE4max and mA3CDA1(QM3)‐mA3dCDI‐BE4max at eight genomic loci in HEK293T cells. Statistical significance was determined by an unpaired two‐tailed Student's *t*‐test (ns, not significant). The full dataset is displayed in Figure S8B. (G and H) Electrostatic surface potentials of mA3CDA1(WT) (G) and mA3CDA1(QM3) (H). The D41E, E107R, N133R, and D136K mutations are indicated by dashed circles and arrows. Positively charged, negatively charged, and neutral regions are depicted in blue, red, and white, respectively.

We first constructed all 15 possible double mutants. To enable direct comparison, base editing efficiencies of single and double mutants were measured in parallel experiments and normalized to the mA3CDA1(WT) control to calculate fold changes (Figure S4A). At the *CUL3* locus, all double mutants outperformed their corresponding single mutant counterparts. At position C4, fold improvements ranged from 0.85‐ to 3.00‐fold for single mutants and 5.49‐ to 15.44‐fold for double mutants. At C5, fold changes ranged from 0.80‐ to 1.89‐fold and 2.00‐ to 3.76‐fold, respectively (Figure 2A). Among the 15 double mutants, six showed more than tenfold improvement at C4, with the D41E/D136K combination yielding the highest enhancement. Similar trends were observed at another independent non‐NGG PAM locus (*EMX1*). At this site, fold changes ranged from 1.11‐ to 2.03‐fold for single mutants and from 1.56‐ to 4.03‐fold for double mutants at position C5 (Figure 2B and Figure S4B). The magnitude of improvement was more modest at the *EMX1* locus than at the *CUL3* locus, likely due to sequence‐context effects. Together, these results demonstrate that the beneficial substitutions exert a consistent, sequence‐independent additive effect on base editing efficiency.

Encouraged by the finding, we next constructed triple mutants based on the performance of the top double mutants. Five promising double mutants (D41E/N133R, D41E/D136K, N133R/D136K, G34R/N133R, and G34R/D136K) were selected, from which we generated all possible combinations, yielding 14 triple mutants. Deaminase activity improved progressively with increasing numbers of substitutions. At the *CUL3* locus, fold changes for triple mutants ranged from 1.59‐ to 16.64‐fold at C4 and 1.45‐ to 16.47‐fold at C5 (Figure 2A and Figure S4A). A similar pattern was observed at the *EMX1* locus, with fold changes ranging from 0.61‐ to 4.62‐fold at C5 and from 0.61‐ to 3.44‐fold at C6 (Figure 2B and Figure S4B). Notably, some sequence‐dependent inconsistencies were detected. For instance, the G34R/D41E/N133R variant showed strong activity at *CUL3* C4 (12.47‐fold) but compromised performance at *EMX1* C5 (0.88‐fold), highlighting the influence of target sequence context on deaminase activity.

Based on the combined performance at both *CUL3* and *EMX1* loci, three promising triple mutants (D41E/N69G/N133R, D41E/E107R/N133R, and G34R/N133R/D136K) were selected for quadruple mutant construction. We generated six quadruple mutants and observed further steady improvement. Four of these variants achieved over 20‐fold enhancement at the *CUL3* C4 position, with the D41E/N69G/E107R/N133R combination reaching up to 27.59‐fold (Figure 2A and Figure S4A). Consistent improvements were also observed at the *EMX1* locus, where G34R/E107R/N133R/D136K showed the highest fold change at C5 position (4.37‐fold) (Figure 2B and Figure S4B). However, further addition of mutations to generate quintuple and sextuple mutants resulted in a sharp decline in activity. Fold changes dropped to 1.95–7.83 for quintuple mutants and 3.15 for the sextuple mutant at *CUL3* C4 (Figure 2A and Figure S4A). A similar reduction was also observed at *EMX1* (Figure 2B and Figure S4B). We hypothesize that this decrease arises from steric hindrance or negative epistatic effects caused by excessive mutations. Significantly, strong sequence‐context dependence persisted. The best‐performing quadruple mutant at *CUL3* (D41E/N69G/E107R/N133R) exhibited only modest improvement at *EMX1* (27.59‐fold at *CUL3* C4 vs. 1.58‐fold at *EMX1* C5).

To identify one broadly effective variant, we selected five quadruple mutants for further assessment across 12 additional non‐NGG PAM sites in HEK293T cells (Figure S5): mA3CDA1(QM1) (D41E/N69G/E107R/N133R), mA3CDA1(QM2) (D41E/N69G/N133R/D136K), mA3CDA1(QM3) (D41E/E107R/N133R/D136K), mA3CDA1(QM4) (G34R/D41E/N133R/D136K), and mA3CDA1(QM5) (G34R/E107R/N133R/D136K). NGS analysis revealed that mA3CDA1(QM1) and mA3CDA1(QM3) consistently outperformed the other variants, achieving average editing efficiencies of 11.87% and 14.27%, respectively, compared with 6.40% for mA3CDA1(WT) (Figure 2C). The mA3CDA1(QM3) variant exhibited robust and stable performance across the majority of tested genomic sites and was therefore selected for subsequent experiments. We further compared mA3CDA1(QM3) with mA3CDA1(WT) at 14 endogenous NGG PAM sites in HEK293T cells. As shown in Figure 2D and Figure S6, mA3CDA1(QM3) achieved higher average editing efficiencies (mean: 22.56% vs. 15.76%). Taken together, iterative combinatorial engineering yielded the mA3CDA1(QM3) quadruple mutant, which outperformed mA3CDA1(WT) at most tested loci.

### 2.3 Comprehensive Evaluation of mA3CDA1(QM3) Deaminase Variant

Previous comparisons between mA3CDA1(WT) and mA3CDA1(QM3) were performed within the tBE architecture, which involves multiple components and orthogonal aptamer‐protein recruitment systems. To directly evaluate the intrinsic deaminase activity of mA3CDA1(WT) and mA3CDA1(QM3), we replaced the rat APOBEC1 (rA1) in the canonical rA1‐BE4max [10] architecture (rA1‐nSpCas9‐2 × UGI) with either mA3CDA1(WT) or mA3CDA1(QM3), generating mA3CDA1(WT)‐BE4max and mA3CDA1(QM3)‐BE4max, respectively (Figure S7A). We then compared the two constructs with the canonical rA1‐BE4max and the state‐of‐the‐art TadA‐derived CBE [39] (CBE6a) at 12 endogenous genomic loci (six with NGG PAMs and six with non‐NGG PAMs) in HEK293T cells (Figure S7B). Deep sequencing analysis showed that mA3CDA1(QM3)‐BE4max outperformed mA3CDA1(WT)‐BE4max at 10 of 12 sites, with average editing efficiencies of 32.16% versus 17.10%, respectively (Figure 2E). Remarkably, mA3CDA1(QM3)‐BE4max achieved editing efficiencies comparable to those of rA1‐BE4max (24.59%) and CBE6a (32.17%). Western blot analysis confirmed comparable protein expression levels between mA3CDA1(WT)‐BE4max and mA3CDA1(QM3)‐BE4max (Figure S7C,D). Overall, these data confirm the superior deaminase activity of mA3CDA1(QM3) relative to mA3CDA1(WT). Moreover, mA3CDA1(QM3)‐BE4max provides a robust CBE with performance comparable to rA1‐BE4max and CBE6a, further enriching the CBE toolbox.

The inhibitory effect of mA3dCDI on mA3CDA1(WT) is critical for suppressing tBE activity at OT loci. To determine whether the amino acid substitutions in mA3CDA1(QM3) compromise the natural inhibition, we evaluated the inhibitory capacity of mA3dCDI on the engineered deaminase. We fused the mA3dCDI domain to the C‐terminus of the deaminase in both the mA3CDA1(WT)‐BE4max and mA3CDA1(QM3)‐BE4max backbones to mimic the natural domain architecture of mA3 [16]. Therefore, mA3CDA1(WT)‐mA3dCDI‐BE4max and mA3CDA1(QM3)‐mA3dCDI‐BE4max constructs were yielded, respectively (Figure S8A). We parallelly compared the two groups at eight endogenous NGG PAM loci in HEK293T cells (Figure S8B). NGS analysis revealed that both groups exhibited similarly low C‐to‐T editing efficiencies (mean: 0.79% vs. 0.67%), with no significant difference observed (Figure 2F). These results demonstrate that mA3dCDI retains its potent inhibitory effect on mA3CDA1(QM3), keeping the deaminase inactive when being linked. This property is essential for preserving the high specificity of the tBE system.

Prior studies have shown that rA1‐BE4max induces substantial RNA OT mutations [14, 15], whereas mA3CDA1(WT)‐BE4max, mA3dCDI‐BE4max, or mA3CDA1(WT)‐mA3dCDI‐BE4max generate only background levels of RNA variants [16]. The difference is likely attributable to the RNA editing activity of rA1 [40], which is minimal in mA3CDA1 and mA3dCDI [41]. To determine whether the mutations in mA3CDA1(QM3) alter RNA editing specificity, we compared mA3CDA1(WT)‐ and mA3CDA1(QM3)‐induced RNA OT mutations using mA3CDA1(WT)‐BE4max, mA3CDA1(QM3)‐BE4max, mA3CDA1(WT)‐mA3dCDI‐BE4max, and mA3CDA1(QM3)‐mA3dCDI‐BE4max architectures (Figure S9A). HEK293T cells were transfected with the respective single‐plasmid constructs, selected with 4 µg/mL puromycin for 48 h, and harvested for RNA sequencing (RNA‐seq) analysis, as previously described [16, 37, 42]. RNA‐seq analysis using the RNA‐editing analysis pipeline to decode all twelve types of RNA‐editing events (RADAR) [16, 42, 43, 44, 45] revealed that A‐to‐G, G‐to‐A, C‐to‐U, and U‐to‐C edits were the predominant RNA variants, consistent with adenosine deaminase acting on RNA (ADAR) and APOBEC/AID biological activities. No significant differences in the total number of RNA editing events were observed among the four groups in HEK293T cells (1458, 1396, 1347, and 1366, respectively) (Figure S9B). Similar results were obtained when analyzing *Alu*, non‐*Alu* repetitive, and non‐repetitive regions. Focusing specifically on C‐to‐U editing events, the variant counts were also comparable (269, 263, 261, and 262, respectively) (Figure S9C). These data demonstrate that the introduced amino acid substitutions do not compromise the high RNA editing specificity of the deaminase.

To explore possible molecular mechanisms underlying the improved deaminase activity of mA3CDA1(QM3), we analyzed the electrostatic surface potentials and local geometry of the substrate‐binding pocket using AlphaFold‐predicted structures. Compared to mA3CDA1(WT), which displays a predominantly neutral to negatively charged electrostatic surface surrounding the substrate‐binding pocket (Figure 2G), the introduction of key mutations in mA3CDA1(QM3), particularly E107R, N133R, and D136K, appears to remodel the local microenvironment, generating a more positively charged surface (Figure 2H). The most notable change was observed at position 133, where the neutral asparagine in mA3CDA1(WT) is located approximately 5.78 Å from the C4 of the substrate DNA, whereas the substituted arginine positions its positively charged guanidinium group much closer to the DNA phosphate backbone (reducing the distance to ∼2.8 Å) (Figure S10A). The E107R and D136K mutations similarly seem to alleviate local electrostatic repulsion at the periphery of the binding pocket, although the distance reductions are less pronounced (decreasing from 9.24 to 8.56 Å, and 12.44 to 8.94 Å, respectively) (Figures S10B,C). In contrast, the D41E substitution induced no obvious static geometric changes near the substrate (Figure S10D), suggesting that it may contribute through more subtle dynamic effects or evolutionary optimization of protein stability, consistent with its identification via MSA. Collectively, these structural analyses raise the possibility that the enhanced deaminase activity of mA3CDA1(QM3) might result from strengthened electrostatic interactions with the substrate DNA.

Taken together, these results indicate that mA3CDA1(QM3) exhibits significantly higher average base editing efficiency than mA3CDA1(WT), while retaining potent inhibition by mA3dCDI and high RNA editing specificity. The improved activity may be attributable to enhanced electrostatic interactions with the substrate DNA. However, this hypothesis warrants further biochemical validation.

### 2.4 Fusion of a Single UGI Domain to the C‐terminus of nSpCas9 Restored Editing Fidelity

The mA3CDA1 deaminase and its variants are expressed from the tBE‐V5‐mA3 construct. Thus, the tBE‐V5‐mA3 plasmid carrying mA3CDA1(WT) or mA3CDA1(QM3) is hereafter referred to as tBE‐V5‐mA3(WT) or tBE‐V5‐mA3(QM3), respectively. For brevity, the combination of nSpCas9 and tBE‐V5‐mA3(QM3) is designated tBE(QM3), while the original combination of nSpCas9 + tBE‐V5‐mA3(WT) is denoted as tBE hereafter. We next comprehensively characterized the editing performance of tBE(QM3). In addition to C‐to‐T editing efficiency, insertion and deletion (indel) frequency and product purity are two key performance metrics for CBEs. Indels refer to unintended insertion or deletion mutations that result in changes in DNA length at the target locus. The indel frequency is calculated as the percentage of sequencing reads containing insertions or deletions relative to the total number of sequencing reads. Product purity is defined as the percentage of edited sequencing reads (i.e., reads in which the target cytosine is converted to any other nucleotide) in which the target cytosine is precisely converted to thymine. It is calculated as T / (A + T + G), where T, A, and G represent the read counts with the respective nucleotides at the target position.

Although tBE(QM3) achieved substantially higher C‐to‐T editing efficiencies than tBE at most of the tested genomic loci, it also displayed elevated indel frequencies (7.07% vs. 2.99%) and reduced product purity (84.36% vs. 90.87%) (Figure S11A,B). We hypothesized that the hyperactive mA3CDA1(QM3) deaminase variant induces repeated deamination events, leading to persistent uracil lesions that are recognized and excised by endogenous UNG [8, 9]. This process generates apurinic/apyrimidinic (AP) sites, which in turn promote indel formation and reduce C‐to‐T editing purity. Previous studies have shown that fusion of UGI domains to CBEs effectively inhibits endogenous UNG activity, thereby improving editing fidelity [8, 9]. Inspired by these findings, we adopted a similar strategy here.

**FIGURE 3:**
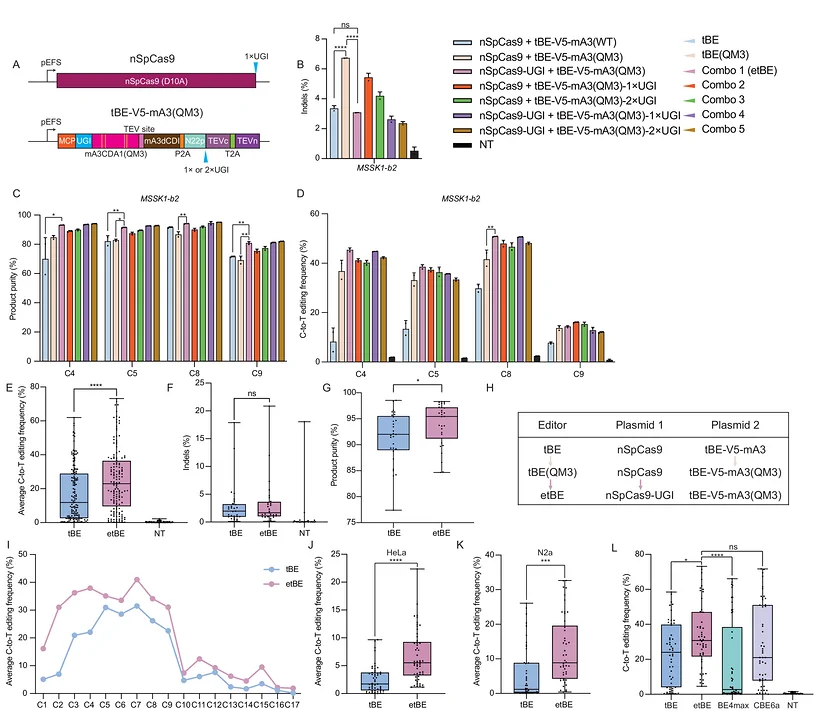
Evaluation of etBE editing performance across genomic loci and cell lines. (A) Schematic architecture of UGI fusion proteins. The inserted UGI domain is highlighted as a blue triangle. (B–D) Indel frequencies (B), product purities (C), and base editing efficiencies (D) of UGI‐fused variants compared with tBE, tBE(QM3), and NT groups at the *MSSK1‐b2* locus. The components of Combo 1–5 groups are indicated. Data represent mean ± SD from two independent biological replicates. Statistical significance was determined by one‐way ANOVA with Tukey's multiple comparisons (ns, not significant; ^*^ *p* < 0.05; ^**^ *p* < 0.01; ^****^ *p* < 0.0001). (E–G) Summary of base editing efficiencies (E), indel frequencies (F), and product purities (G) for tBE, etBE, and NT control across 29 NGG PAM endogenous loci in HEK293T cells. Statistical significance was determined by one‐way ANOVA with Tukey's multiple comparisons in (E) and (F) and by unpaired two‐tailed Student's *t*‐test in (G) (ns, not significant; ^*^ *p* < 0.05; ^****^ *p* < 0.0001). The full dataset is provided in Figure S12. (H) Summary of the components of tBE, tBE(QM3), and etBE. tBE consists of nSpCas9 and tBE‐V5‐mA3; tBE(QM3) consists of nSpCas9 and tBE‐V5‐mA3(QM3); and etBE consists of nSpCas9‐UGI and tBE‐V5‐mA3(QM3). (I) Average C‐to‐T editing efficiencies at each protospacer position across the 29 NGG PAM loci in HEK293T cells. Data represent mean values. (J) Summary of base editing efficiencies for tBE and etBE at 17 NGG PAM loci in HeLa cells. Statistical significance was determined by unpaired two‐tailed Student's *t*‐test (^****^ *p* < 0.0001). The full dataset is shown in Figure S18. (K) Summary of base editing efficiencies for tBE and etBE at 11 endogenous loci in N2a cells. Statistical significance was determined by an unpaired two‐tailed Student's *t*‐test (^***^ *p* < 0.001). The full dataset is displayed in Figure S19. (L) Summarized base editing efficiencies for tBE, etBE, BE4max, and CBE6a at 11 NGG PAM loci in HEK293T cells. Statistical significance was determined by one‐way ANOVA Tukey's multiple comparisons (ns, not significant; ^*^ *p* < 0.05; ^****^ *p* < 0.0001). The full dataset is shown in Figure S20.

We tested multiple UGI fusion configurations, including fusion of one UGI domain to the C‐terminus of nSpCas9 (nSpCas9‐UGI), incorporation of one or two UGI domains into the tBE‐V5‐mA3(QM3) architecture (denoted as tBE‐V5‐mA3[QM3]‐1 × UGI or tBE‐V5‐mA3[QM3]‐2 × UGI), and various combinations thereof (Figure 3A). We did not fuse two copies of UGI to nSpCas9 because the SpCas9 coding sequence (CDS) is already ∼4.1 kilobase (kb). Adding one UGI domain (∼0.2 kb) plus the necessary promoter and poly(A) elements approaches the packaging limit of a single AAV vector (∼4.7 kb). In contrast, the compact tBE‐V5‐mA3(QM3) architecture provided sufficient capacity for two UGI domains. The tested five configurations were designated Combo 1–5 for brevity. Combo 1 is nSpCas9‐UGI + tBE‐V5‐mA3(QM3), Combo 2 is nSpCas9 + tBE‐V5‐mA3(QM3)‐1 × UGI, Combo 3 is nSpCas9 + tBE‐V5‐mA3(QM3)‐2 × UGI, Combo 4 is nSpCas9‐UGI + tBE‐V5‐mA3(QM3)‐1 × UGI, and Combo 5 is nSpCas9‐UGI + tBE‐V5‐mA3(QM3)‐2 × UGI. In addition, tBE, tBE(QM3), and non‐transfected (NT) controls were evaluated alongside the five combinations.

The *MSSK1*‐*b2* locus was selected for comparisons because tBE(QM3) exhibited markedly higher indel frequency at this site compared with tBE (6.72% vs. 3.39%). NGS analysis revealed that the five configurations improved editing fidelity to varying degrees. Combo 1, 4, and 5 reduced indel frequency to levels comparable to tBE (3.07%, 2.61%, and 2.34%, respectively), while Combo 2 and 3 showed relatively moderate reductions (5.42% and 4.18%, respectively) (Figure 3B). The UGI fusion strategy also effectively improved product purity. For instance, at the C5 position, Combo 1, 4, and 5 achieved substantially higher purity (91.33%, 92.52%, and 92.57%, respectively) compared with both tBE and tBE(QM3) (81.89% and 82.65%, respectively) (Figure 3C). Similar improvements were observed across other cytosine positions. Notably, the UGI fusion strategy further enhanced editing efficiency at certain positions. At the C8 position, where tBE(QM3) already outperformed tBE (41.49% vs. 29.66%), Combo 1–5 increased C‐to‐T editing efficiencies to 50.77%, 47.82%, 46.55%, 50.57%, and 48.02%, respectively (Figure 3D). We attribute this enhancement to UGI‐mediated inhibition of UNG, which prevents excision of deaminated uracils and thereby favors their conversion to thymine. However, this effect is sequence‐dependent. For instance, at the C9 position, editing efficiencies of Combo 1–5 (11.95%–15.24%) were comparable to those of tBE(QM3) (13.61%).

To further evaluate the robustness of the UGI fusion strategy, we tested an additional, more challenging genomic locus (*DNMT3B*), where tBE(QM3) induced substantially higher levels of editing byproducts. NGS analysis showed that Combo 1, 3, and 5 markedly improved editing fidelity relative to tBE(QM3). These combinations significantly reduced indel frequencies (10.09%, 9.20%, and 7.23%, respectively) to levels comparable to the parental tBE (5.10%) (Figure S11C). They also restored product purity to levels similar to tBE (83.62%–87.81% vs. 86.43% at C9), which was substantially higher than that of tBE(QM3) (60.19%) (Figure S11D). Moreover, at the same position, Combo 1, 3, and 5 substantially increased editing efficiency (33.03%–37.56%), outperforming both tBE (29.10%) and tBE(QM3) (20.71%) (Figure S11E). Although improvements at other cytosine positions were moderate, an overall consistent trend was observed, confirming the broad effectiveness of the UGI fusion strategy.

Among the five configurations, fusing a single UGI domain to the C‐terminus of SpCas9 (Combo 1) showed the best overall performance, while adding extra UGI domains into the tBE‐V5‐mA3(QM3) architecture (Combo 4 and 5) provided only marginal additional benefit. We therefore selected Combo 1 for broader assessment across 29 endogenous NGG PAM sites in HEK293T cells (Figure S12). NGS analysis revealed that Combo 1 outperformed tBE across most tested loci. The average C‐to‐T editing efficiency increased from 17.05% (tBE) to 25.16% with Combo 1 (Figure 3E), while indel frequencies remained comparable (2.93% vs. 3.25%) (Figure 3F). Product purity was also superior with Combo 1, reaching an average of 93.94% compared with 91.42% for tBE (Figure 3G). Given its enhanced overall editing performance, we designated Combo 1 as the engineered tBE (etBE) hereafter (Figure 3H).

To determine whether the UGI fusion strategy could also benefit the parental tBE system, we constructed five analogous UGI‐containing configurations (Combo 6–10) as follows: Combo 6 is nSpCas9‐UGI + tBE‐V5‐mA3(WT), Combo 7 is nSpCas9 + tBE‐V5‐mA3(WT)‐1 × UGI, Combo 8 is nSpCas9 + tBE‐V5‐mA3(WT)‐2 × UGI, Combo 9 is nSpCas9‐UGI + tBE‐V5‐mA3(WT)‐1 × UGI, and Combo 10 is nSpCas9‐UGI + tBE‐V5‐mA3(WT)‐2 × UGI. Five combinations were evaluated in parallel with the parental tBE at the *DNMT3B* locus in HEK293T cells. NGS analysis showed that the editing efficiencies of Combo 6–10 were comparable to that of the parental tBE (e.g., 41.11%–55.89% vs. 52.19% at C7) (Figure S13A). Importantly, indel frequencies were reduced to varying degrees (2.19%–5.03% vs. 6.79%) (Figure S13B), and product purities were improved at certain positions (e.g., 88.18%–92.22% vs. 82.52% at C7) (Figure S13C). Similar results were obtained at an independent locus (*MSSK1‐b2*), where Combo 6–10 exhibited comparable or slightly lower editing efficiencies, reduced indel frequencies (1.07%–1.85% vs. 2.54%), and comparable product purities relative to the parental tBE (Figure S13D–F).

Since fusing a single UGI domain to the C‐terminus of nSpCas9 is broadly applicable for tBE‐V5‐mA3(QM3), we further compared Combo 6 with the parental tBE at an additional eight NGG PAM genomic sites. NGS analysis showed that both groups obtained comparable editing efficiencies (Figure S14A). When normalized to the parental tBE tested in parallel, etBE exhibited substantially higher editing efficiency than Combo 6 at the same eight loci (Figure S14B). Together, these results indicate that the UGI fusion strategy does not broadly improve editing efficiency for the parental tBE. The elevated editing frequencies observed in both tBE(QM3) and etBE are primarily attributable to the engineered mA3CDA1(QM3) deaminase variant, while the UGI domain mainly helps preserve the deaminated uracil and promotes its conversion to thymine.

### 2.5 Comprehensive Characterization of etBE Editing Performance

To comprehensively characterize etBE editing performance, we next compared tBE(QM3) and etBE in parallel experiments across 14 endogenous NGG PAM loci in HEK293T cells, with all metrics normalized to tBE. Both configurations showed similar improvements in editing efficiency, with 4.42‐fold enhancement for tBE(QM3) and 3.46‐fold for etBE (Figure S15A). However, etBE exhibited markedly better fidelity, with a normalized indel rate of only 1.09‐fold relative to tBE, compared with 3.13‐fold for tBE(QM3) (Figure S15B). etBE also displayed higher product purity, with 1.03‐fold versus 0.93‐fold for tBE(QM3) (Figure S15C). Together, etBE achieved improved editing fidelity while maintaining comparable base editing efficiency to tBE(QM3).

Analysis of average editing frequencies across individual protospacer positions revealed that etBE possesses a broadened editing window. Adopting a bystander‐to‐target editing ratio threshold of 20% [39, 46, 47], we found that the editing window expanded from 9 nucleotide (nt) for tBE (C2–C9 and C12) to 11 nt for etBE (C1–C9 and C11–C12) (Figure 3I). The broader editing window substantially expands the targeting range for the tBE system. To comprehensively assess the editing profile of etBE, we partitioned the protospacer into PAM‑distal region (C1–C3), canonical window (C4–C8), and PAM‑proximal region (C9–C20). etBE outperformed tBE across all regions, with the most pronounced gains in the PAM‑distal region, where average efficiencies increased from 11.26% for tBE to 28.31% for etBE (Figure S15D). In the canonical window, etBE achieved 36.16% compared to 27.81% for tBE (Figure S15E), and in the PAM‑proximal region, efficiencies rose from 7.30% to 11.53% (Figure S15F). We also evaluated the sequence‑context specificity of etBE across different NC dinucleotide motifs. etBE achieved significantly higher C‑to‑T efficiencies across multiple sequence contexts. At TC motifs (*n * =  38), average efficiencies increased from 18.63% for tBE to 28.11% for etBE (Figure S15G). Similar gains were observed at CC (*n*  =  59, from 20.17% to 27.39%) and GC motifs (*n*  =  28, from 14.52% to 24.44%) (Figure S15H,I). A comparable trend was evident at AC motifs (*n*  =  19, from 7.92% to 13.39%), although this difference did not reach statistical significance, likely due to the smaller cohort size (Figure S15J). Overall, etBE exhibited significantly enhanced C‐to‐T editing efficiencies across the entire protospacer and various NC dinucleotide motifs.

Previous studies have shown that the tBE system can induce C‐to‐T editing at both sgRNA and hsgRNA loci [16, 37]. We therefore analyzed base editing efficiencies at hsgRNA loci for both tBE and etBE across 29 endogenous NGG PAM sites (Figure S16). Both editors mediated moderate levels of C‐to‐T editing at hsgRNA loci, with etBE achieving substantially higher editing efficiencies (mean: 10.91% vs. 3.32%) (Figure S17A). The enhanced editing efficiency observed at hsgRNA loci further confirms the improved activity of the mA3CDA1(QM3). Analysis of editing patterns across the protospacer showed that both editors primarily modified cytosines within the C4–C8 region at hsgRNA loci (Figure S17B), likely due to better accessibility of this region. Notably, the average editing efficiencies at hsgRNA loci were significantly lower than those at sgRNA loci for both tBE and etBE (tBE: 3.32% vs. 17.05%; etBE: 10.91% vs. 25.16%) (Figure S17A). The only exception was the C8 position for etBE, where hsgRNA editing slightly exceeded sgRNA editing, possibly owing to the small sample size (*n* = 2). Wang et al. previously demonstrated that truncating the hsgRNA spacer from 20 to 10 nt can effectively eliminate unintended editing at hsgRNA loci without compromising sgRNA editing [16]. To mitigate this undesired editing at hsgRNA loci for etBE, we employed a similar strategy. We compared etBE using 20‐nt and 10‐nt hsgRNA spacers at six individual genomic loci. Shortening the hsgRNA spacer to 10 nt effectively reduced editing at hsgRNA sites (Figure S17C). The average editing efficiency at hsgRNA loci dropped sharply from 6.39% (20‐nt) to 0.05% (10‐nt) (Figure S17D), while base editing efficiencies, indel frequencies, and product purity at the corresponding sgRNA sites remained comparable between the two groups (Figure S17E–G). Allele frequency analysis at the *FANCF* hsgRNA site showed that 10.77% of alleles carried C‐to‐T edits with the 20‐nt hsgRNA, whereas only 0.37% of alleles were edited with the 10‐nt hsgRNA (Figure S17H,I). Overall, these results demonstrate that shortening the hsgRNA spacer is also effective in eliminating undesired editing at hsgRNA loci for etBE.

Subsequently, we parallelly compared etBE and tBE in additional cell lines. Direct comparisons across 17 endogenous NGG PAM loci in HeLa cells confirmed that etBE consistently outperformed tBE, achieving an average editing efficiency of 6.69% versus 2.54% for tBE, while retaining the broadened editing window (Figure 3J and Figure S18A,B). Similar enhancements were observed in Neuro‐2a (N2a) cells (12.41% vs. 5.53%) (Figure 3K and Figure S19A–D). Base editing efficiencies of tBE and etBE groups were substantially lower in both HeLa and N2a cells compared with HEK293T cells, most likely due to their lower transfection efficiency with the dual‐plasmid system. Nevertheless, etBE still conferred significant improvements over tBE in these cell lines, further demonstrating its robustness.

Finally, we compared the base editing efficiencies of tBE, etBE, BE4max, and CBE6a at 11 endogenous genomic loci harboring NGG PAMs in HEK293T cells (Figure S20). Although the BE4max and CBE6a experiments were performed in parallel batches, all constructs were evaluated under identical experimental conditions. NGS analysis revealed that etBE achieved a substantially higher average C‐to‐T editing efficiency than tBE and BE4max (33.58% vs. 23.34% and 16.95%, respectively) and was comparable to CBE6a (27.70%) (Figure 3L). Taken together, these results demonstrate that etBE enables highly efficient base editing across multiple cell lines, comparable to the state‐of‐the‐art editor CBE6a, while maintaining high fidelity.

### 2.6 Evaluation of etBE‐Induced Genomic and Transcriptomic OT Mutations

**FIGURE 4:**
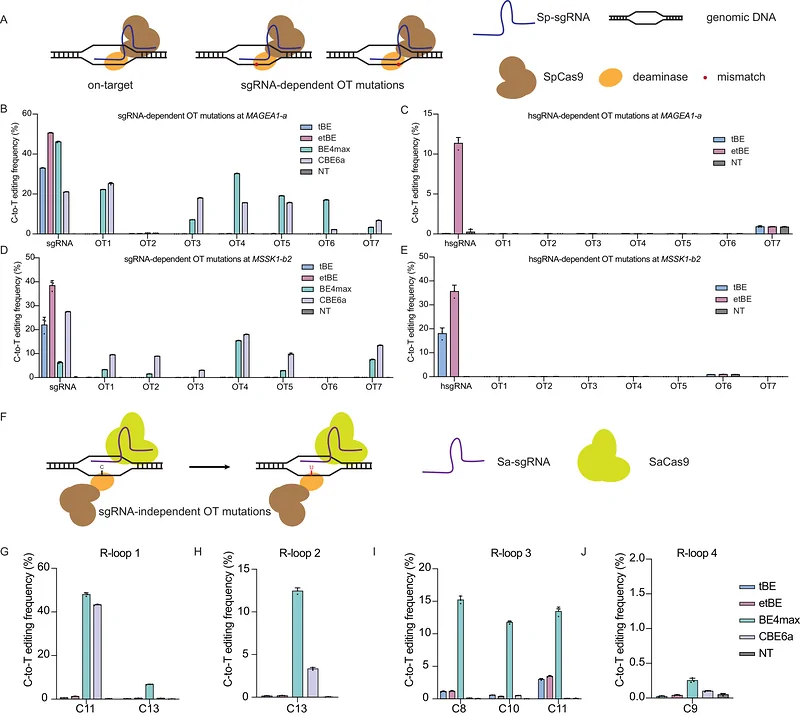
Assessment of etBE‐induced genomic off‐target mutations. (A) Schematic illustration of sgRNA‐dependent off‐target mutations. Genomic DNA is shown in black, SpCas9 protein in brown, SpCas9 sgRNA in navy blue, and the deaminase domain in orange. Mismatches between sgRNA and genomic DNA are highlighted by red solid circles. (B and C) Frequencies of sgRNA‐dependent (B) and hsgRNA‐dependent (C) off‐target mutations induced by tBE, etBE, BE4max, CBE6a, and NT control groups at predicted off‐target sites for the *MAGEA1*‐*a* locus. (D and E) Frequencies of sgRNA‐dependent (D) and hsgRNA‐dependent (E) off‐target mutations induced by tBE, etBE, BE4max, CBE6a, and NT groups at predicted off‐target sites for the *MSSK1*‐*b2* locus. Data represent the mean ± SD from three independent biological replicates. (F) Schematic of the CESSCO assay used to detect sgRNA‐independent off‐target mutations. SaCas9 protein and its corresponding sgRNA are depicted in green‐yellow and purple, respectively. (G–J) Frequencies of sgRNA‐independent off‐target mutations for tBE, etBE, BE4max, CBE6a, and NT groups at R‐loops 1 (G), 2 (H), 3 (I), and 4 (J). Data represent mean ± SD from three independent biological replicates.

To evaluate genomic OT mutations induced by etBE, we employed both sgRNA‐dependent and sgRNA‐independent approaches. sgRNA‐dependent OT mutations occur when SpCas9 tolerates mismatches between the sgRNA and target DNA, enabling editing at loci with high sequence similarity to the on‐target site (Figure 4A). In this study, we compared tBE, etBE, BE4max, CBE6a, and NT groups parallelly at two NGG PAM loci (*MAGEA1*‐*a* and *MSSK1*‐*b2*) and two non‐NGG PAM loci (*CUL3* and *DYRK1A*). Potential OT sites for both sgRNA and hsgRNA were predicted in silico by using CRISPRoff [48], and the top seven were selected for validation. At the *MAGEA1‐a* locus, both tBE and etBE induced only background‐level OT mutations at the tested sgRNA‐dependent predicted sites (average 0.04% and 0.07%, respectively, comparable to 0.06% for the NT control), whereas BE4max and CBE6a mediated substantial editing at six of the seven tested loci (average 14.18% and 11.96%, respectively) (Figure 4B). Notably, BE4max reached up to 30.20% editing at OT4, while CBE6a reached 25.21% at OT1. Moreover, tBE and etBE induced minimal editing at the predicted hsgRNA‐dependent OT loci (Figure 4C). This pattern was recapitulated at other tested loci (Figure 4D,E and Figure S21A–D).

SgRNA‐independent OT mutations arise from deaminase access to transiently exposed single‐stranded DNA (ssDNA) independently of sgRNA guidance (Figure 4F). Here, we utilized the co‐expressing *S. aureus* and *S. pyogenes* Cas9 orthologs (CESSCO) [16, 37] assay, in which orthogonally expressed *S. aureus* Cas9 D9A nickase (nSaCas9) artificially generates R‐loops to mimic accessible ssDNA, to assess sgRNA‐independent OT mutations. Deep sequencing analysis of the four nSaCas9‐induced R‐loops revealed that both tBE and etBE induced significantly lower OT editing than BE4max and CBE6a at R‐loops 1 and 2 (Figure 4G,H). For example, at the C11 position of R‐loop 1, BE4max and CBE6a achieved editing efficiencies of 48.03% and 43.24%, respectively, whereas tBE and etBE induced only 0.61% and 1.23% editing (Figure 4G). At R‐loop 3, tBE and etBE caused modest OT editing (mean: 1.57% and 1.67%, respectively), which was substantially lower than that of BE4max (mean: 13.48%) but higher than CBE6a (0.23%) and the NT control (0.04%) (Figure 4I). All four editors showed only low‐level editing at R‐loop 4 (Figure 4J), possibly due to limited accessibility generated by nSaCas9. Taken together, these results demonstrate that etBE induces minimal genomic OT mutations at the tested loci. Nevertheless, more rigorous and unbiased genome‐wide evaluation methods, such as GOTI [49] and Digenome‐seq [50], could provide more comprehensive insights into the specificity of etBE.

**FIGURE 5:**
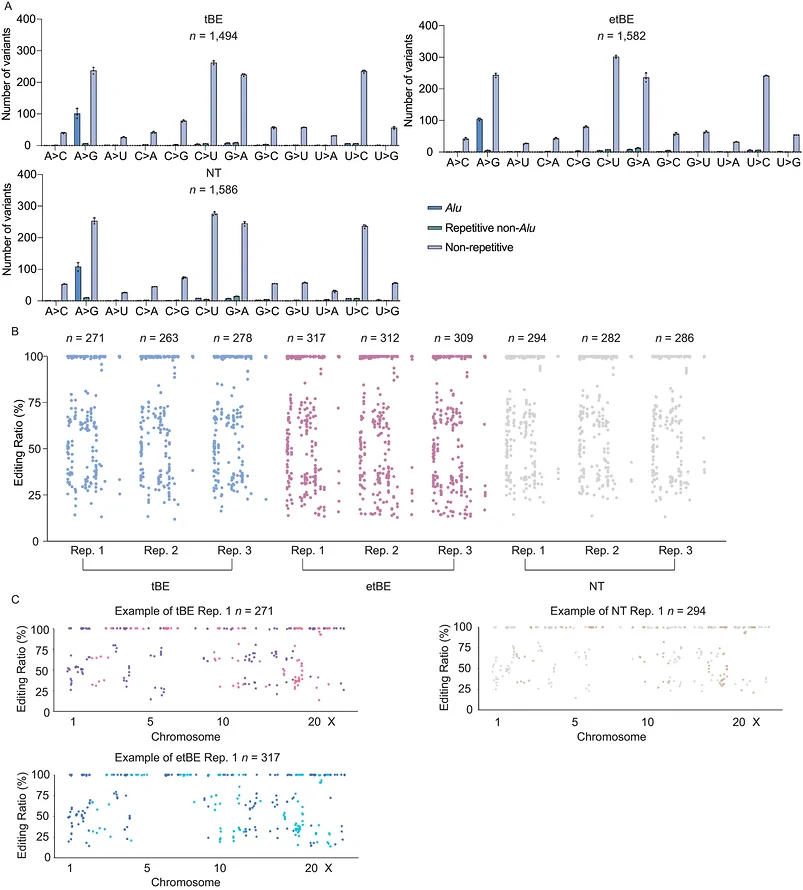
Evaluation of etBE‐induced transcriptomic off‐target mutations. (A) Counts of all 12 types of RNA point mutations detected in HEK293T cells treated with tBE, etBE, or NT control. Data are presented as mean ± SD from three independent biological replicates. (B) Manhattan plot depicting RNA C‑to‑U editing frequencies from the data shown in (A), with counts for each individual replicate labeled. (C) Editing ratios and chromosomal distributions of RNA C‐to‐U variants for tBE, etBE, and NT groups in replicate 1. The full dataset is provided in Figure S22.

To investigate etBE‐induced transcriptomic OT mutations, we co‐transfected HEK293T cells with the dual‐plasmid tBE or etBE system. Cells expressing tBE‐V5‐mA3(WT) or tBE‐V5‐mA3(QM3) were enriched by puromycin selection (4 µg/mL) and harvested 48 h post‐selection, as previously described [16, 37, 42]. RNA‐seq data were analyzed by using the RADAR [16, 37, 42, 44, 51] pipeline. tBE, etBE, and NT control groups were compared in parallel, targeting the *MAGEA1‐a* locus, where we had previously assessed genomic OT mutations. RNA‐seq analysis revealed that both tBE and etBE generated RNA variant levels comparable to the NT control (average 1494 variants for tBE, 1582 for etBE, and 1586 for NT) (Figure 5A). No significant differences were observed between the three groups in the counts of any of the 12 mutation types, including in *Alu*, repetitive non‐*Alu*, and non‐repetitive regions (Figure 5A). We further focused on C‐to‐U RNA mutations. Comparable levels of C‐to‐U variants were detected across tBE (average 271), etBE (313), and NT (287) groups (Figure 5B). Additionally, the chromosomal distribution of C‐to‐U variants showed no specific enrichment (Figure 5C and Figure S22A–C). Collectively, these data demonstrate that etBE induces a background level of transcriptomic OT mutations.

### 2.7 Dual‐AAV‐Mediated In Vivo Base Editing Using etBE in Humanized Mice

**FIGURE 6:**
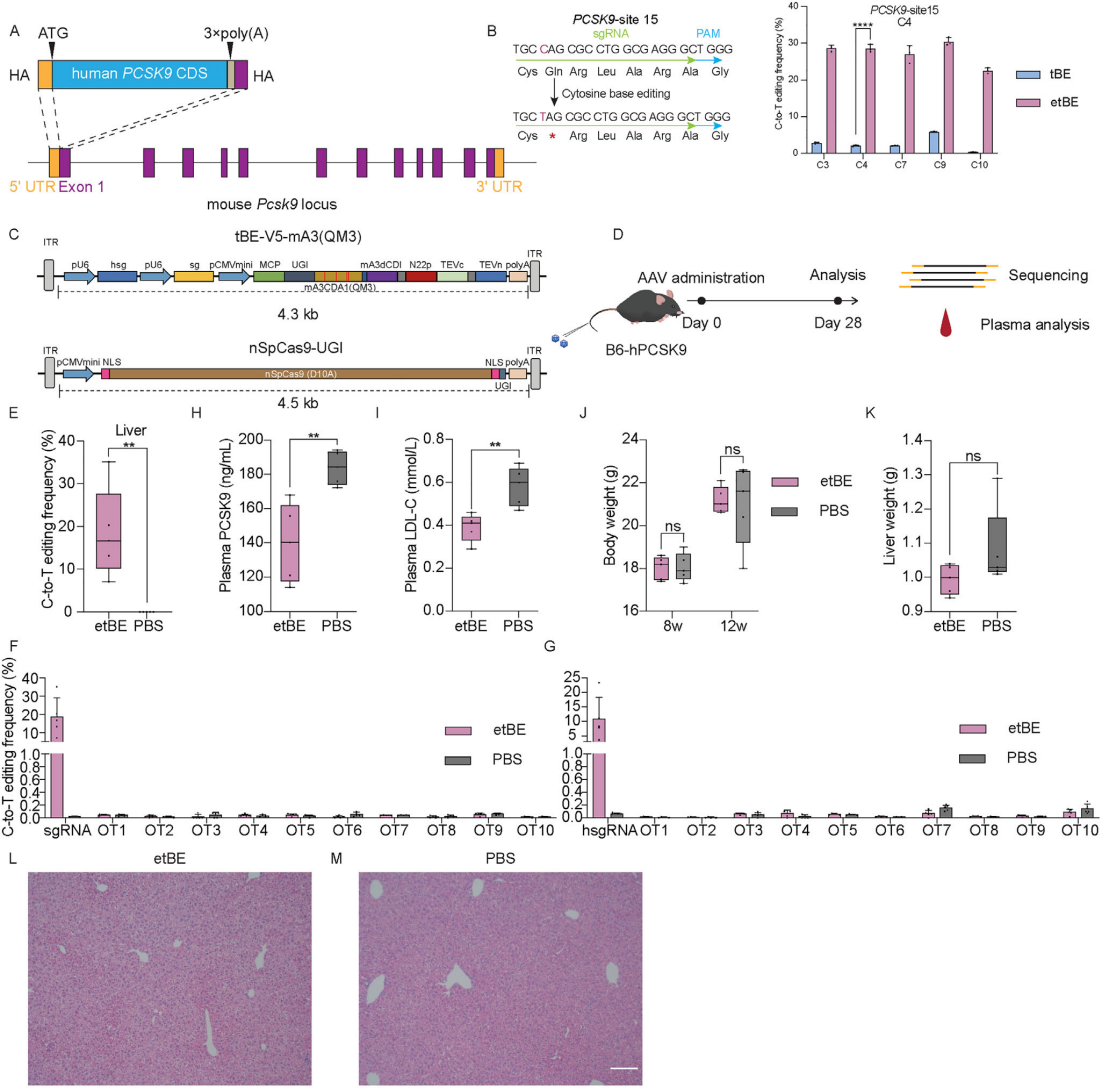
etBE‐mediated in vivo base editing in a humanized mouse model. (A) Schematic illustration of the humanized B6‐hPCSK9 mouse model. The human *PCSK9* coding sequence (CDS) together with a 3 × polyA signal, flanked by left and right homology arms (HAs), was knocked in between the 5ʹ untranslated region (UTR) and the first exon of the endogenous mouse *Pcsk9* locus. (B) Schematic illustration of the target sequence (left) and base editing efficiencies of tBE and etBE at site 15 in the *PCSK9* locus in HEK293T cells (right). The sgRNA sequence is highlighted with green arrows and the PAM sequence with blue arrows. Editing at the C4 position introduces a premature termination codon (highlighted in red). Data are presented as mean ± SD from three independent biological replicates (^****^ *p* < 0.0001). (C) Schematic of the dual‐AAV constructs. The etBE consists of two AAV vectors expressing nSpCas9‐UGI and tBE‐V5‐mA3(QM3), respectively. ITR, inverted terminal repeat; pU6, U6 promoter; hsg, hsgRNA; sg, sgRNA; pCMVmini, mini CMV promoter; MCP, MS2 coat protein; N22p, N peptide from bacteriophage P22; TEVc, C‐terminal fragment of TEV protease; TEVn, N‐terminal fragment of TEV protease; NLS, nuclear localization signal; UGI, uracil DNA glycosylase inhibitor domain. (D) Schematic diagram of the mouse experimental design. Eight‐week‐old female B6‐hPCSK9 mice received intravenous injection of dual‐AAV vectors at a dose of 2 × 10^14^ vg/kg or an equal volume of PBS as a control. Mice were sacrificed 4 weeks post‐injection. (E) Base editing efficiency of etBE and PBS groups in mouse liver. Data represent mean ± SD from 5 mice (^**^ *p* < 0.01). (F and G) Base editing frequencies at sgRNA‐dependent (F) and hsgRNA‐dependent (G) off‐target loci in mouse liver. Data represent mean ± SD from five mice. (H and I) Plasma PCSK9 (H) and LDL‐C (I) levels in etBE‐treated and PBS control groups (^**^ *p* < 0.01). (J) Body weight of mice at 8 and 12 weeks of age (ns, not significant). (K) Liver weight of mice at 12 weeks of age (ns, not significant). (L and M) Histological analysis of liver sections from etBE‐treated (L) and PBS control (M) mice. Scale bar, 100 µm. Data represent the mean ± SD from five mice. Statistical significance in Figure 6B,E,H–K was determined by an unpaired two‐tailed Student's *t*‐test.

To fully evaluate the in vivo base editing potential of etBE, we selected the *PCSK9* as the target gene, a well‐established therapeutic target for cardiovascular diseases [52, 53]. To better recapitulate the clinical setting, we utilized a humanized B6‐hPCSK9 mouse model in which the complete human *PCSK9* CDS, followed by a 3 × poly(A) signal and flanked by left and right homology arms (HAs), was knocked in between the 5ʹ untranslated region (UTR) and the first exon of the endogenous mouse *Pcsk9* locus (Figure 6A).

To achieve efficient in vivo base editing, we designed seven sgRNAs targeting the first six exons of the human *PCSK9* CDS using BE‐Designer [54]. Corresponding hsgRNAs were designed according to the previously reported protocol [16, 37, 42, 43] and positioned 40–90 bp upstream of each sgRNA. In total, we generated 17 hsgRNA‐sgRNA pairs (denoted as sites 1–17 hereafter) and compared the editing efficiencies of tBE and etBE side‐by‐side in HEK293T cells. NGS analysis showed that etBE outperformed tBE at 14 of the 17 tested loci. The full dataset, including all efficiently edited cytosines, is shown in Figure S23, with those capable of generating premature termination codons (PTCs) specifically indicated. Notably, etBE achieved markedly higher editing efficiency than tBE at site 15 (C4 position: 28.13% vs. 2.00%) (Figure 6B). Given the low editing efficiency of tBE, only the etBE system was advanced to in vivo studies. Since the etBE system exceeds the packaging capacity of a single AAV, we employed a dual‐AAV delivery strategy (Figure 6C). One AAV encodes the nSpCas9‐UGI fusion protein, while the other carries the tBE‐V5‐mA3(QM3) construct. AAV8 serotype was chosen for its high liver tropism, low immunogenicity, and sustained expression [16, 55, 56]. Eight‐week‐old female B6‐hPCSK9 mice were intravenously injected with dual AAVs at a total dose of 2 × 10^14^ vector genome (vg)/kg (experimental group) or with an equal volume of phosphate‐buffered saline (PBS) as the control group (Figure 6D). Mice were sacrificed four weeks post‐injection. Livers and plasma were harvested for subsequent analyses. Deep sequencing revealed that etBE achieved efficient base editing in the liver, with an average efficiency of 18.45% (7.06%–35.13%), which was significantly higher than that in the PBS control group (average 0.02%) (Figure 6E), confirming the in vivo targeting potential.

We next assessed the specificity of etBE by examining the top 10 predicted OT sites for both hsgRNA and sgRNA. Only background‐level mutations were detected at sgRNA‐ and hsgRNA‐dependent OT loci (Figure 6F,G), demonstrating high in vivo specificity. Notably, etBE achieved an average editing efficiency of 10.86% at hsgRNA loci, consistent with our previous findings (Figures S16 and S17). The selected sgRNA contains three mismatches with the mouse *Pcsk9* homolog at OT4 (human: TGCCAGCGCCTGGCGAGGGC**TGG**; mouse: TGCCgGCaCCTGGCGAGGaC**TGG**, mismatches in lowercase, PAM in bold). No detectable editing was observed at this site. Similarly, the hsgRNA used in this study contains two mismatches with the mouse *Pcsk9* homolog. However, this locus ranked low in the OT prediction (OT23) and was therefore not included among the top 10 predicted sites that we examined. The low prediction score is likely attributable to a mismatch in the PAM‐proximal region (human: TGCTGCTGCCCCTGGCGGGT**GGG**; mouse: TtCTGCTGCCCCTGGCcGGT**GGG**). Collectively, these results demonstrate the high in vivo specificity of etBE.

Subsequently, phenotypic analysis revealed a significant reduction in plasma PCSK9 levels in etBE‐treated mice compared with the PBS control group (139.8 ng/mL vs. 183.7 ng/mL), accompanied by decreased LDL‐C levels (0.39 mmol/L vs. 0.582 mmol/L) (Figure 6H,I). No significant differences were observed in body weight (Figure 6J) or in liver weight (Figure 6K). Histological examination of liver sections showed no apparent abnormalities or differences between the two groups (Figure 6L,M). Overall, these results demonstrate that etBE‐mediated *PCSK9* editing effectively reduced plasma PCSK9 and LDL‐C levels, underscoring its therapeutic potential.

## 3 Discussion

In this study, we identified 18 beneficial amino acid substitutions in mA3CDA1 that enhance deaminase activity through structure‐guided and MSA‐based rational protein engineering (Figure 1B,C). Through iterative combinatorial optimization of the six top‐performing mutations, we generated the quadruple mutant mA3CDA1(QM3), which substantially improved base editing efficiency at majority of the tested genomic sites (Figure 2C,D). Although mA3CDA1(QM3) initially elevated editing byproducts, fusion of a single UGI domain to the C‐terminus of nSpCas9 effectively restored editing fidelity (Figure 3B–D). The resulting etBE, comprising nSpCas9‐UGI and tBE‐V5‐mA3(QM3), exhibited markedly higher C‐to‐T editing efficiency, an expanded editing window, improved product purity, and comparable indel rates (Figure 3E–I). Notably, etBE exhibited minimal genomic OT mutations at tested loci (Figure 4) and background‐level transcriptomic OT mutations (Figure 5). Furthermore, dual‐AAV delivery of etBE achieved efficient in vivo editing in a humanized mouse model, resulting in significant reductions in plasma PCSK9 and LDL‐C levels (Figure 6). Collectively, these findings establish etBE as a highly efficient and secure base editing platform with considerable potential for both basic research and therapeutic applications.

Here, we engineered the mA3CDA1 deaminase using a combination of structure‐guided and MSA‐based rational design approaches. Guided by the AlphaFold 2‐predicted structure, we identified key residues located in close proximity to the substrate DNA and introduced positively charged amino acids at these positions. We reasoned that cytosine deamination is a multi‐turnover enzymatic process, such that increasing the binding affinity between the deaminase and substrate DNA would prolong the residence time on the target site and thereby enhance base editing efficiency. We substituted selected residues with positively charged amino acids to strengthen electrostatic interactions with the negatively charged DNA backbone. This strategy is supported by previous studies showing that arginine scanning can improve nuclease activity by enhancing substrate binding affinity [57, 58, 59], and that fusion of ssDNA‐binding domains [31, 60, 61] or RNA‐DNA hybrid binding domains [62] can similarly elevate base editor efficiency. More recently, Kweon et al. enhanced the activity of a DYW‐like deaminase derived from the toxin of *Pseudomonas syringae* by introducing positively charged residues (V289H/K/R, H333R, and Y335R) [35]. These findings collectively validate our hypothesis and rational design approach.

The AlphaFold‐predicted structure provided direct insights into the interaction between mA3CDA1 and substrate DNA, enabling the identification of key residues within the catalytic pocket. However, static structural models do not involve all relevant dynamic conformational states. We therefore complemented the structure‐based design with MSA of mA3CDA1 and other active CDAs. This strategy prioritizes substitutions that naturally occur in functional orthologs, thereby reducing the risk of introducing mutations that compromise protein folding or stability [29]. Of the 18 beneficial substitutions, 16 candidates were identified through structure‐guided design while two through MSA. Notably, the D136N mutation in mA3CDA1 corresponds to the D124N substitution previously shown to enhance deaminase activity in evoBE4max [19], underscoring the structural and functional conservation among CDAs. Several other beneficial substitutions identified here have not been previously reported, underscoring the value of exploring mA3CDA1. Although our design efforts primarily focused on the catalytic pocket, we also screened 38 additional candidate mutations located outside this region via MSA analysis. None of these peripheral mutations significantly improved deaminase activity. This does not imply that residues outside the active‐site pocket are dispensable. Instead, they possibly play critical roles in modulating substrate access and protein conformational dynamics. For instance, a recent study on the prime editor (PE) [63] system showed that AI‐guided optimization of residues outside the active pocket, aimed at improving protein stability and expression, substantially enhanced editing efficiency. However, the rational design strategy employed in this study was inherently limited by its relatively low throughput and primary focus on the core catalytic region. High‐throughput approaches, such as saturated mutagenesis [64, 65], directed evolution [66, 67], and AI‐assisted screening [63], could serve as valuable complements to the rational design.

The etBE system achieves substantially higher C‐to‐T editing efficiency than the parental tBE, albeit with a broader editing window and consequently elevated bystander mutation risk. Allele frequency analysis revealed that the proportion of alleles carrying only the intended single‐nucleotide substitution was low for both editors (1.39% for tBE and 0.29% for etBE at the *DNMT3B* locus; 2.05% for tBE and 0.95% for etBE at the *MAGEA1‐a* locus) (Figure S24A–D), with etBE showing even lower levels of precise single editing. This is mechanistically expected, as the engineered mA3CDA1(QM3) variant possesses enhanced substrate‐binding affinity and increased processivity, allowing the deaminase to act on both the target cytosine and nearby bystander cytosines during the multi‐turnover reaction. A similar pattern is also observed for mA3CDA1(WT)‐BE4max, mA3CDA1(QM3)‐BE4max, rA1‐BE4max, and CBE6a at the same *MAGEA1‐a* locus (Figure S24E–H), indicating that bystander editing remains a common challenge for many base editors. Nevertheless, etBE remains highly advantageous for gene disruption applications, particularly in applications where safety is of paramount importance, such as those requiring the avoidance of DSBs and minimization of OT mutations. Overall, etBE offers a useful tool for applications requiring precise C‐to‐T transitions, serving as both an alternative and a complement to prime editors (PEs).

Similar trade‐offs between editing efficiency and window width have been commonly observed in other highly active base editors [31, 60, 68, 69]. Conversely, base editors engineered for narrower editing windows often exhibit reduced on‐target efficiency [21, 70, 71, 72]. We also attempted to narrow the editing window of tBE. However, none of the tested mutants achieved a satisfactory balance between precision and efficiency. For example, the W102Y mutation in mA3CDA1 (corresponding to W90Y in BE3 [21]) significantly decreased editing efficiency at the *DNMT3B* locus (e.g., 51.76% vs. 71.85% at C7) (Figure S3C). Similarly, the Y132A and Y132G substitutions in mA3CDA1 (corresponding to the Y130A and Y130G mutations in highly accurate hA3A‐based CBE [22]) essentially abolished deaminase activity (1.84% and 1.20% at C7, respectively) (Figure S3D). Combinatorial mutants also showed only marginally detectable activity. These data suggest that simply transplanting mutations from other CDA orthologs may not be directly applicable to mA3CDA1. Recently, several promising strategies have been reported to reduce bystander editing while maintaining high on‐target efficiency [73, 74, 75], providing valuable directions for future optimization of the tBE system.

Due to the limited packaging capacity of a single AAV vector, the tBE system necessitates a dual‐AAV delivery strategy. Although the elegant architecture of tBE was originally developed by Wang et al. [16]. obviates the need for trans‐splicing systems, dual‐AAV delivery can still reduce overall editing efficiency in vivo due to lower co‐transduction rates, potentially underestimating its therapeutic efficacy. Further optimization of delivery modalities is expected to improve in vivo base editing performance. For instance, emerging platforms with greater cargo capacities, such as lipid nanoparticles (LNPs) [76, 77] and virus‐like particles (VLPs) [78, 79], have demonstrated tremendous promise in in vivo gene delivery and could significantly enhance the therapeutic application of tBE in the future.

The reduction in plasma PCSK9 and LDL‐C levels observed in this study was more modest than those reported in previous studies [80, 81], likely due to the relatively moderate in vivo editing efficiency achieved (mean: 18.45%). In addition, the baseline plasma PCSK9 concentration in the humanized B6‐hPCSK9 mouse model was relatively low (183.7 ng/mL) compared with WT C57BL/6J mice (approximately 350 ng/mL [81]), which may have led to an underestimation of the therapeutic effect. Further studies are warranted to clarify the physiological differences between these models.

## 4 Methods

### 4.1 sgRNA Design and Off‐Target Prediction

The sgRNAs employed in this study were computationally designed using BE‐Designer (http://www.rgenome.net/be‐designer) [54] under default parameters to optimize editing efficiency. Off‐target predictions were performed against the hg38 (human) and mm10 (mouse) reference genomes using CRISPRoff (https://rth.dk/resources/crispr/crisproff/) [48]. Sequencing primers for the target loci were designed via Primer3 (https://primer3.ut.ee/) [82] and commercially synthesized by BiOligo (Shanghai, China). Complete details of the constructs, cloning oligonucleotides, hsgRNA and sgRNA sequences, PAM motifs, predicted off‐target sites, and sequencing primers are presented in Tables S1–S3.

### 4.2 Cloning

The plasmids pEFS‐nSpCas9 (D10A) (Addgene #171691), pEFS‐nSpCas9 (D10A)‐NG (Addgene #171692), ccdB‐Sp‐sgRNA scaffold‐boxB‐tBE‐V5‐mA3 (Addgene #171693), pUC57‐sgRNA‐MS2‐pU6 (Addgene #171694), and pU6‐Sa‐sgRNA scaffold‐pCMV‐nSaCas9 (D9A) (Addgene #171695) were obtained as gifts from Jia Chen [16]. The pEFS‐nSpRY (D10A) construct was generated from pEFS‐nSpCas9 (D10A)‐NG by sequential introduction of mutations via PCR. All tBE‐V5‐mA3 vectors were constructed as previously described, with a detailed protocol available on Protocol Exchange [16, 42].

All deaminase mutants were generated using the KOD‐Plus‐Mutagenesis Kit (TOYOBO, SMK‐101) following the manufacturer's instructions. Briefly, inverse PCR was performed with 10 cycles using primers incorporating the desired mutations, followed by *DpnI* digestion of the template plasmid DNA. The PCR products were then phosphorylated with T4 polynucleotide kinase at 16°C for 1 h and transformed into DH5α competent cells (Tolobio, CC96102‐01).

The pCMV_BE4max plasmid (Addgene #112093) was a gift from Yongming Wang. The rA1‐BE4max expression construct used in this study was derived from pCMV_BE4max by replacing the CMV promoter with the EFS promoter and inserting a pU6‐driven sgRNA expression cassette and a pSV40‐driven puromycin resistance gene cassette via Gibson Assembly (NEB, E2621L). Subsequent all‐in‐one vectors, including mA3CDA1(WT)‐BE4max, mA3CDA1(QM3)‐BE4max, mA3CDA1(WT)‐mA3dCDI‐BE4max, and mA3CDA1(QM3)‐mA3dCDI‐BE4max, were constructed from this modified rA1‐BE4max backbone using Gibson Assembly. Briefly, the backbone was amplified by inverse PCR using KOD‐Plus‐Neo polymerase (TOYOBO, KOD‐401), while insert fragments were PCR‐amplified from the ccdB‐Sp‐sgRNA scaffold‐boxB‐tBE‐V5‐mA3 plasmid (Addgene #171693) or from deaminase mutants generated in this study. The CBE6a all‐in‐one construct was generated by replacing rA1 in rA1‐BE4max with the TadA‐derived cytidine deaminase via Gibson Assembly. For sgRNA spacer integration, complementary oligonucleotides containing BbsI‐compatible overhangs (CACC on the forward strand and AAAC on the reverse strand) were annealed by heating to 95°C and slowly cooling to room temperature, then ligated into BbsI‐HF‐digested (NEB, R3539L) all‐in‐one vectors using T4 DNA ligase (NEB, M0202L).

For the CESSCO assay, oligonucleotides with compatible overhangs (ACCG on the forward strand and AAAC on the reverse) were annealed and ligated into BsmBI‐digested (NEB, R0739L) pU6‐Sa‐sgRNA scaffold‐pCMV‐nSaCas9 (D9A) vectors. All plasmid constructs were verified by Sanger sequencing. The sequences of all oligonucleotides used for cloning are listed in Table S1.

### 4.3 Cell Culture and Transfection

All mammalian cell lines were maintained in a humidified incubator at 37°C and 5% CO_2_. HEK293T (ATCC CRL‐11268), HeLa (ATCC CCL‐2), and N2a (ATCC CRL‐131) cells were cultured in Dulbecco's Modified Eagle's Medium (DMEM, VivaCell, C3122‐0500) supplemented with 10% fetal bovine serum (FBS, VivaCell, C04000‐500) and 100 U/mL penicillin‐streptomycin (Biosharp, BL505A). Cells were routinely screened for mycoplasma contamination using a commercial detection kit (Takara, CY232) and confirmed to be mycoplasma‐free.

For tBE experiments, cells were seeded into 24‐well plates (WHB Scientific, WHB‐24) at a density of 8 × 10^4^ cells per well 16 h prior to transfection. Transfections were performed with 500 ng each of nSpCas9 and tBE‐V5‐mA3 plasmids (or tBE‐V5‐mA3 variants) complexed with 3 µL polyethylenimine (PEI, Polysciences, 24765). Puromycin (LABLEAD, P0671) was added to a final concentration of 4 µg/mL 24 h post‐transfection. Cells were harvested 72 h post‐transfection for downstream analyses. For base editing with all‐in‐one vectors, including rA1‐BE4max, CBE6a, mA3CDA1(WT)‐BE4max, mA3CDA1(QM3)‐BE4max, mA3CDA1(WT)‐mA3dCDI‐BE4max, and mA3CDA1(QM3)‐mA3dCDI‐BE4max, 500 ng plasmid was diluted in 50 µL serum‐free DMEM containing 1.5 µL PEI. The mixture was incubated for 10 min at room temperature before addition to the cell cultures. For the CESSCO assay, an additional 500 ng nSaCas9 expression plasmid was co‐transfected.

### 4.4 Next‐Generation Sequencing

Harvested cells were lysed using QuickExtract DNA Extraction Solution (Lucigen, QE0905T) at 65°C for 15 min, followed by inactivation at 98°C for 2 min. Target sites were amplified via two rounds of PCR using KOD One PCR Master Mix‐Blue (TOYOBO, KMM‐201). Sequencing primers were incorporated in the first round, while indices and adaptors were added in the second round. Amplicons were verified by electrophoresis on 2% agarose gels (Biosharp, BS081‐100 g), with bands of expected size extracted and purified using a column‐based spin kit (Tiangen, DP214). Purified DNA amplicons were sequenced on the Illumina NovaSeq X Plus platform (Sequenta, Shanghai, China) in 150‐bp paired‐end mode. Targeted deep‐sequencing data were analyzed using CRISPResso2 (version 2.2.14) [30] with default parameters. PCR primer sequences are listed in Table S2.

### 4.5 Structure Analysis

The structure of mA3CDA1 deaminase was predicted using AlphaFold 2 (https://alphafold.com) [83] with default parameters. Crystal structures of human APOBEC3A in complex with substrate DNA (PDB ID: 5SWW) [26], human APOBEC3G (PDB ID: 3EIU) [27], and the predicted mouse APOBEC deaminase structure were analyzed using UCSF ChimeraX (version 1.7rc202312120032) [84].

### 4.6 Transcriptome Sequencing and RNA Editing Analysis

HEK293T cells were seeded into 12‐well plates (WHB Scientific, WHB‐12) at a density of 2 × 10^5^ cells per well 16 h prior to transfection. Six different mixtures were prepared as follows: (1) 1 µg nSpCas9 + 1 µg tBE‐V5‐mA3(WT), (2) 1 µg nSpCas9‐UGI + 1 µg tBE‐V5‐mA3(QM3), (3) 1 µg mA3CDA1(WT)‐BE4max, (4) 1 µg mA3CDA1(QM3)‐BE4max, (5) 1 µg mA3CDA1(WT)‐mA3dCDI‐BE4max, or (6) 1 µg mA3CDA1(QM3)‐mA3dCDI‐BE4max. Each mixture was diluted in 100 µL serum‐free DMEM containing 6 µL PEI. Puromycin selection (4 µg/mL) was initiated 24 h post‐transfection and continued for 48 h. Total RNA was isolated from HEK293T cells using TRIzol reagent (Thermo Fisher Scientific, 15596026CN) per the manufacturer's protocol. RNA concentration was quantified using a NanoDrop 2000 spectrophotometer (Thermo Fisher Scientific), and integrity was evaluated by capillary electrophoresis on an Agilent 2100 Bioanalyzer. Only samples meeting the following criteria were used for library preparation: total RNA ≥ 2 µg, concentration ≥ 50 ng/µL, volume ≥ 10 µL, OD260/280 = 1.8–2.0, OD260/230 = 1.8–2.2, RIN ≥ 7, and 28S/18S ratio ≥ 1. Strand‐specific RNA‐seq libraries were prepared using the VAHTS Universal V6 RNA‐seq Library Prep Kit for Illumina (Vazyme, NR604‐02). Briefly, mRNA was enriched and purified using oligo(dT) magnetic beads, followed by fragmentation. First‐strand cDNA was synthesized by reverse transcription, and second‐strand cDNA synthesis was coupled with end repair and dA‐tailing. Indexed adaptors were ligated, and libraries were size‐selected (∼260–280 bp) using VAHTS DNA Clean Beads (Vazyme, N411‐03). Libraries were amplified by PCR (15–18 cycles) and purified. Library quality and quantification were assessed using Qsep‐400 (BiOptic) and Qubit 3.0 (Thermo Fisher Scientific), respectively. Sequencing was performed on the Illumina NovaSeq X Plus platform in paired‐end 150 bp mode by Tsingke (Beijing, China). RNA variants were identified and visualized using the RADAR pipeline (https://github.com/YangLab/RADAR; v1.0.0) [16, 42, 44, 51], filtering for sequencing depth > 150, RNA editing ratio ≥ 0.05, variant quality > 150, quality by depth > 2, Fisher strand bias ≤ 30, and mapping quality ≥ 20. A detailed protocol for transcriptome‐wide OT mutation analysis is available on Protocol Exchange [42].

### 4.7 Hematoxylin and Eosin Staining

Tissues were fixed in 4% paraformaldehyde (Servicebio, G1101‐500ML) at 4°C overnight, followed by paraffin embedding the next day. Sections were cut at 5 µm thickness from paraffin blocks, deparaffinized, and stained with hematoxylin and eosin. Histological differences were examined using a Nikon ECLIPSE Ni‐U microscope.

### 4.8 Animal Experiments

C57BL/6J mice were purchased from JSJ‐Lab (Shanghai, China), while humanized B6‐hPCSK9 C57BL/6J mice were obtained from Gempharmatech (Nanjing, China). All mice were housed in a pathogen‐free facility at Fudan University under a 12‐h light/12‐h dark cycle, at 20°C–22°C and 40%–60% humidity. Eight‐week‐old female B6‐hPCSK9 mice were injected intravenously with 200 µL PBS or AAVs. Four weeks post‐injection, mice were fasted for 6 h, after which blood was collected. Hearts, livers, spleens, kidneys, and lungs were harvested following PBS perfusion for downstream analyses. All mouse experiments were approved by the Institutional Animal Care and Use Committee of Fudan University (2024JS076) and conducted in accordance with institutional guidelines.

### 4.9 Plasma Collection and Biochemical Analysis

Blood was collected from mice via terminal cardiac puncture into heparinized tubes at 4 weeks post‐injection. Plasma was separated by centrifugation and stored at −80°C until further analysis. Plasma PCSK9 concentrations were measured using a commercial ELISA kit (MULTI SCIENCES, EK1124). LDL‐C levels were determined by a contracted clinical laboratory service (YuxiuBio, Shanghai, China).

### 4.10 Western Blot

Cells were harvested and lysed 48 h post‐transfection using RIPA lysis buffer (Epizyme, PC101) supplemented with a protease inhibitor cocktail (Epizyme, GRF101) on ice for 15 min. The lysates were centrifuged at 12 000 rpm for 15 min at 4°C, and the supernatant was collected. Proteins were separated by SDS‐PAGE and subsequently transferred onto PVDF membranes (Immobilon, ISEQ00010). The membranes were blocked with 5% skim milk (BD Bioscience, 232100) and then probed with primary antibodies against FLAG (Abmart, M20008S) and β‐actin (ABclonal, AC004). After incubation with HRP‐conjugated secondary antibodies (ABclonal, AS003), protein signals were visualized using an enhanced chemiluminescence substrate.

### 4.11 Statistics Analysis and Reproducibility

The sample size (*n*) for each experiment is indicated in the figure legends. All statistical analyses were performed with GraphPad Prism software (version 10.1.1). Differences were assessed using an unpaired, two‐tailed Student's *t*‑test or one‑way ANOVA followed by Tukey's multiple comparisons test, as appropriate. *p* value < 0.05 was considered statistically significant, and significance levels are denoted as follows: ^*^ *p* < 0.05, ^**^ *p* < 0.01, ^***^ *p* < 0.001, and ^****^ *p* < 0.0001. All experiments were independently repeated at least twice to ensure reproducibility.

## Author Contributions

D.L. and H.C. conceived and designed the study. B.C., R.X., and T.W. designed constructs and conducted cell experiments. B.C. performed bioinformatic analyses. B.C. and L.L. performed mouse experiments and wrote the manuscript. All authors reviewed and approved the final version.

## Conflicts of Interest

The authors declare no conflicts of interest.

## Supporting information




**Supporting File 1**: advs77657‐sup‐0001‐SuppMat.pdf.

**Supporting File 2**: advs77657‐sup‐0002‐S1.xlsx.

**Supporting File 3**: advs77657‐sup‐0003‐S2.xlsx.

**Supporting File 4**: advs77657‐sup‐0004‐S3.xlsx.

## Data Availability

The data that supports the findings of this study are available in the supplementary material of this article. Deep sequencing data generated in this study have been deposited in the National Center for Biotechnology Information Sequence Read Archive under Bioproject “PRJNA1522987”.
