# Supplementary material for: Engineered Transformer Base Editor with Enhanced Editing Efficiency
Source: Adv Sci (Weinh). 2026 Sep 10:e77657. Online ahead of print. doi: 10.1002/advs.77657 (PMC13562778; doi:10.1002/advs.77657)
Supplement: Supplementary file 1 [file ADVS-9999-e77657-s001.pdf]

Figure S1

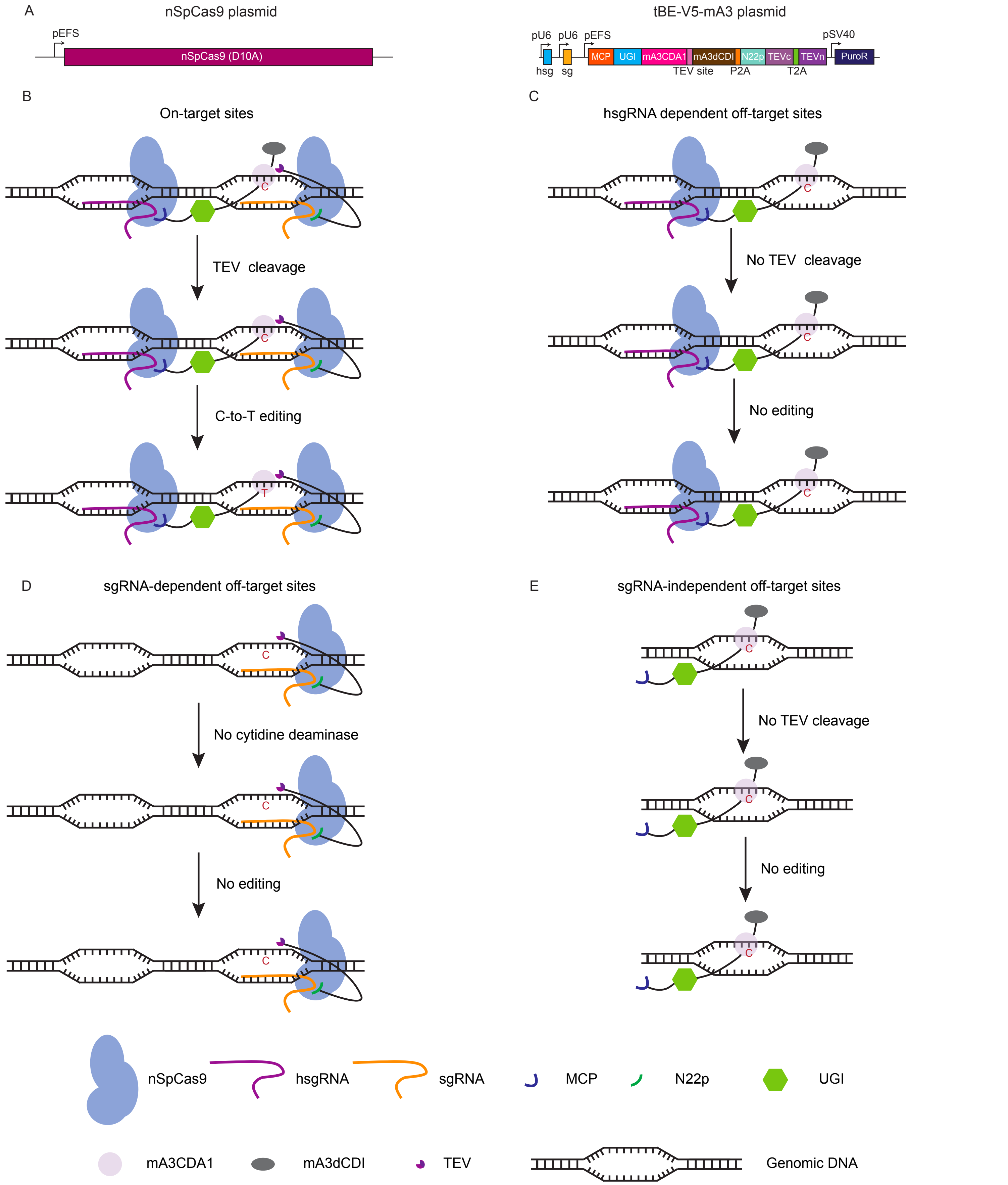

Figure S1. Schematic illustration of the tBE system. (A) Architectures of the dual plasmids for tBE, including the nSpCas9-expressing plasmid and the tBE-V5-mA3 plasmid. pEFS, EFS promoter; pU6, U6 promoter; hsg, hsgRNA; sg, sgRNA; MCP, MS2 coat protein; UGI, Uracil DNA glycosylase inhibitor; N22p, N peptide from bacteriophage P22; pSV40, SV40 promoter; PuroR, puromycin resistance gene; P2A/T2A, self-cleaving peptide. (B) Schematic of tBE-mediated C-to-T editing at on-target sites. (C–E) Schematics illustrating how tBE maintains high specificity at hsgRNA-dependent off-target sites (C), sgRNA-dependent off-target sites (D), and sgRNA-independent off-target sites (E). Only at on-target sites, where both hsgRNA and sgRNA bind, does TEV protease-mediated cleavage of mA3dCDI activate tBE, thereby catalyzing cytosine-to-thymine conversion.

Figure S2

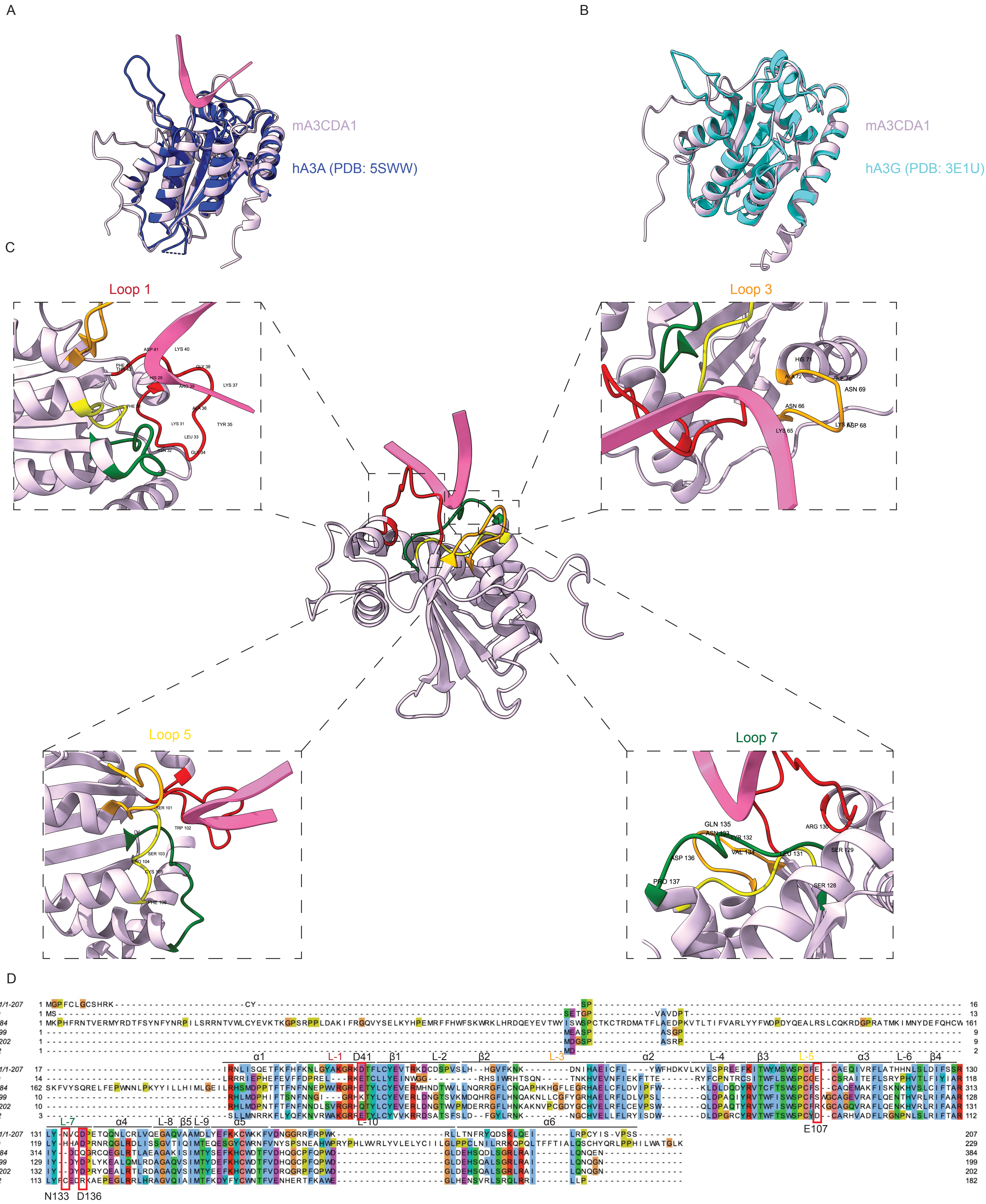

Figure S2. AlphaFold 2-predicted structure of mA3CDA1 and multiple sequence alignment of CDAs. (A) Structural superposition of the AlphaFold 2-predicted mA3CDA1 structure (pink) and the crystal structure of human APOBEC3A (A3A) (PDB ID: 5SWW, navy blue). The bound substrate DNA is shown in magenta. (B) Structural superposition of the predicted mA3CDA1 structure (pink) and the crystal structure of human APOBEC3G (A3G) (PDB ID: 3E1U, cyan). (C) Predicted mA3CDA1 structure generated by AlphaFold 2, highlighting the four loops that form the substrate-binding groove. Loops 1, 3, 5, and 7 are colored red, orange, yellow, and green, respectively, with an enlarged view showing labeled amino acid residues. (D) Multiple sequence alignment of mA3CDA1 with rat APOBEC1 (rA1), hA3G, hA3A, macaca fascicularis APOBEC3A (mfA3A), and human activation-induced cytidine deaminase (AID). Secondary structures are indicated by horizontal black lines above the sequence. Loops 1, 3, 5, and 7 are highlighted in red, orange, yellow, and green, respectively. The four amino acid substitutions introduced in mA3CDA1(QM3) are highlighted with red boxes.

Figure S3

A Loop 1

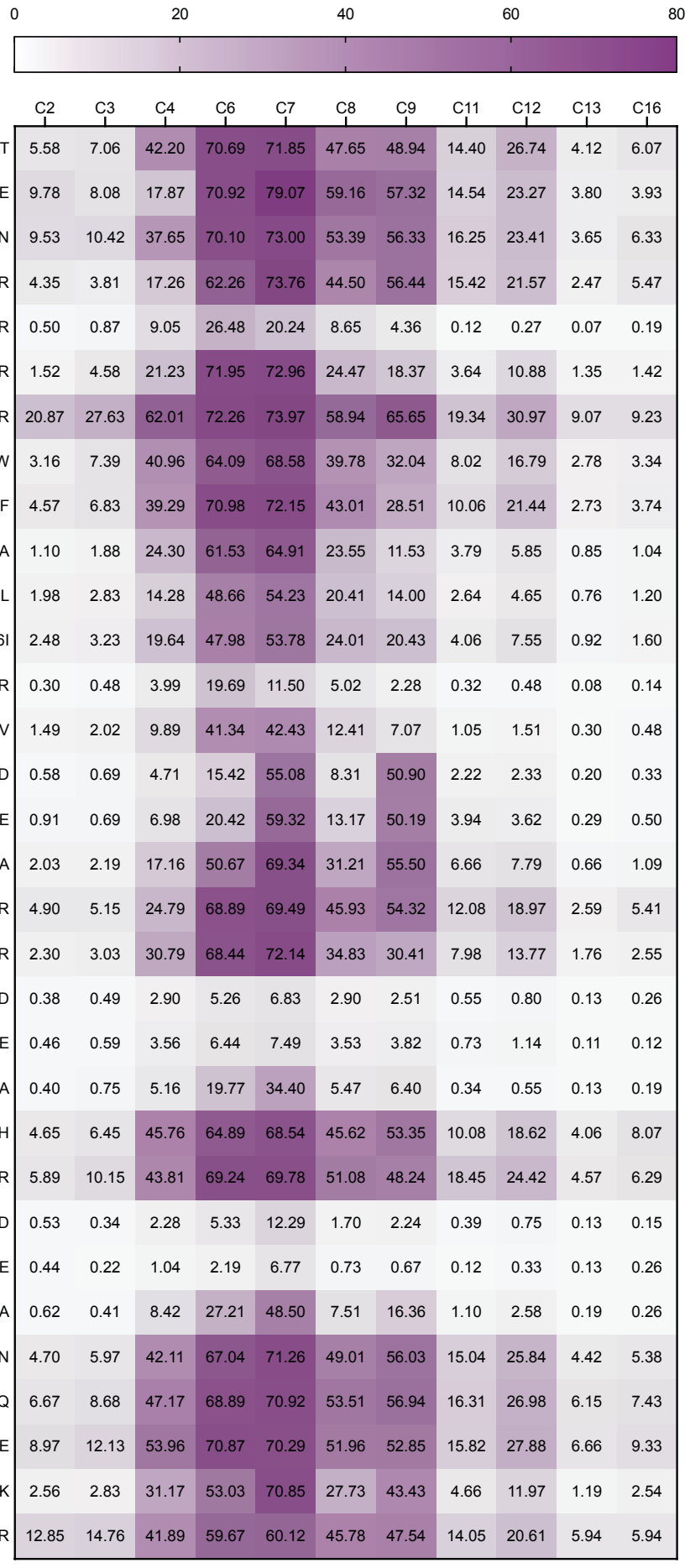

B Loop 3

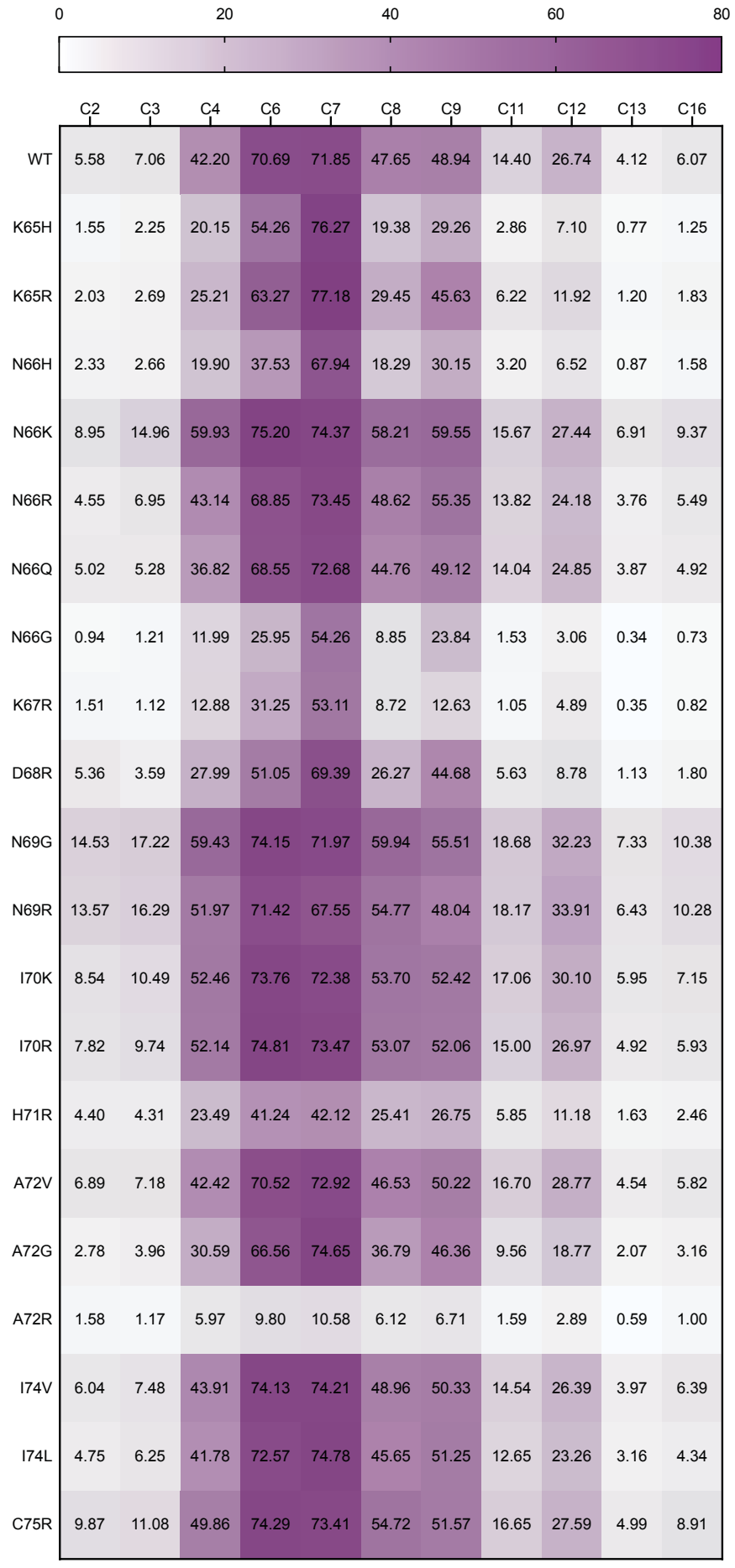

C Loop 5

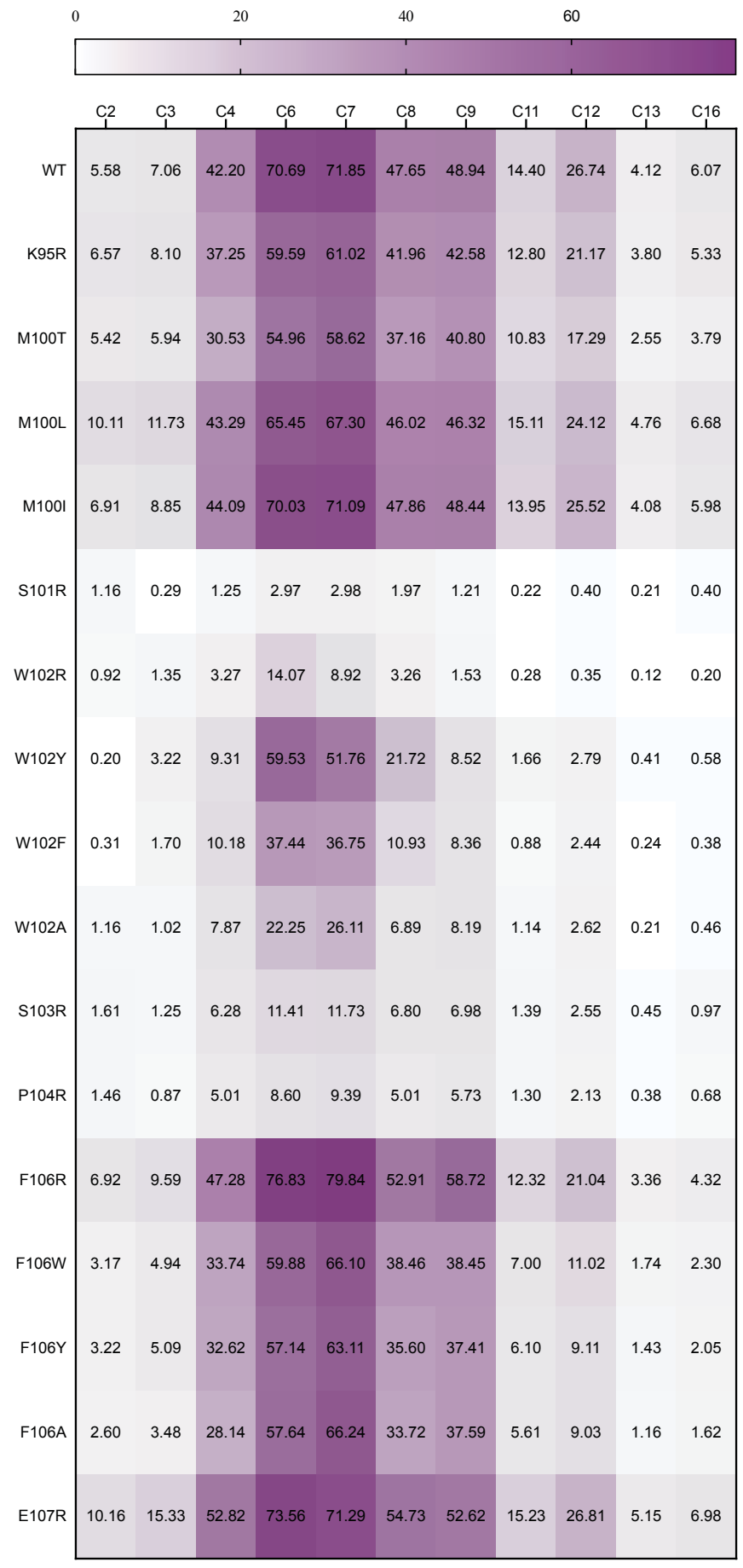

D Loop 7

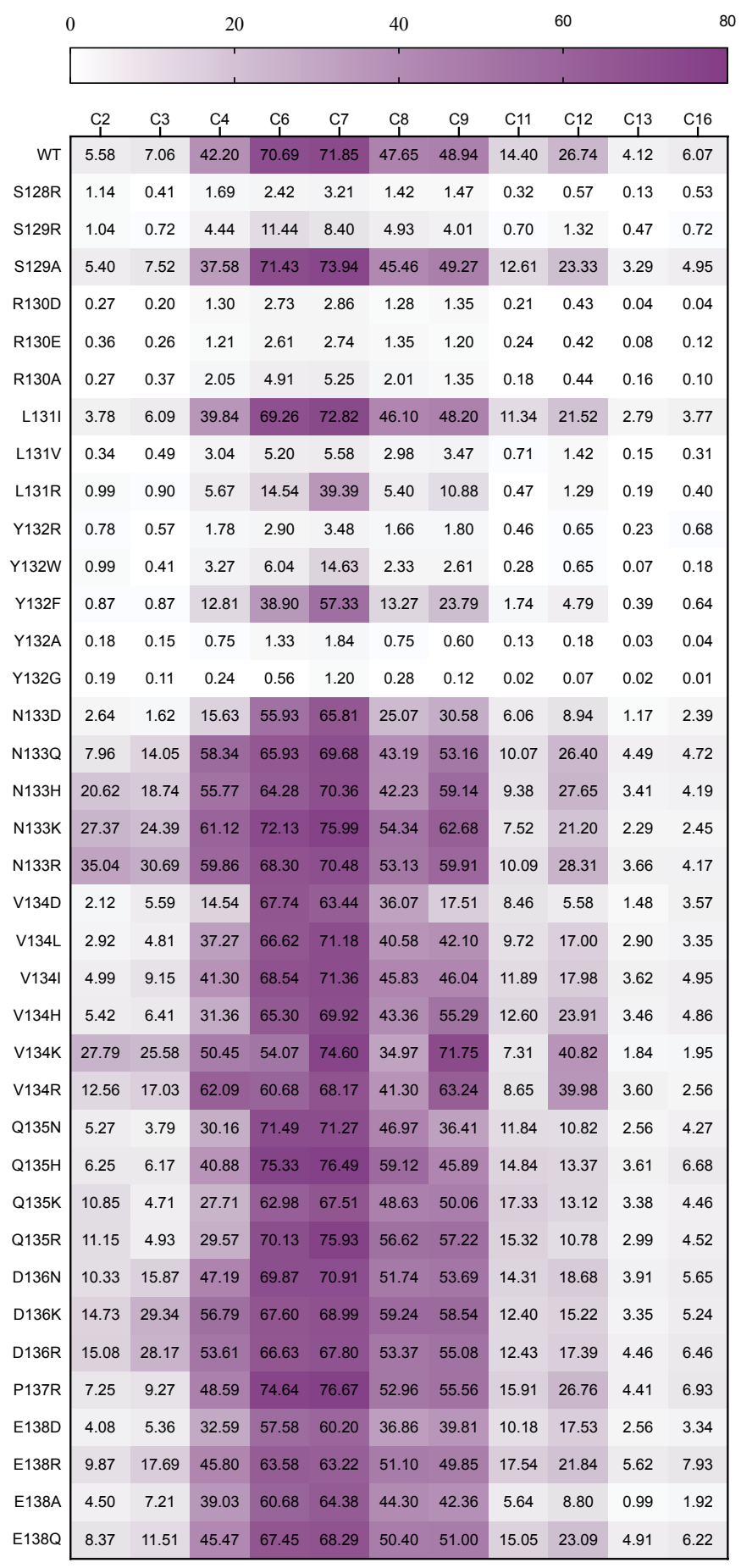

E Outside the pocket

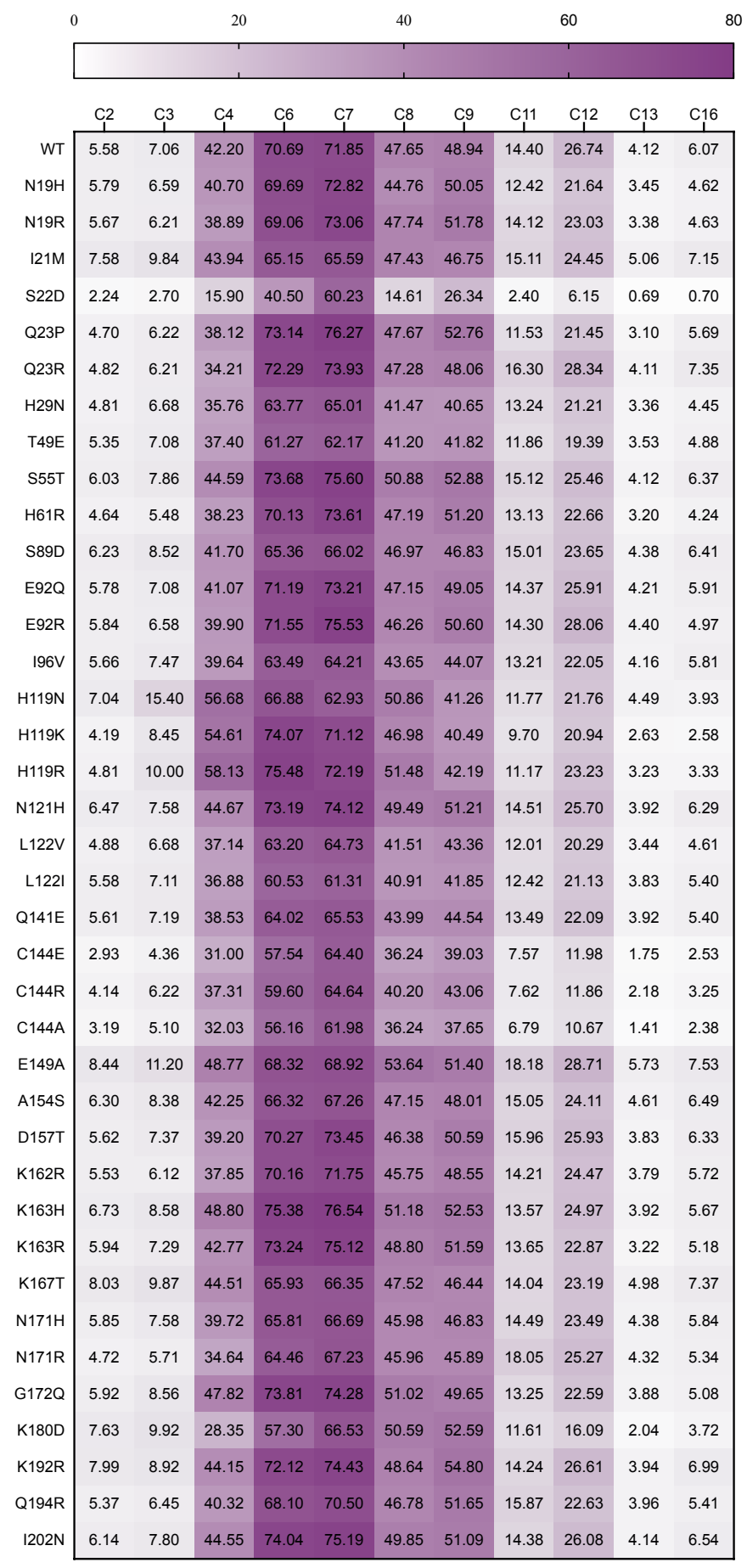

Figure S3. Screening and identification of beneficial amino acid substitutions in mA3CDA1 at the *DNMT3B* locus in HEK293T cells. (A–D) Editing efficiencies of mA3CDA1 variants harboring single amino acid substitutions in the four key loops (31, 20, 16, and 37 variants in loops 1, 3, 5, and 7, respectively). (E) Editing efficiencies of 38 mA3CDA1 variants harboring single amino acid substitutions outside the active-site pocket. Data represent mean values from two independent biological replicates.

Figure S4

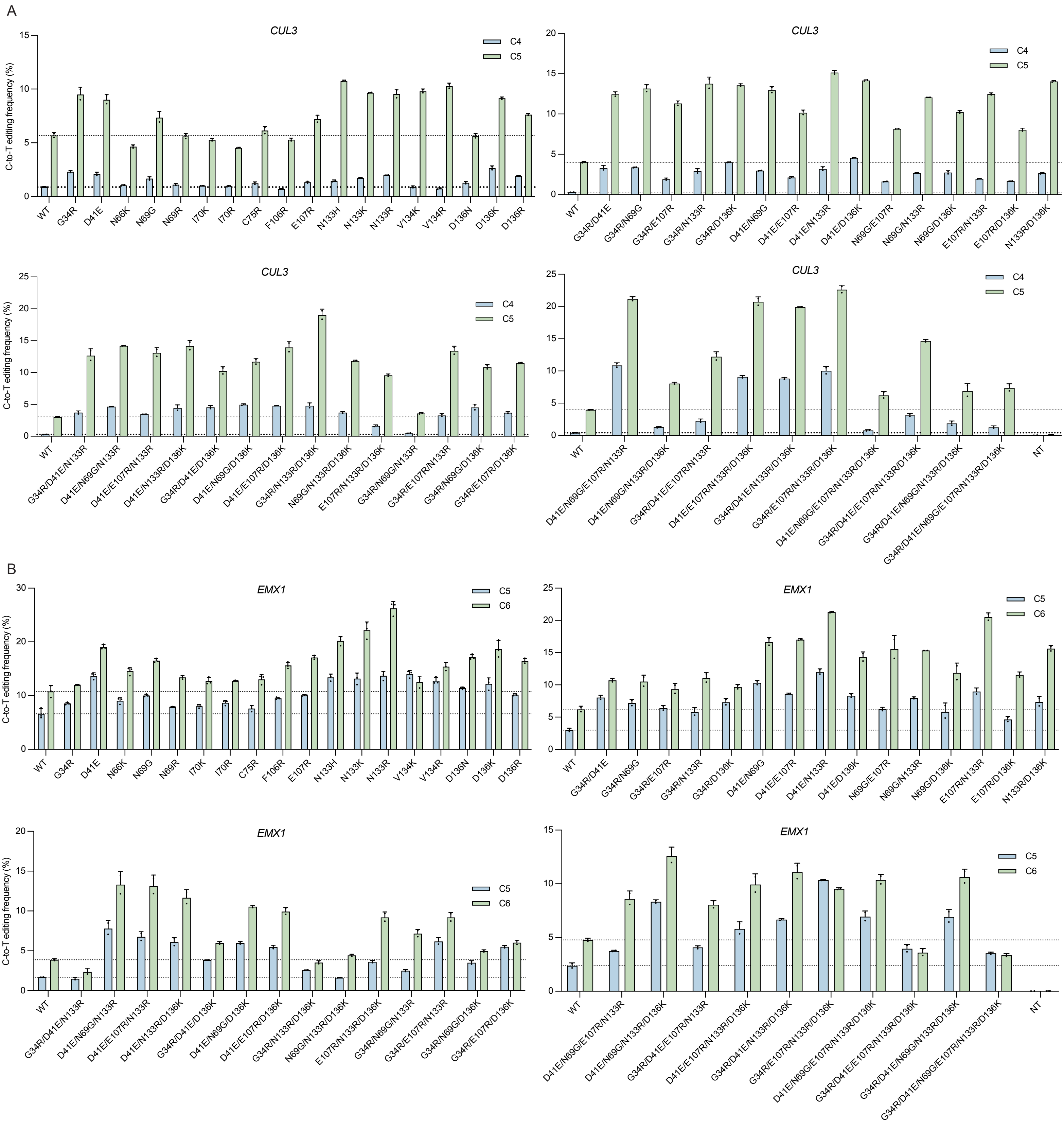

Figure S4. Base editing efficiencies of combinatorial mA3CDA1 mutants and the wild-type control at the *CUL3* and *EMX1* genomic loci in HEK293T cells. (A) C-to-T editing efficiencies of combinatorial mutants at the *CUL3* locus. (B) C-to-T editing efficiencies of combinatorial mutants at the *EMX1* locus. Data represent the mean ± SD from two independent biological replicates. Dashed lines indicate the editing efficiency of wild-type mA3CDA1.

Figure S5

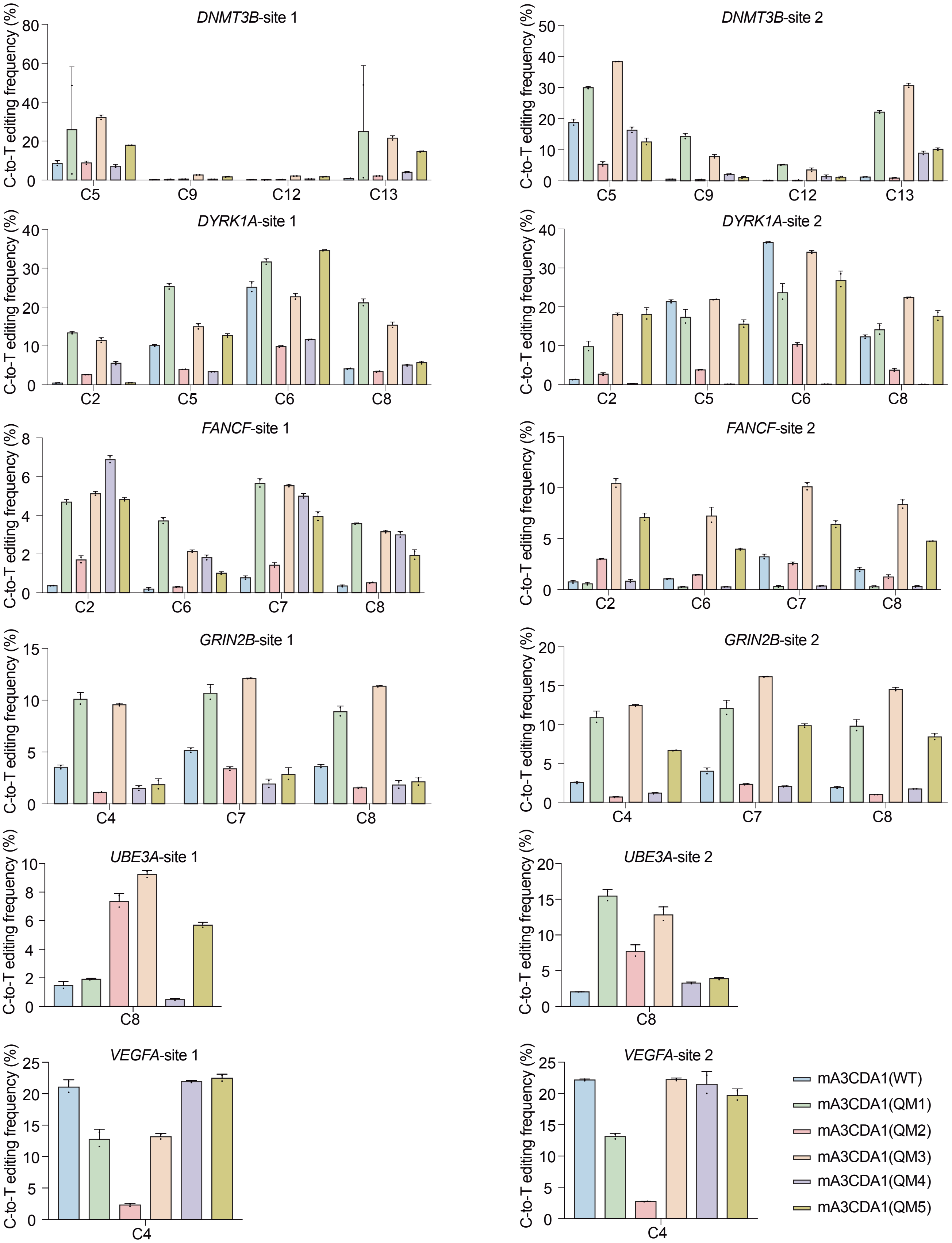

mA3CDA1(WT)	G34	D41	N69	E107	N133	D136
mA3CDA1(QM1)		D41E	N69G	E107R	N133R	
mA3CDA1(QM2)		D41E	N69G		N133R	D136K
mA3CDA1(QM3)		D41E		E107R	N133R	D136K
mA3CDA1(QM4)	G34R	D41E			N133R	D136K
mA3CDA1(QM5)	G34R			E107R	N133R	D136K

Figure S5. Compositions and editing efficiencies of combinatorial mA3CDA1 variants at non-NGG PAM loci in HEK293T cells. C-to-T editing efficiencies of mA3CDA1 mutants and mA3CDA1(WT) across 12 endogenous non-NGG PAM loci in HEK293T cells. Data represent mean ± SD from two independent biological replicates.

Figure S6

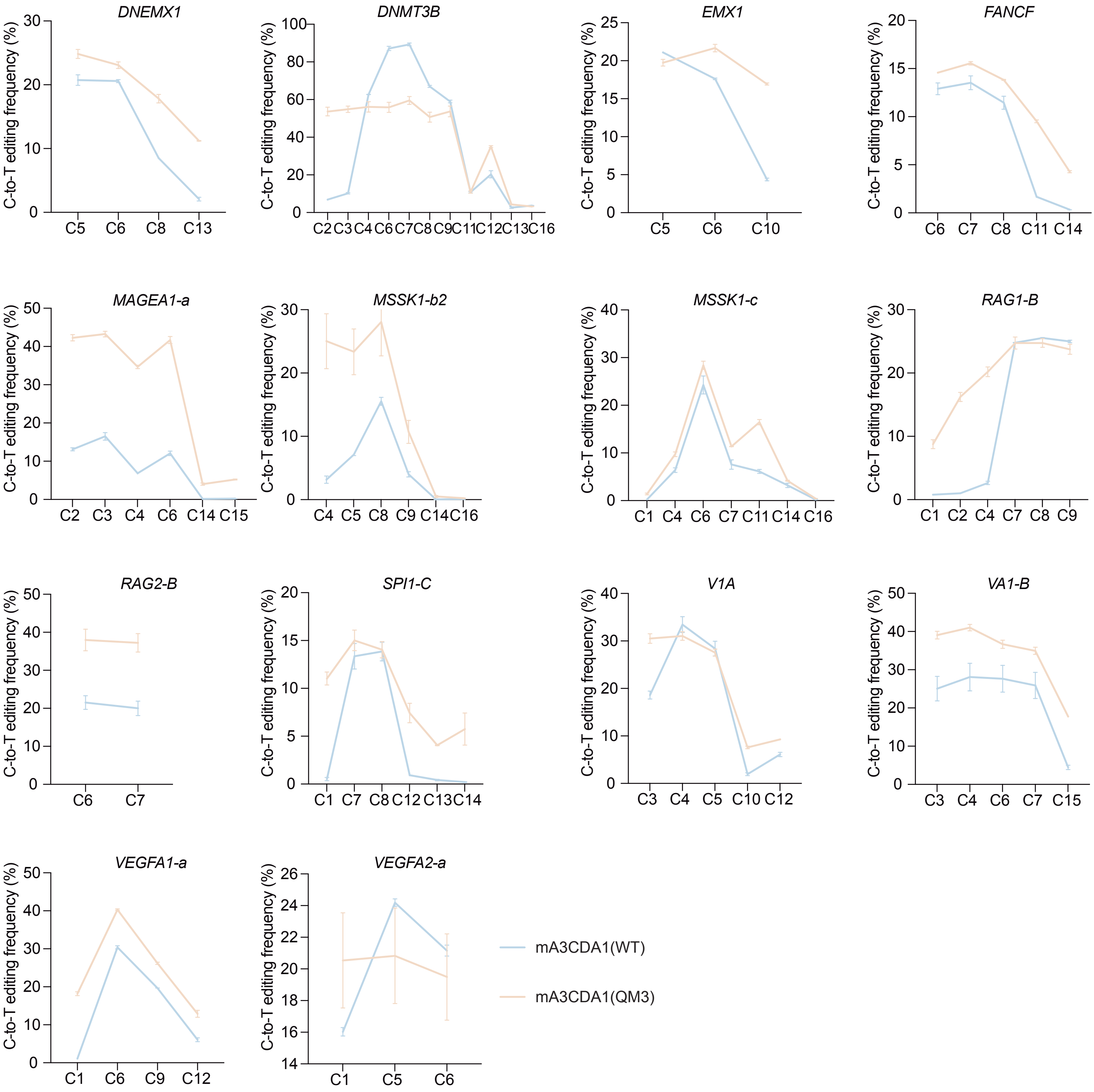

Figure S6. Base editing efficiencies of mA3CDA1(WT) and mA3CDA1(QM3) at 14 NGG PAM loci in HEK293T cells. Base editing efficiencies of mA3CDA1(WT) and mA3CDA1(QM3) at 14 individual endogenous genomic sites in HEK293T cells. Data represent mean ± SD from two independent biological replicates.

Figure S7

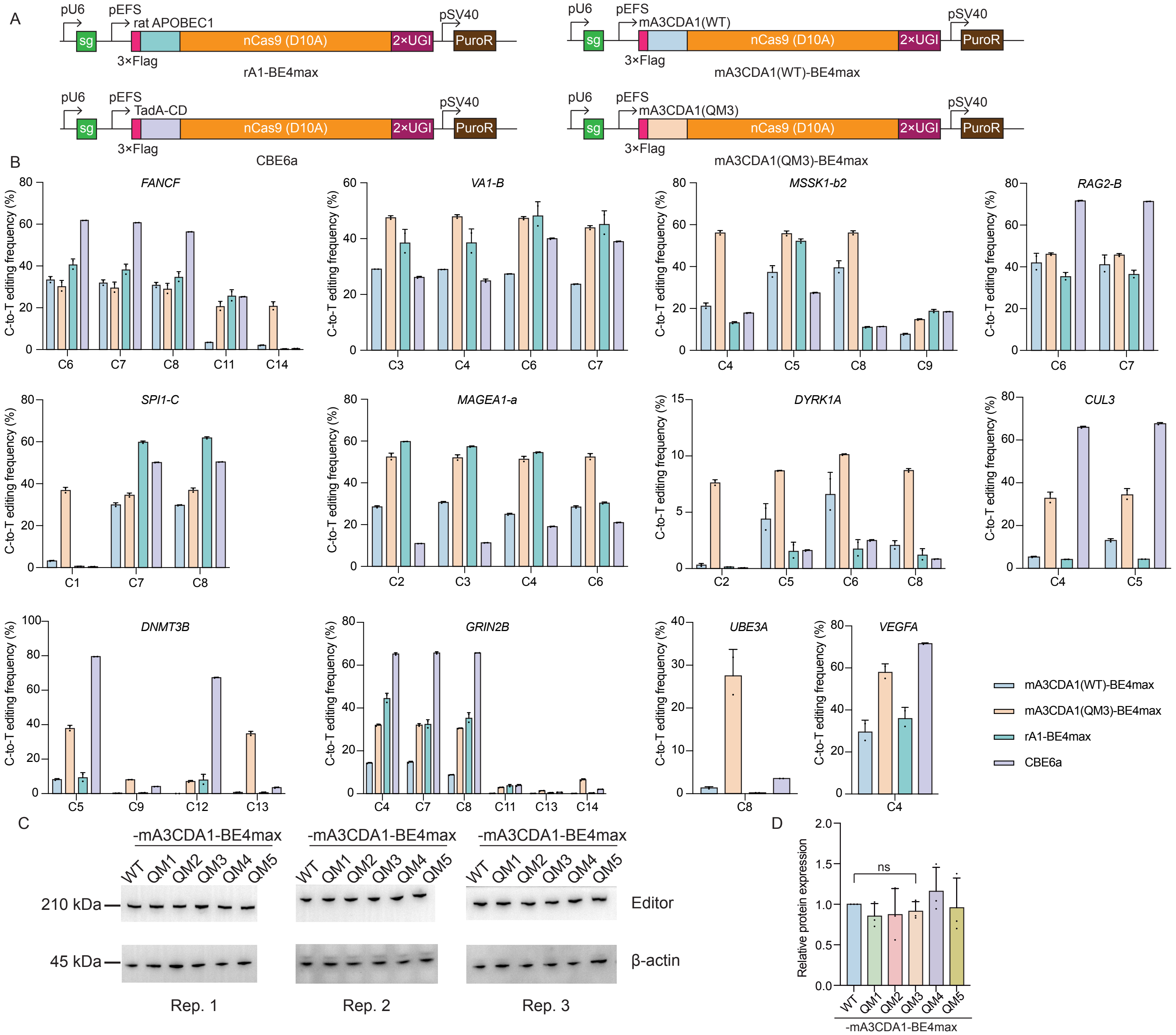

Figure S7. Base editing efficiency and protein expression levels of mA3CDA1(WT)-BE4max and mA3CDA1(QM3)-BE4max in HEK293T cells. (A) Schematic illustration of rA1-BE4max, CBE6a, mA3CDA1(WT)-BE4max, and mA3CDA1(QM3)-BE4max constructs. All editor fusion protein expression cassettes are driven by the EFS promoter, sgRNAs are driven by the U6 promoter, while the puromycin resistance gene (PuroR) is driven by a separate SV40 promoter. TadA-CD: TadA-derived cytidine deaminase. (B) Base editing frequencies of four editors at six NGG PAM sites (*FANCF*, *VA1-B, MSSK1-b2*, *RAG2-B*, *SPI1-C*, and *MAGEA1-a*) and six non-NGG PAM sites (*CUL3*, *DYRK1A*, *DNMT3B*, *GRIN2B*, *UBE3A*, and *VEGFA*) in HEK293T cells. Data represent mean ± SD from two independent biological replicates. (C) Protein expression levels of mA3CDA1(WT)-BE4max and the five quadruple mutants in HEK293T cells, as determined by Western blot. (D) Quantification of relative protein expression levels of mA3CDA1(WT)-BE4max and five quadruple mutants normalized to mA3CDA1(WT)-BE4max. Data represent mean ± SD from three independent biological replicates. Statistical significance was determined by one-way ANOVA Tukey's multiple comparisons (ns, not significant).

Figure S8

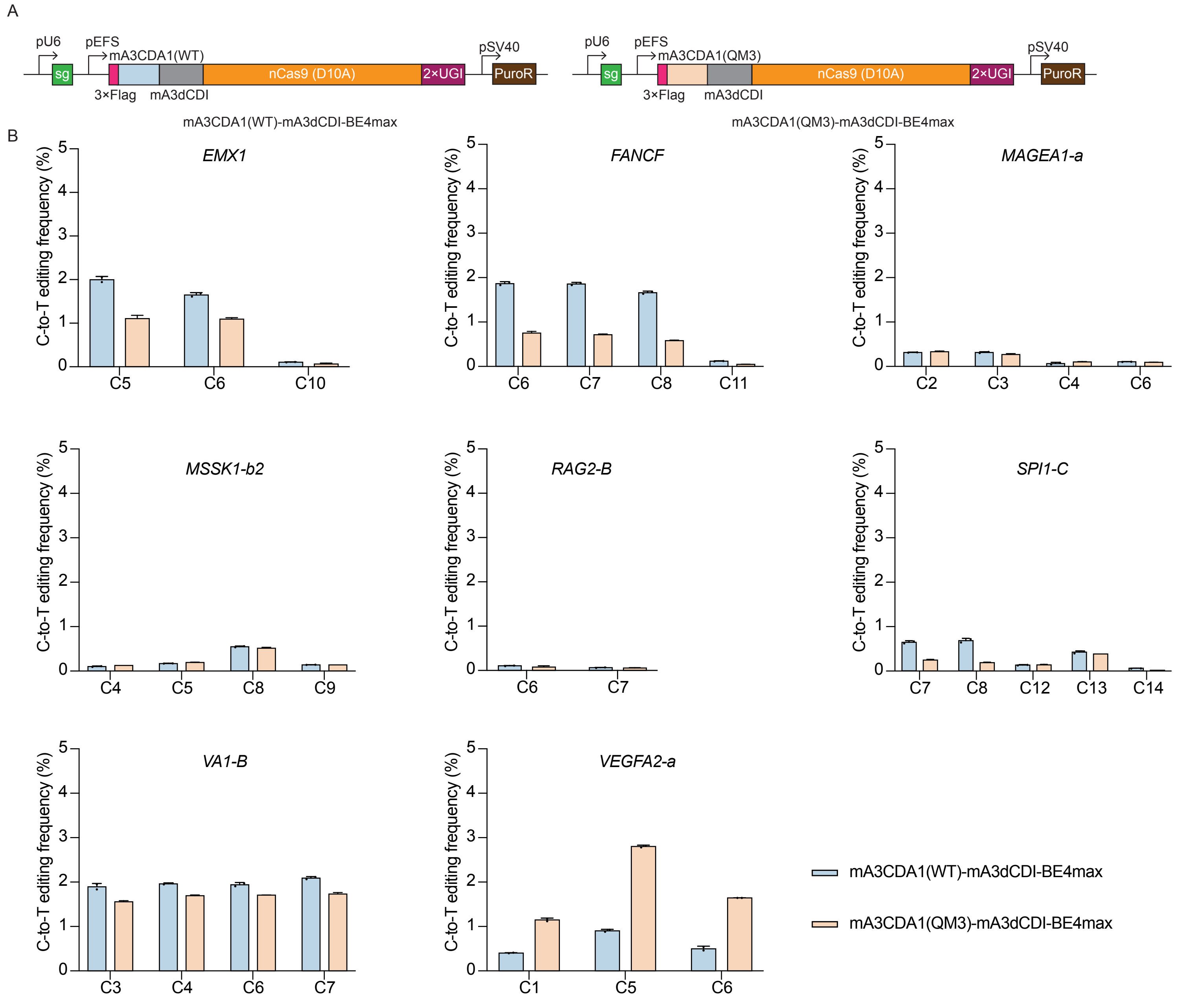

Figure S8. Base editing efficiencies of mA3CDA1(WT)-mA3dCDI-BE4max and mA3CDA1(QM3)-mA3dCDI-BE4max at eight endogenous NGG PAM genomic loci. (A) Schematic of the expression cassettes for mA3CDA1(WT)-mA3dCDI-BE4max and mA3CDA1(QM3)-mA3dCDI-BE4max constructs. (B) C-to-T base editing efficiencies of mA3CDA1(WT)-mA3dCDI-BE4max and mA3CDA1(QM3)-mA3dCDI-BE4max at eight endogenous NGG PAM genomic loci in HEK293T cells. Data represent mean ± SD from two independent biological replicates.

Figure S9

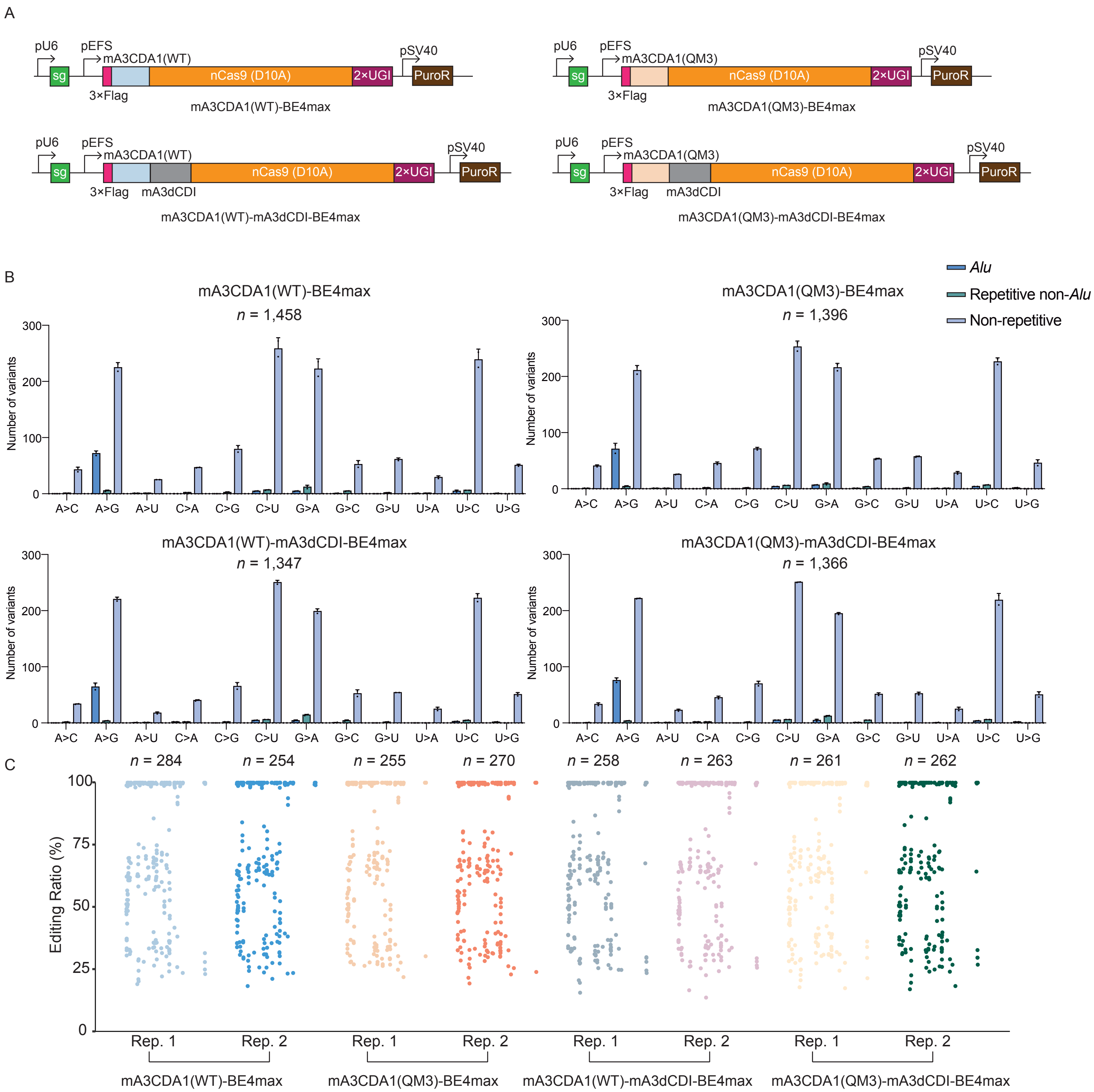

Figure S9. Transcriptomic off-target mutations induced by mA3CDA1(WT)-BE4max, mA3CDA1(QM3)-BE4max, mA3CDA1(WT)-mA3dCDI-BE4max, and mA3CDA1(QM3)-mA3dCDI-BE4max in HEK293T cells. (A) Schematic illustration of the mA3CDA1(WT)-BE4max, mA3CDA1(QM3)-BE4max, mA3CDA1(WT)-mA3dCDI-BE4max, and mA3CDA1(QM3)-mA3dCDI-BE4max constructs. All fusion protein expression cassettes are driven by the EFS promoter, sgRNAs are driven by the U6 promoter, while the puromycin resistance gene (PuroR) is expressed from a separate SV40 promoter. (B) Total RNA editing events detected by the RADAR pipeline for mA3CDA1(WT)-BE4max, mA3CDA1(QM3)-BE4max, mA3CDA1(WT)-mA3dCDI-BE4max, and mA3CDA1(QM3)-mA3dCDI-BE4max in HEK293T cells. The average number of variants per sample is indicated. (C) C-to-U RNA editing events detected by RADAR for the four groups in HEK293T cells. The number of C-to-U variants per replicate is indicated.

Figure S10

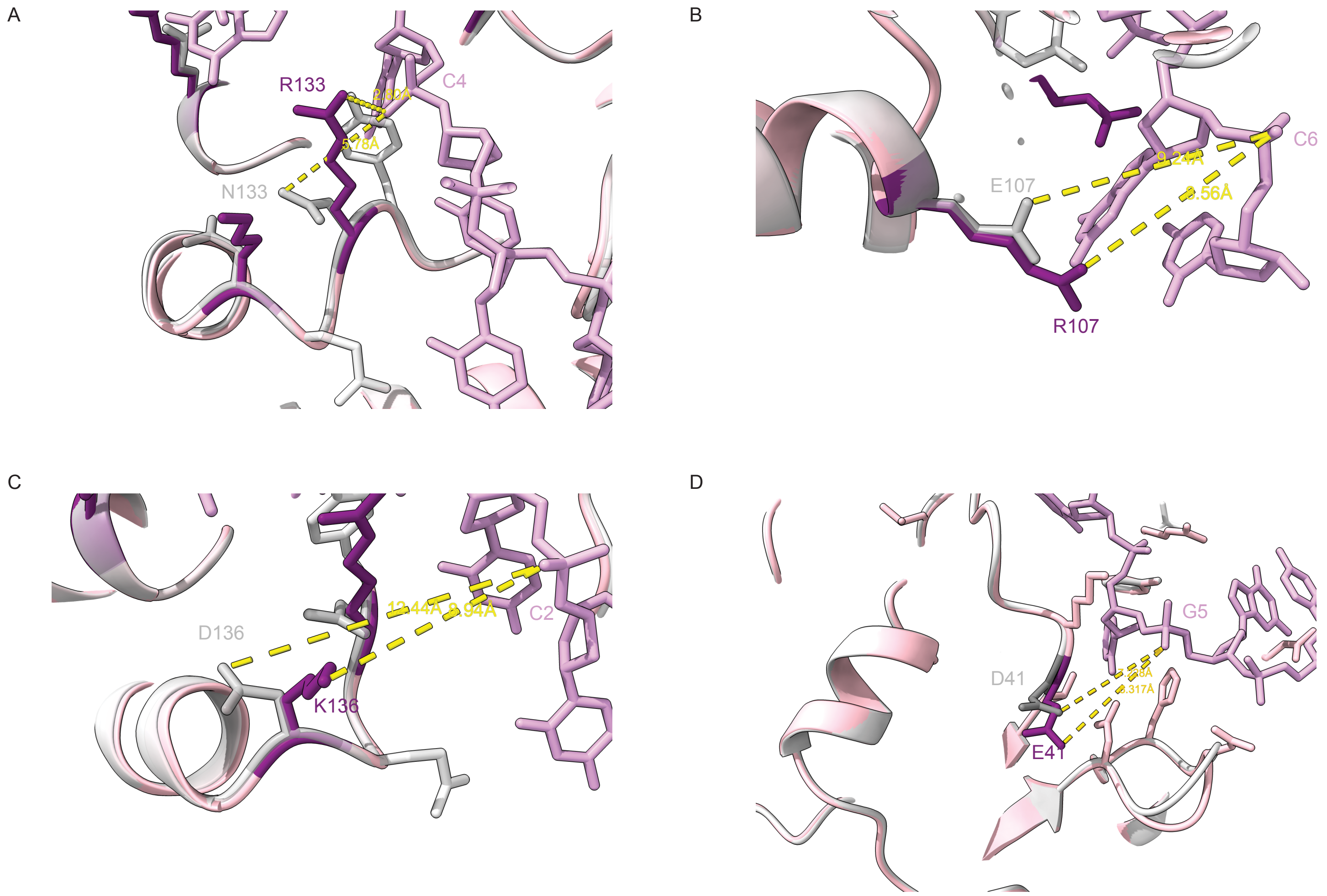

Figure S10. Potential structural basis of enhanced enzymatic activity in mA3CDA1(QM3). (A–D) Superimposed close-up views of the N133R (A), E107R (B), D136K (C), and D41E (D) substitutions. The mA3CDA1(WT) and mA3CDA1(QM3) structures are shown in light gray and pink, respectively. The target residues are shown in stick representation, with wild type residues colored in gray and mutated residues in purple. The DNA substrates are shown in plum. Dashed lines indicate the measured spatial distances (Å) between the specified residue side chains and the corresponding phosphate backbone atoms of the substrate DNA.

Figure S11

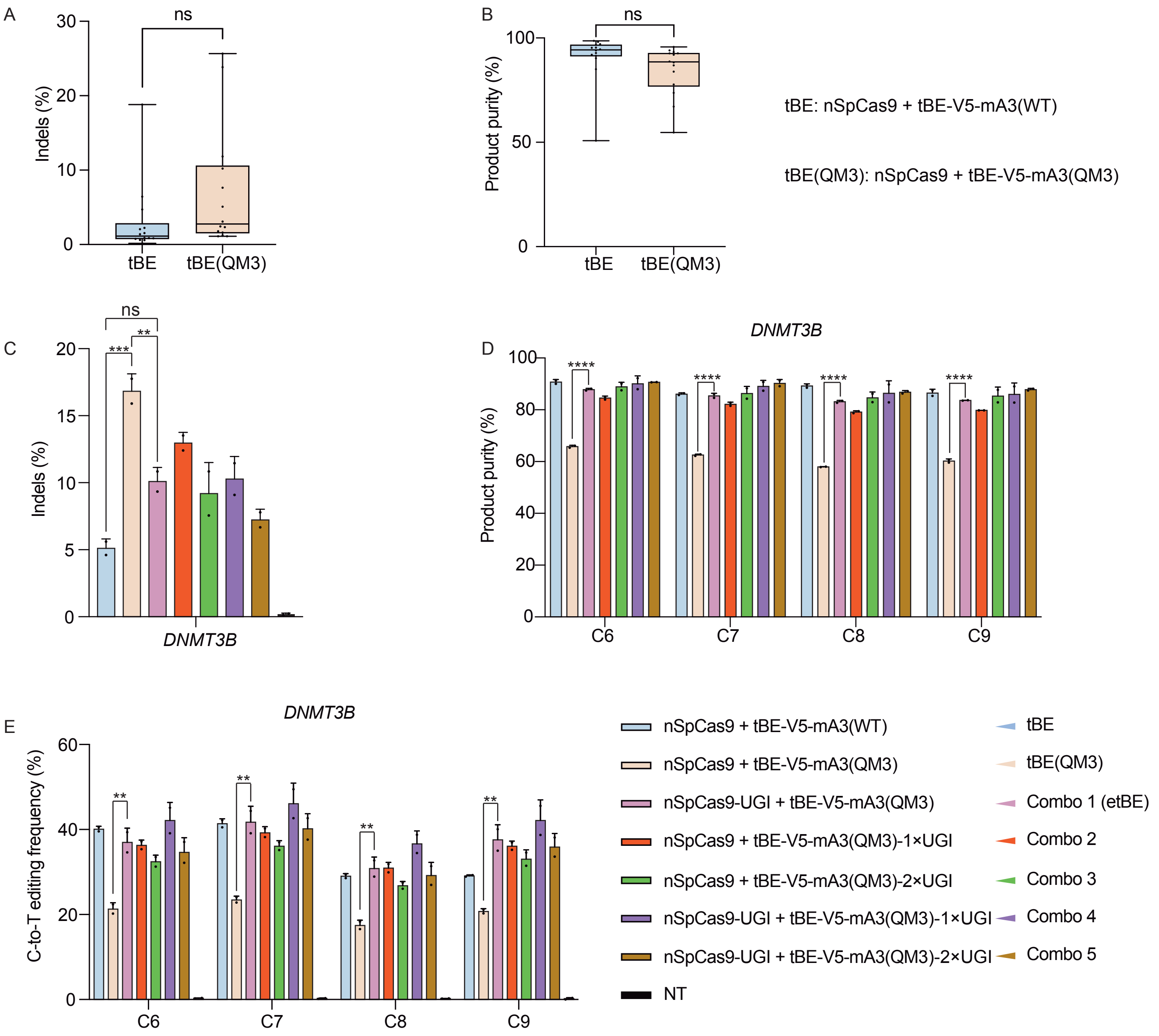

Figure S11. Editing performance of UGI fusion proteins at the *DNMT3B* locus. (A) Summary of indel frequencies for tBE and tBE(QM3) at the 14 NGG PAM genomic loci in HEK293T cell. (B) Summary of product purities for tBE and tBE(QM3) across the same loci. (C–E) Indel frequencies (C), product purities (D), and editing efficiencies (E) of variants incorporating one or two UGI domains fused to either nSpCas9 or the tBE-V5-mA3(QM3) architecture at the *DNMT3B* locus in HEK293T cells. Data represent mean ± SD from two independent biological replicates. Statistical significance in (A) and (B) was determined by unpaired two-tailed Student's *t*-test (ns, not significant). Statistical significance in (C–E) was determined by one-way ANOVA Tukey's multiple comparisons (ns, not significant; ***p* < 0.01; ****p* < 0.001; *****p* < 0.0001).

Figure S12

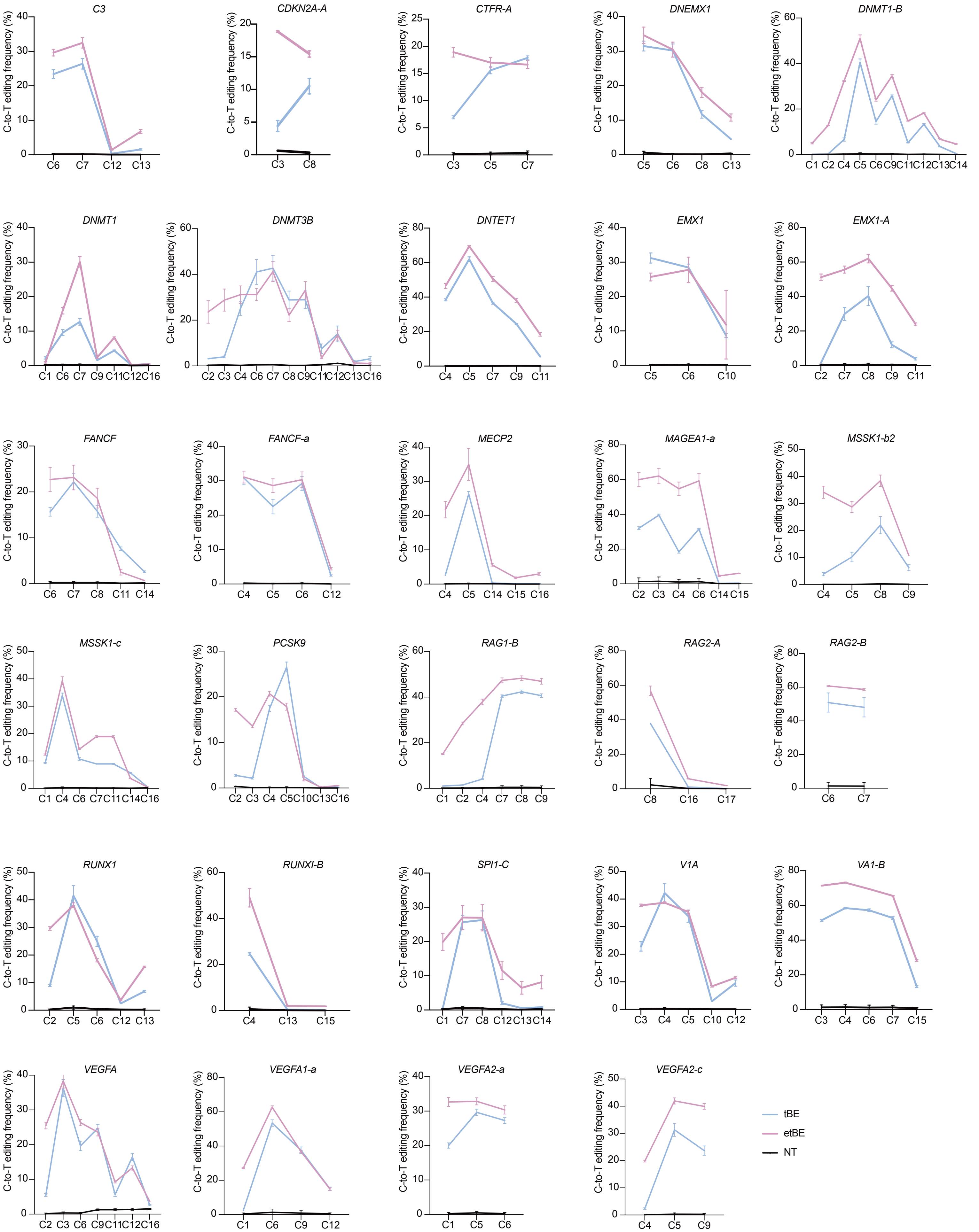

Figure S12. Editing efficiency of etBE at sgRNA loci across 29 NGG PAM genomic loci in HEK293T cells. Base editing efficiencies of tBE, etBE, and NT control groups at sgRNA loci across 29 individual endogenous NGG PAM loci. Data represent mean ± SD from three independent biological replicates.

Figure S13

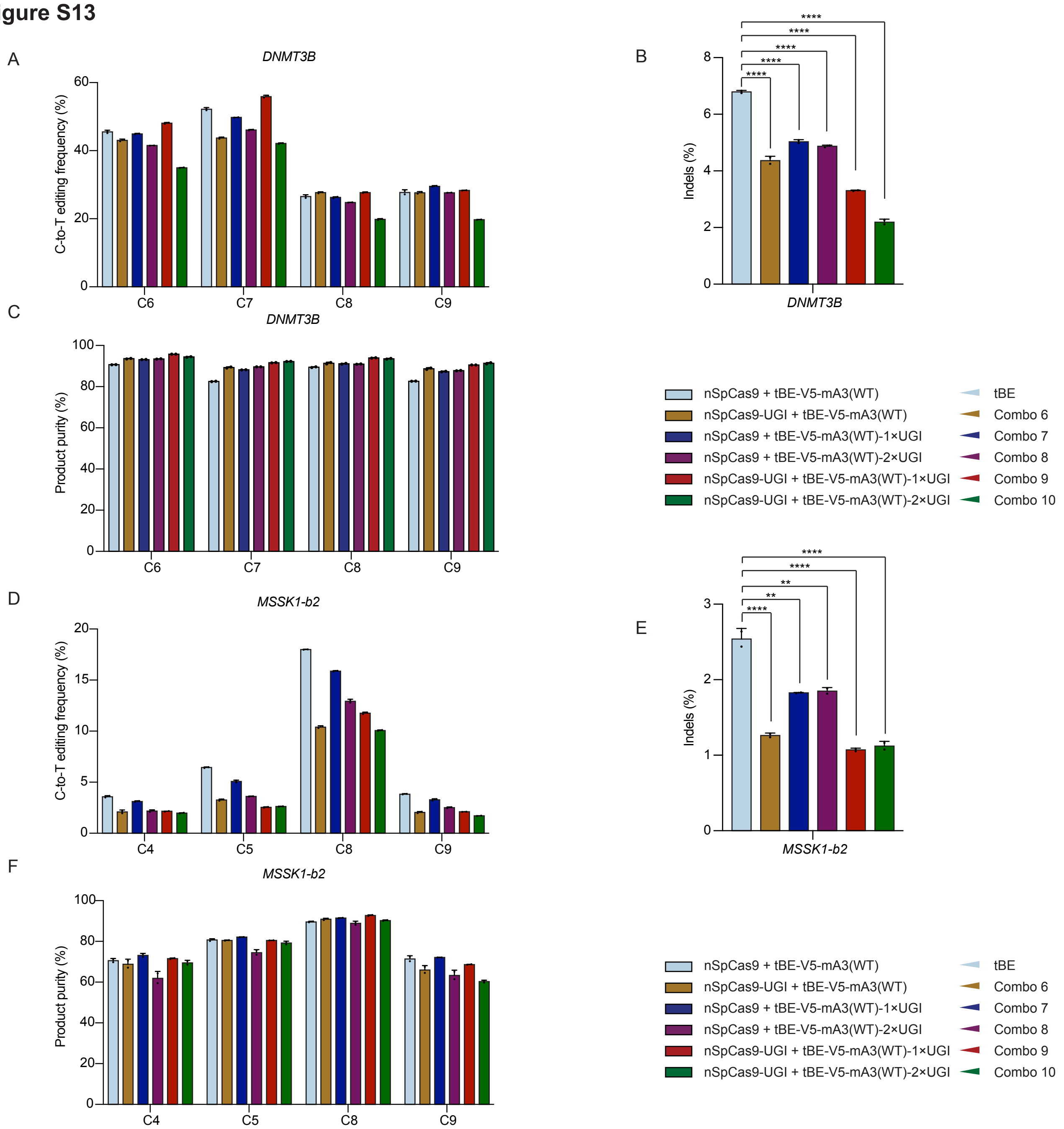

Figure S13. Editing performance of Combo 6–10 at the *DNMT3B* and *MSSK1-b2* loci in HEK293T cells. (A–C) C-to-T editing efficiency (A), indel frequency (B), and product purity (C) of Combo 6–10 at the *DNMT3B* locus. (D–F) C-to-T editing efficiency (D), indel frequency (E), and product purity (F) of Combo 6–10 at the *MSSK1-b2* locus. Data represent the mean ± SD from two independent biological replicates. Statistical significance was determined by one-way ANOVA Tukey's multiple comparisons (**$p < 0.01$, ****$p < 0.0001$).

Figure S14

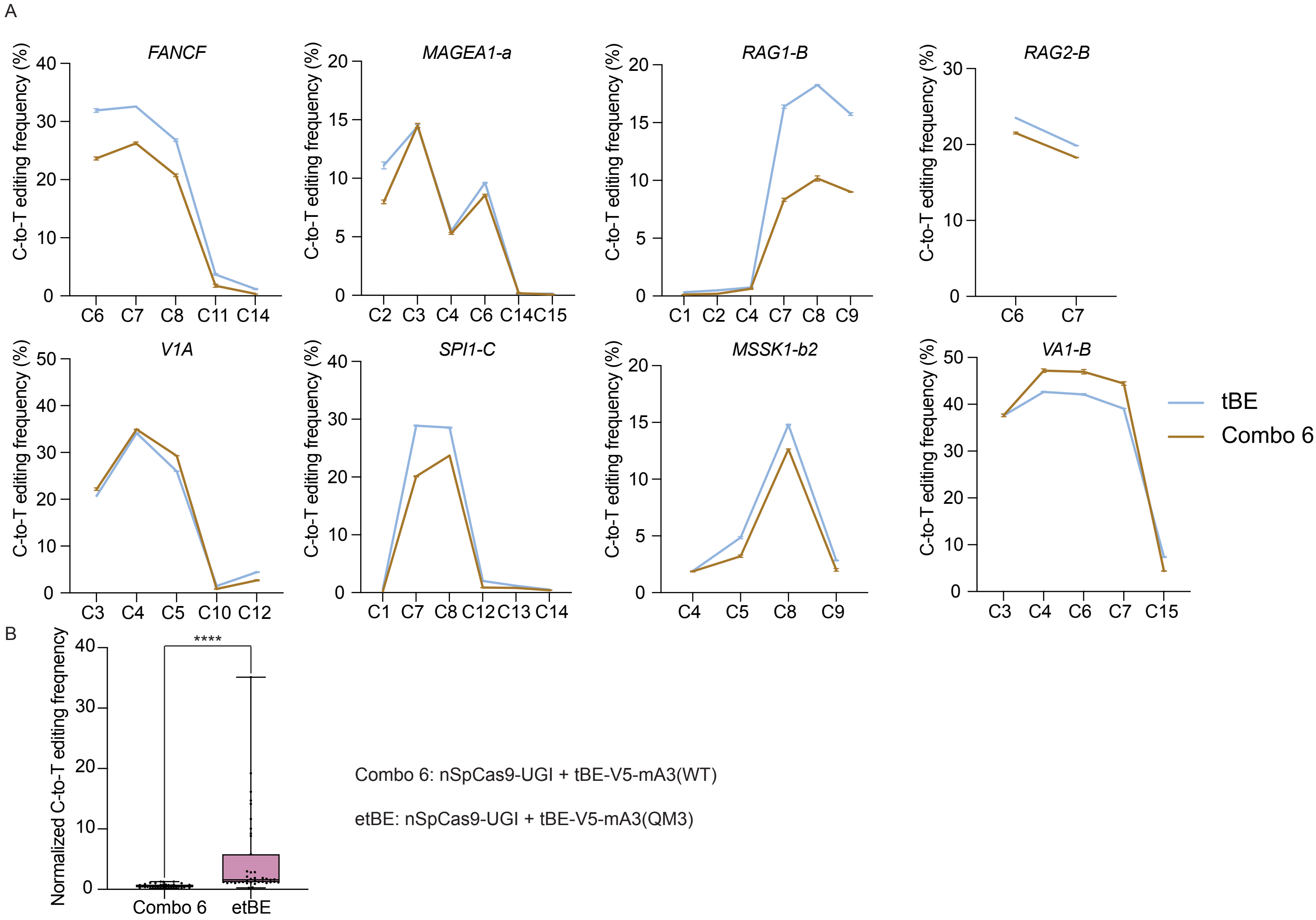

Figure S14. Base editing efficiency of Combo 6 at eight endogenous NGG PAM genomic loci in HEK293T cells. (A) Individual C-to-T editing efficiencies of tBE and Combo 6 at eight endogenous NGG PAM genomic loci. Data are shown as the mean ± SD from two independent biological replicates. (B) Normalized base editing efficiencies of Combo 6 and etBE at the same eight NGG PAM loci. Editing efficiencies of both Combo 6 and etBE were normalized to those of tBE tested in parallel. Statistical significance was determined using an unpaired two-tailed Student's *t*-test (*****p* < 0.0001).

Figure S15

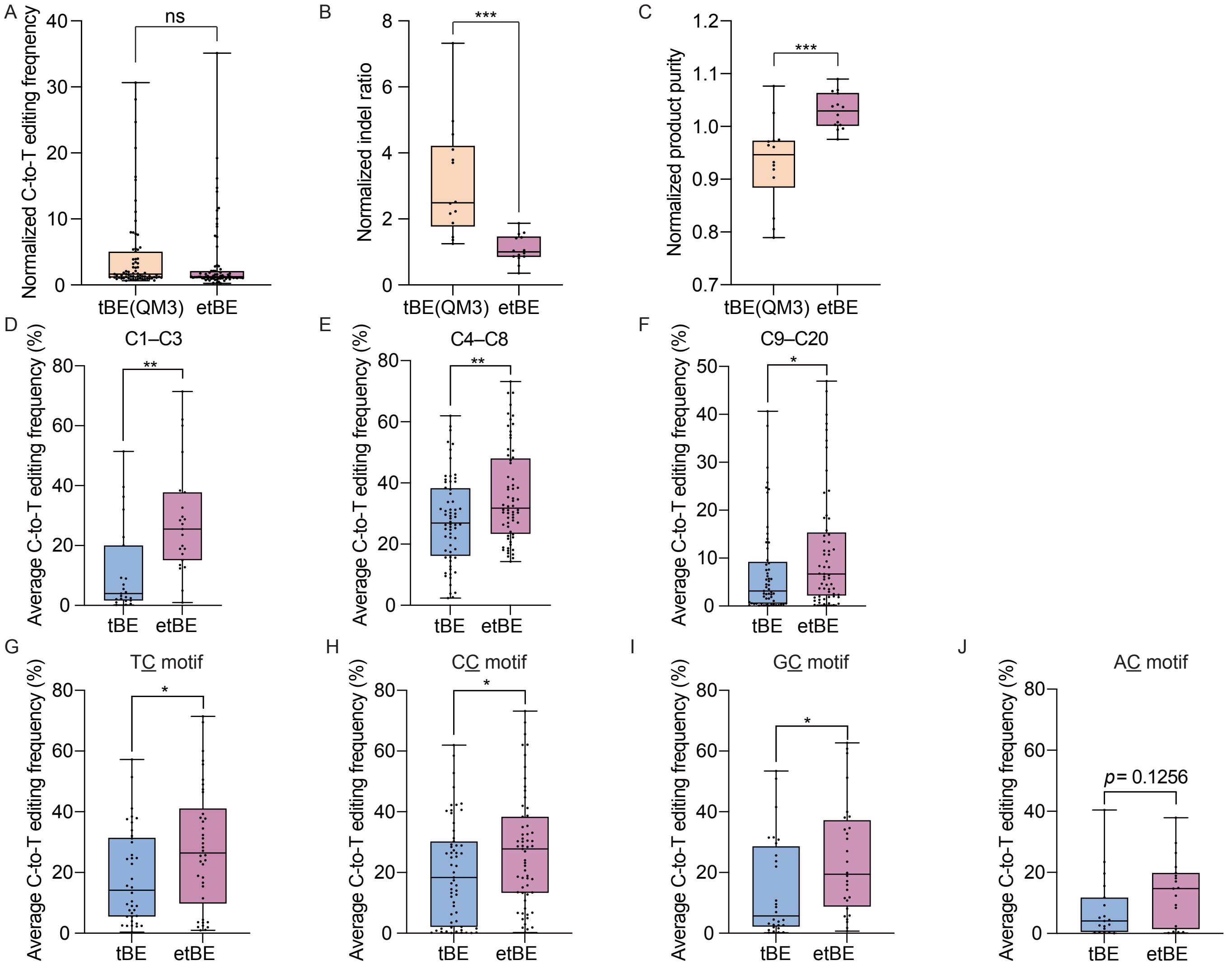

Figure S15. Characterization of etBE across NGG PAM loci in HEK293T cells. (A–C) Normalized base editing efficiencies (A), indel frequencies (B), and product purities (C) at 14 identical NGG PAM loci in HEK293T cells. tBE(QM3) and etBE were both normalized to tBE. Statistical significance was determined using an unpaired two-tailed Student's *t*-test. (ns, not significant; ***$p < 0.001$). (D–F) Average C-to-T editing efficiencies of tBE and etBE across PAM-distal (C1–C3; D), canonical (C4–C8; E), and PAM-proximal (C9–C20; F) protospacer positions. Statistical significance was determined by unpaired two-tailed Student's *t*-test (*$p < 0.05$; **$p < 0.01$). (G–J) Average C-to-T editing efficiencies of tBE and etBE at T<u>C</u> (G), C<u>C</u> (H), G<u>C</u> (I), and A<u>C</u> (J) dinucleotide motifs. Statistical significance was determined by unpaired two-tailed Student's *t*-test (*$p < 0.05$).

Figure S16

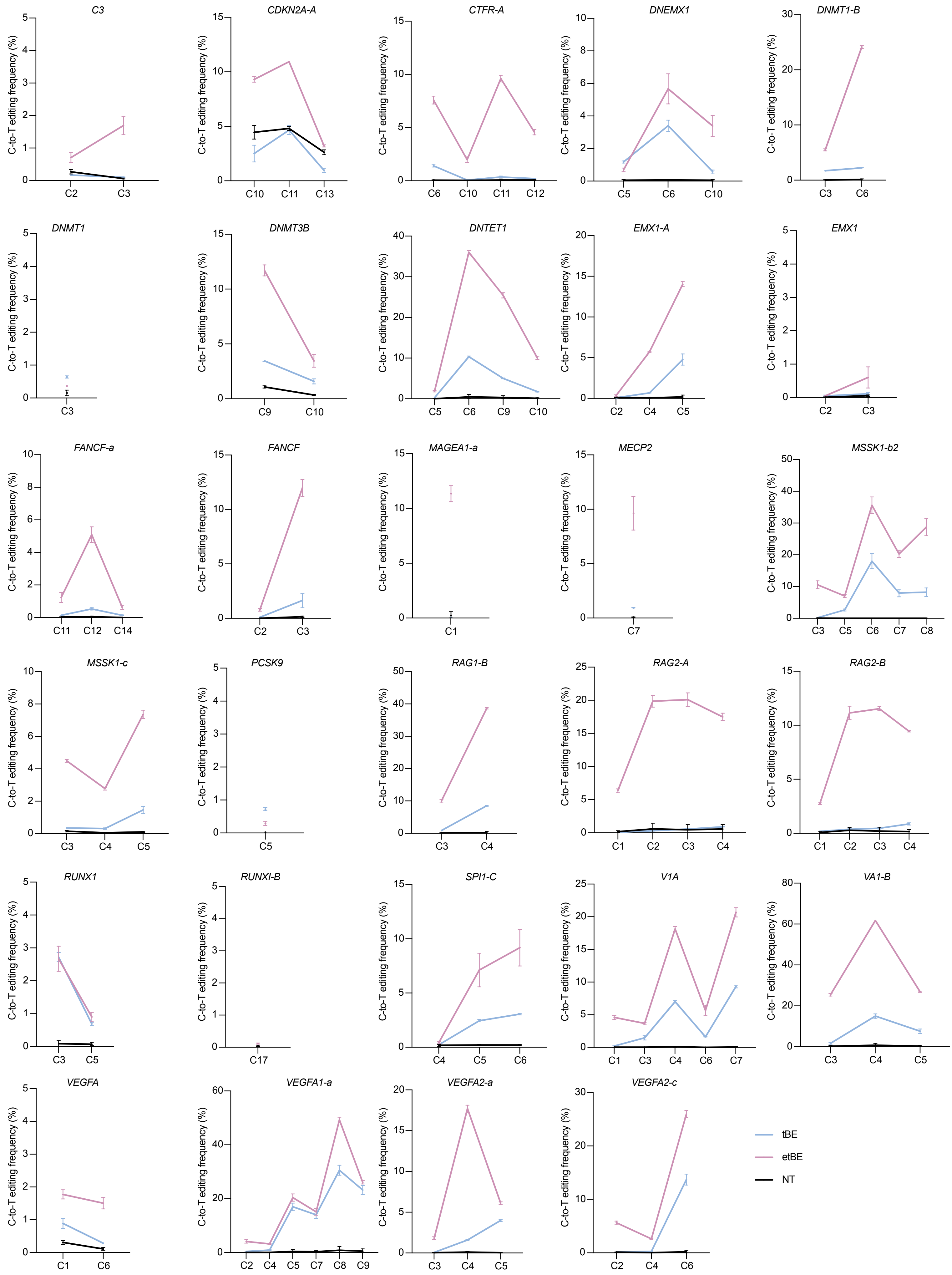

Figure S16. Base editing efficiencies of tBE and etBE at hsgRNA loci across 29 endogenous NGG PAM genomic sites in HEK293T cells. Base editing efficiencies of tBE, etBE, and NT control groups at hsgRNA loci for 29 individual endogenous NGG PAM sites. Data represent the mean ± SD from three independent biological replicates.

Figure S17

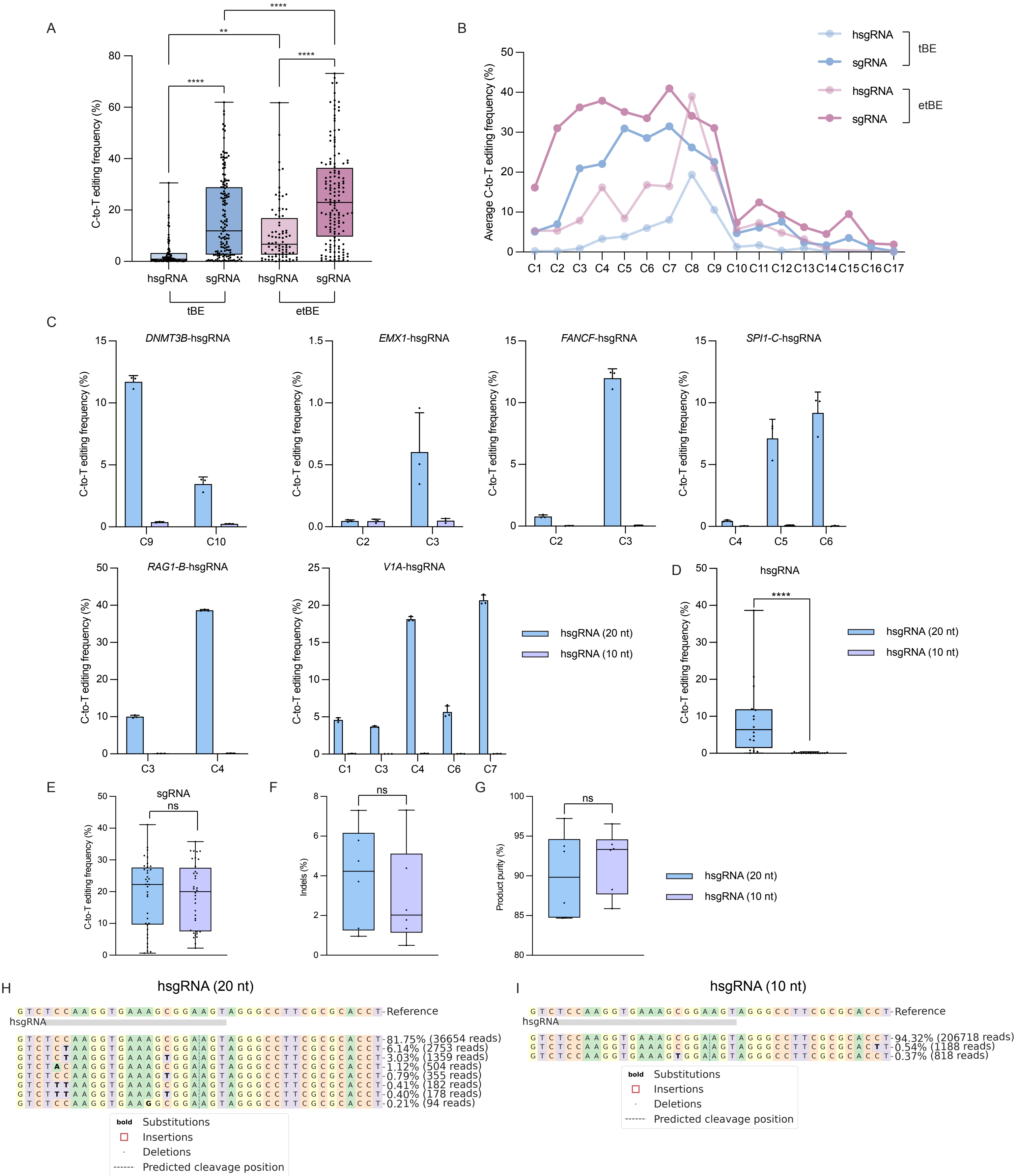

Figure S17. Base editing efficiencies of etBE at hsgRNA loci. (A) Average C-to-T editing efficiencies of tBE and etBE at hsgRNA and sgRNA loci across 29 endogenous NGG PAM genomic sites in HEK293T cells. Statistical significance was determined by one-way ANOVA with Tukey's multiple comparisons test (**$p < 0.01$; ****$p < 0.0001$). (B) Average C-to-T editing efficiencies of tBE and etBE across all cytosine positions at the hsgRNA and sgRNA loci. (C) Editing efficiencies of etBE at hsgRNA sites using 20-nt or 10-nt hsgRNA spacer lengths at six individual genomic loci. Data represent the mean ± SD from three independent biological replicates. (D) Summary of base editing efficiencies shown in panel C. Statistical significance was determined by unpaired two-tailed Student's *t*-test (****$p < 0.0001$). (E) C-to-T editing frequencies of etBE at sgRNA loci when using 20-nt or 10-nt hsgRNA spacer lengths at six genomic targets. (F) Indel frequencies of etBE using 20-nt or 10-nt hsgRNA spacer lengths across six genomic sites. (G) Product purity of etBE using 20-nt or 10-nt hsgRNA spacer lengths across six genomic sites. (H and I) Allele frequency tables of etBE using 20-nt (H) or 10-nt (I) hsgRNA spacer lengths at the *FANCF* locus. Statistical significance in panels E–G was determined by unpaired two-tailed Student's *t*-test (ns, not significant).

Figure S18

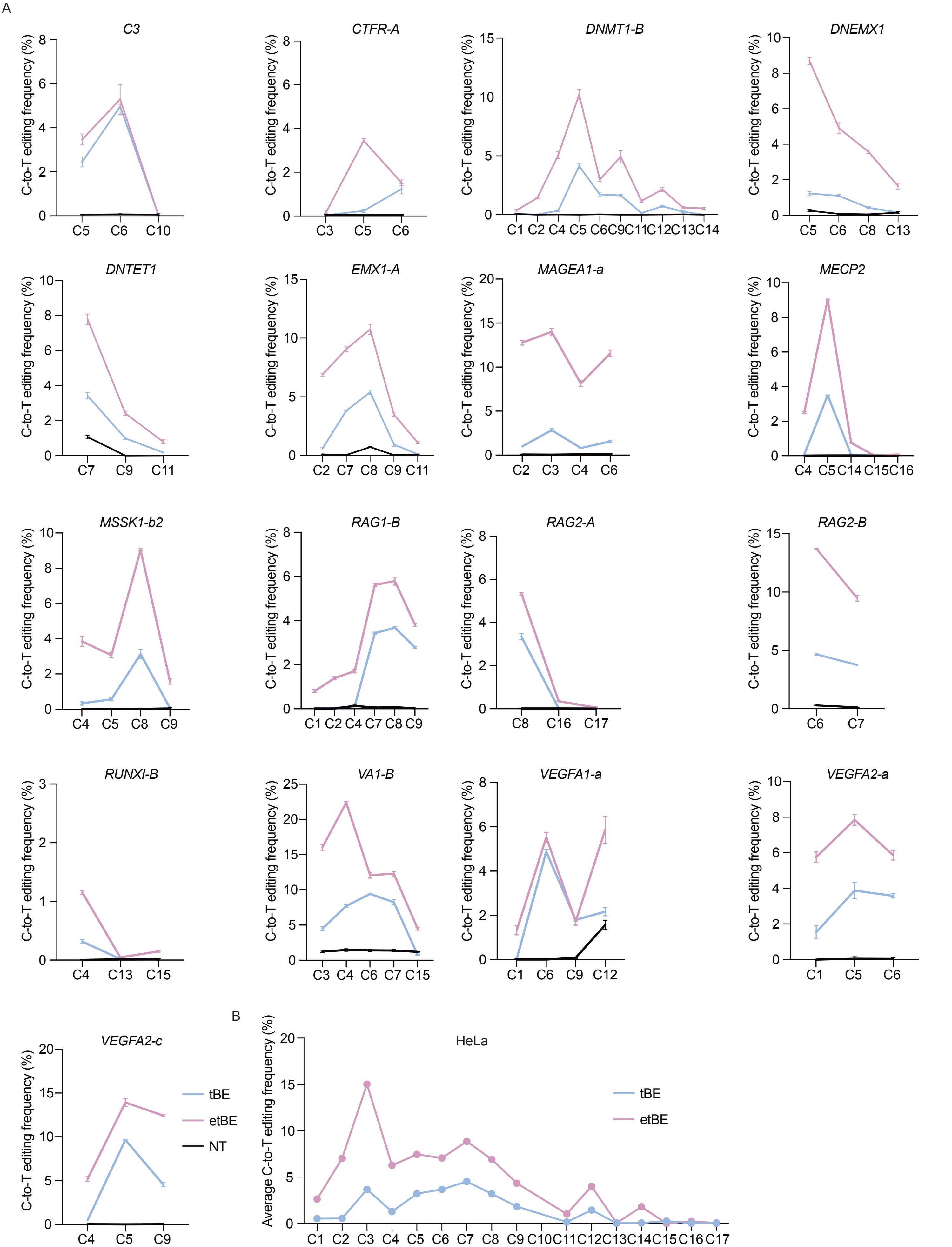

Figure S18. Editing efficiency of etBE in HeLa cells. (A) Editing frequencies of tBE, etBE, and NT control at 17 NGG PAM genomic loci in HeLa cells. Data are presented as mean ± SD from three independent biological replicates. (B) Average C-to-T editing frequency of tBE and etBE at each protospacer position. Data are shown as mean values.

Figure S19

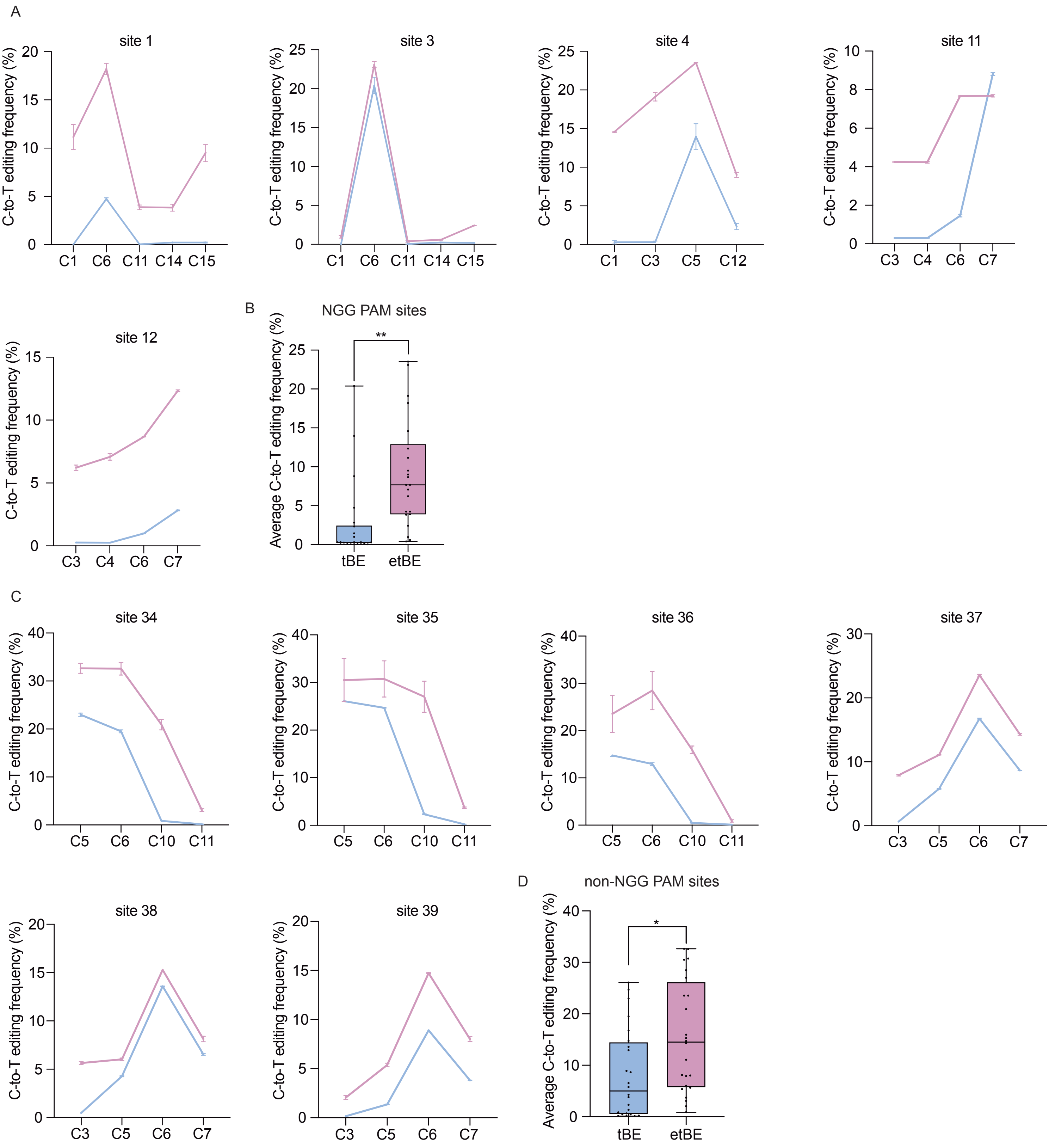

Figure S19. Editing efficiency of etBE in N2a cells. (A) C-to-T editing efficiencies of tBE and etBE at five individual NGG PAM endogenous loci. (B) Summary of average C-to-T editing efficiencies of tBE and etBE across the same five NGG PAM loci. (C) C-to-T editing efficiencies of tBE and etBE at six individual non-NGG PAM endogenous loci. (D) Summary of average C-to-T editing efficiencies of tBE and etBE across the same six non-NGG PAM loci. Data represent mean ± SD from two independent biological replicates. Statistical significance was determined by unpaired two-tailed Student's *t*-test (*$p < 0.05$; **$p < 0.01$).

Figure S20

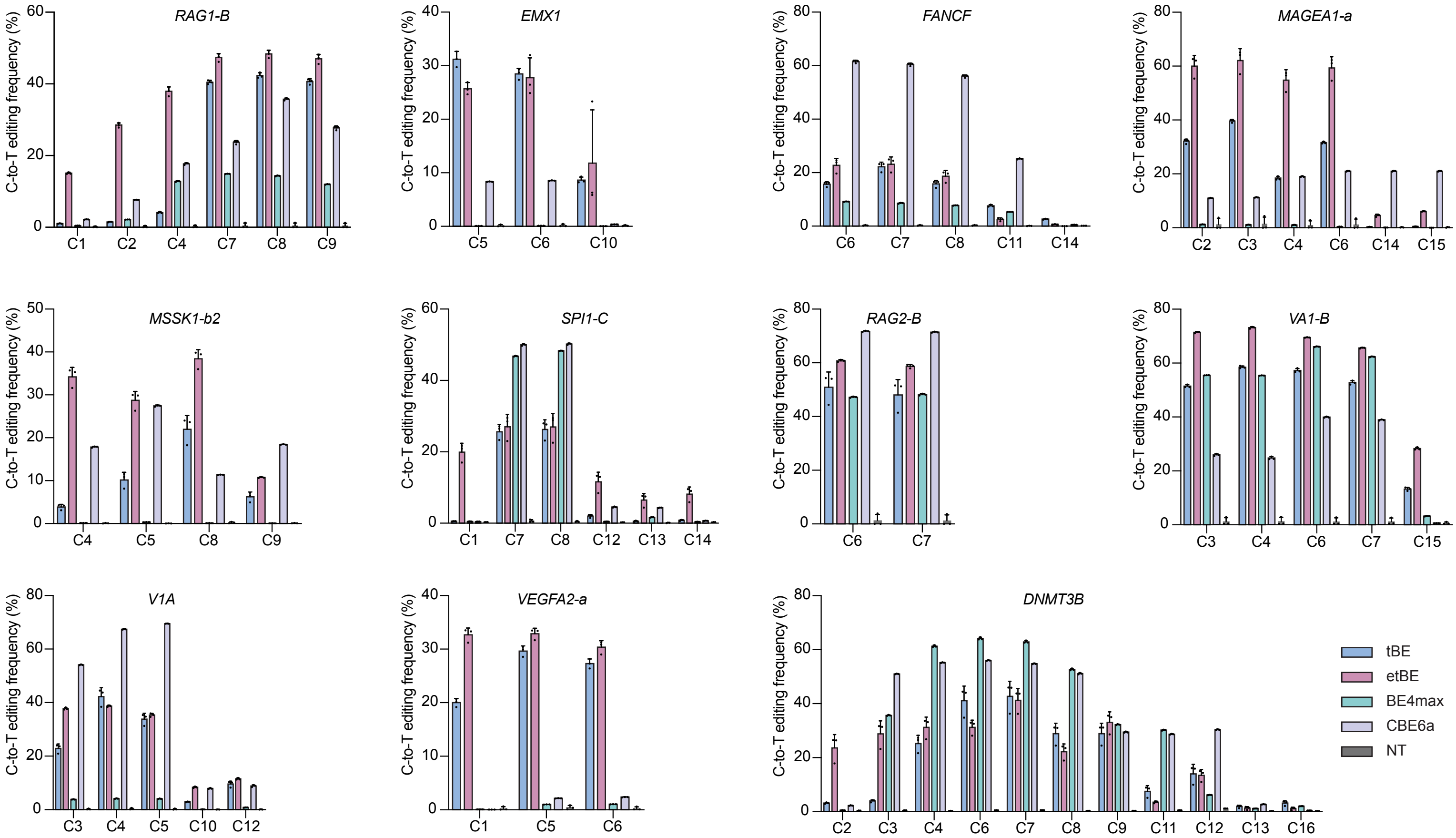

Figure S20. Base editing efficiencies of tBE, etBE, BE4max, CBE6a, and NT control at 11 endogenous NGG PAM loci in HEK293T cells. C-to-T editing efficiencies of tBE, etBE, BE4max, CBE6a, and NT control at 11 endogenous NGG PAM loci. Data represent the mean ± SD from three independent biological replicates.

Figure S21

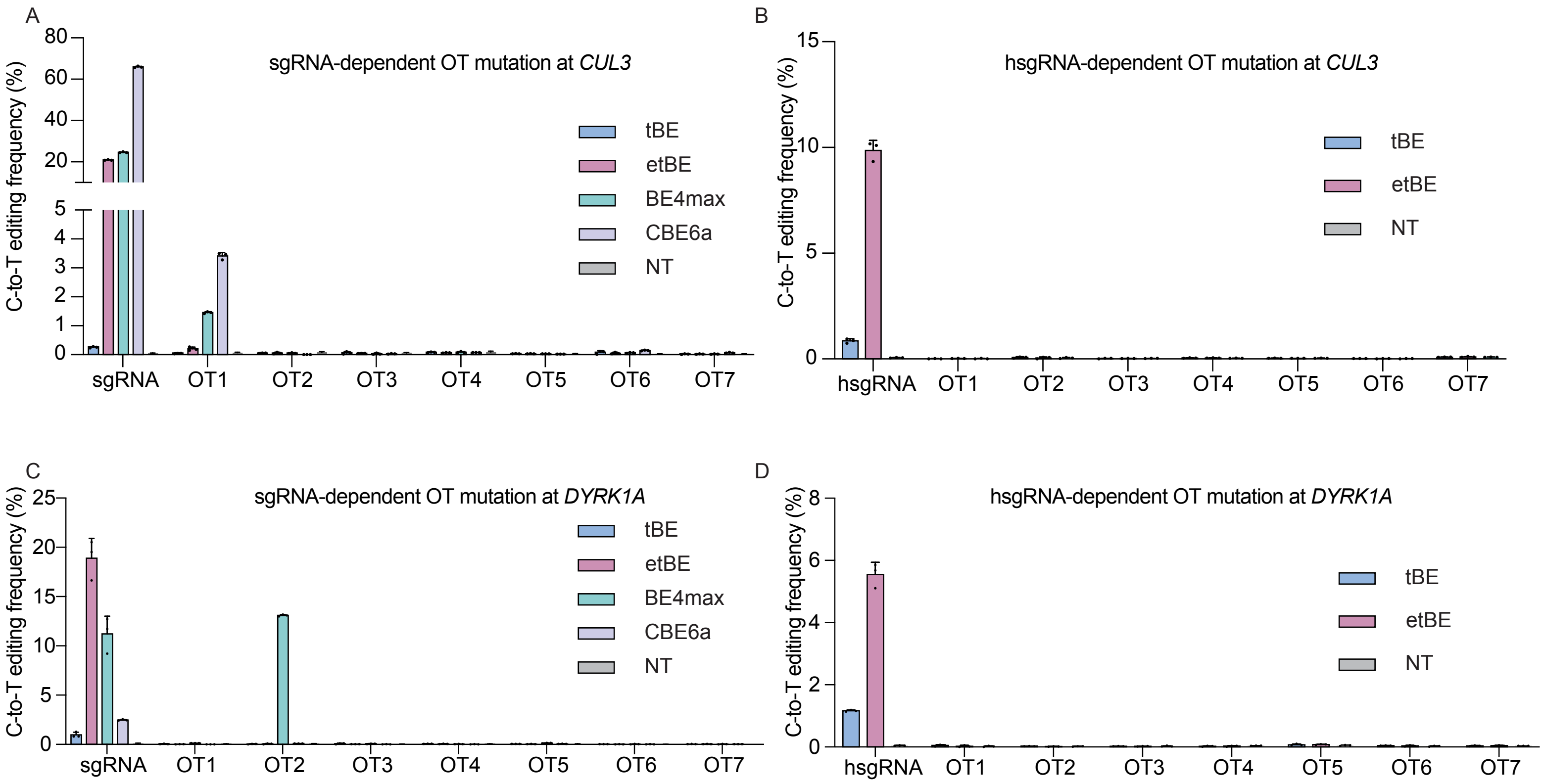

Figure S21. sgRNA- and hsgRNA-dependent OT mutations induced by etBE at non-NGG PAM loci in HEK293T cells. (A and C) sgRNA-dependent OT mutation frequencies induced by tBE, etBE, BE4max, CBE6a, and NT control groups at the *CUL3* (A) and *DYRK1A* (C) endogenous loci harboring non-NGG PAMs. (B and D) hsgRNA-dependent OT mutation frequencies induced by tBE, etBE, and NT groups at the *CUL3* (B) and *DYRK1A* (D) endogenous loci. Data represent the mean ± SD from three independent biological replicates.

Figure S22

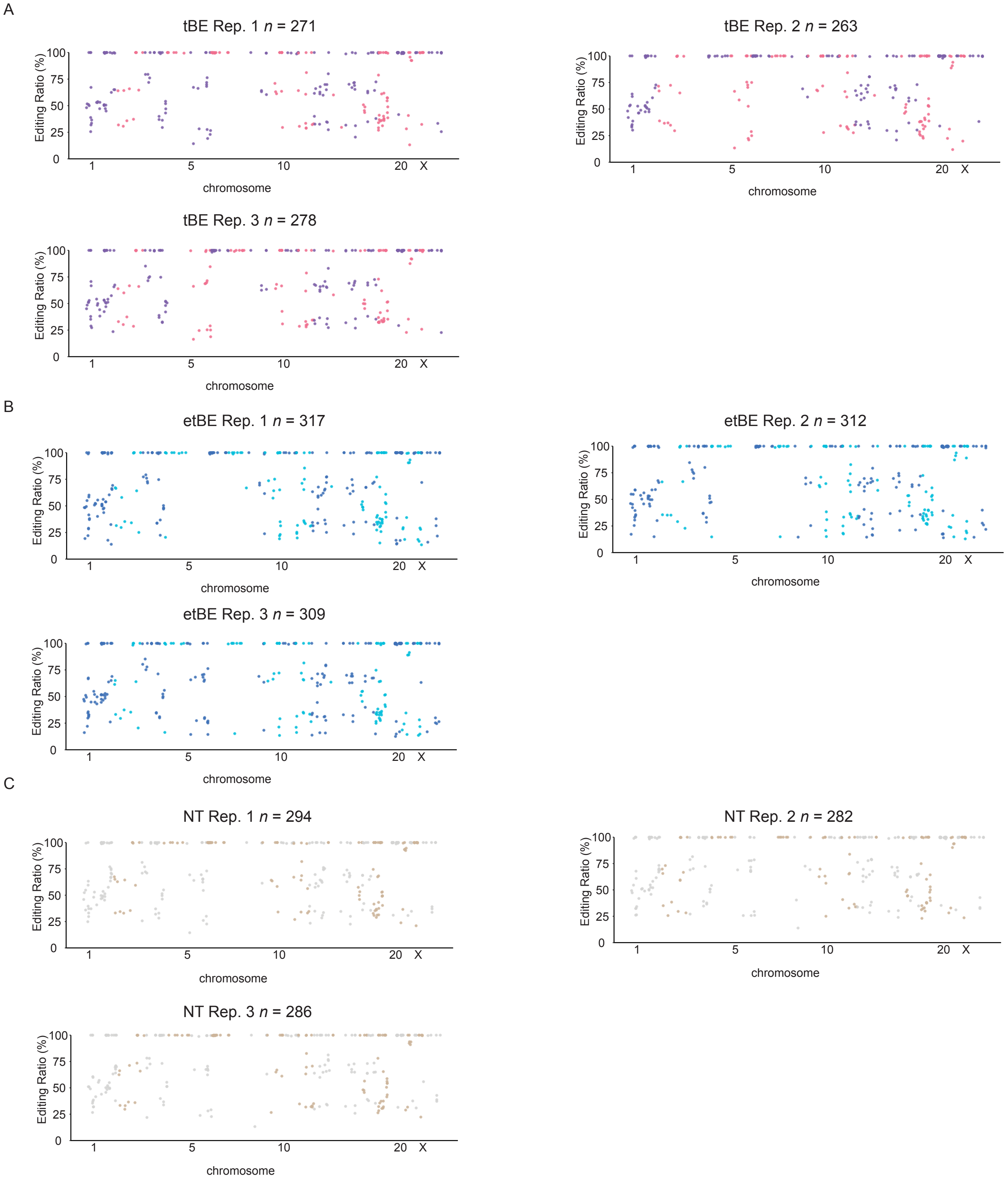

Figure S22. Transcriptomic off-target mutations of etBE. (A–C) Editing ratios and chromosomal distribution of C-to-U RNA variants detected in tBE (A), etBE (B), and NT control (C) groups targeting the *MAGEA1-a* endogenous locus in HEK293T cells. Data are presented as Manhattan plots, with each variant represented as a dot. The number of variants is indicated above each plot.

Figure S23

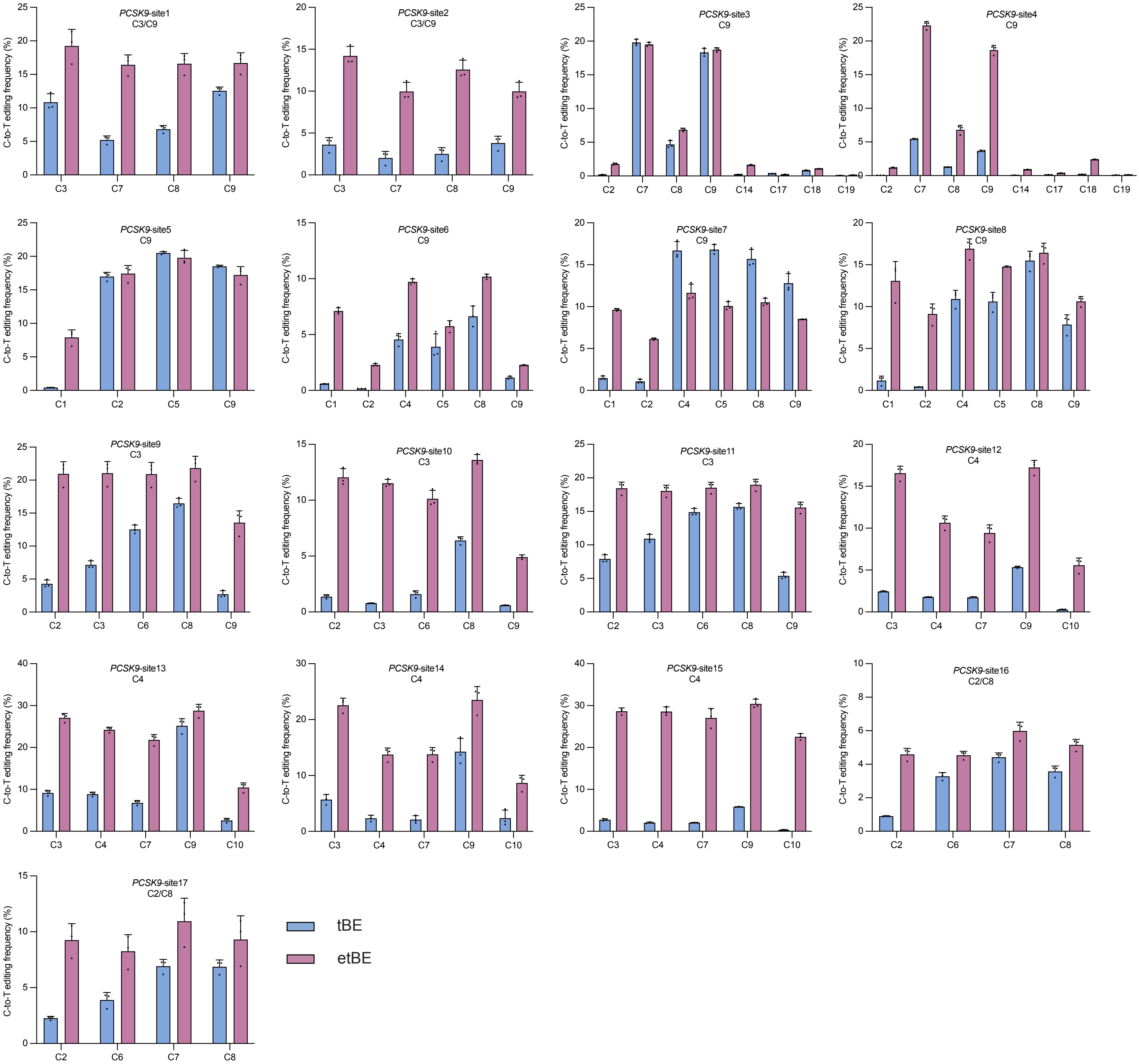

Figure S23. Base editing efficiencies of tBE and etBE using 17 different hsgRNA-sgRNA pairs targeting *PCSK9*. C-to-T editing efficiencies of tBE and etBE with 17 hsgRNA-sgRNA pairs targeting *PCSK9* in HEK293T cells. Data represent the mean ± SD from three independent biological replicates.

Figure S24

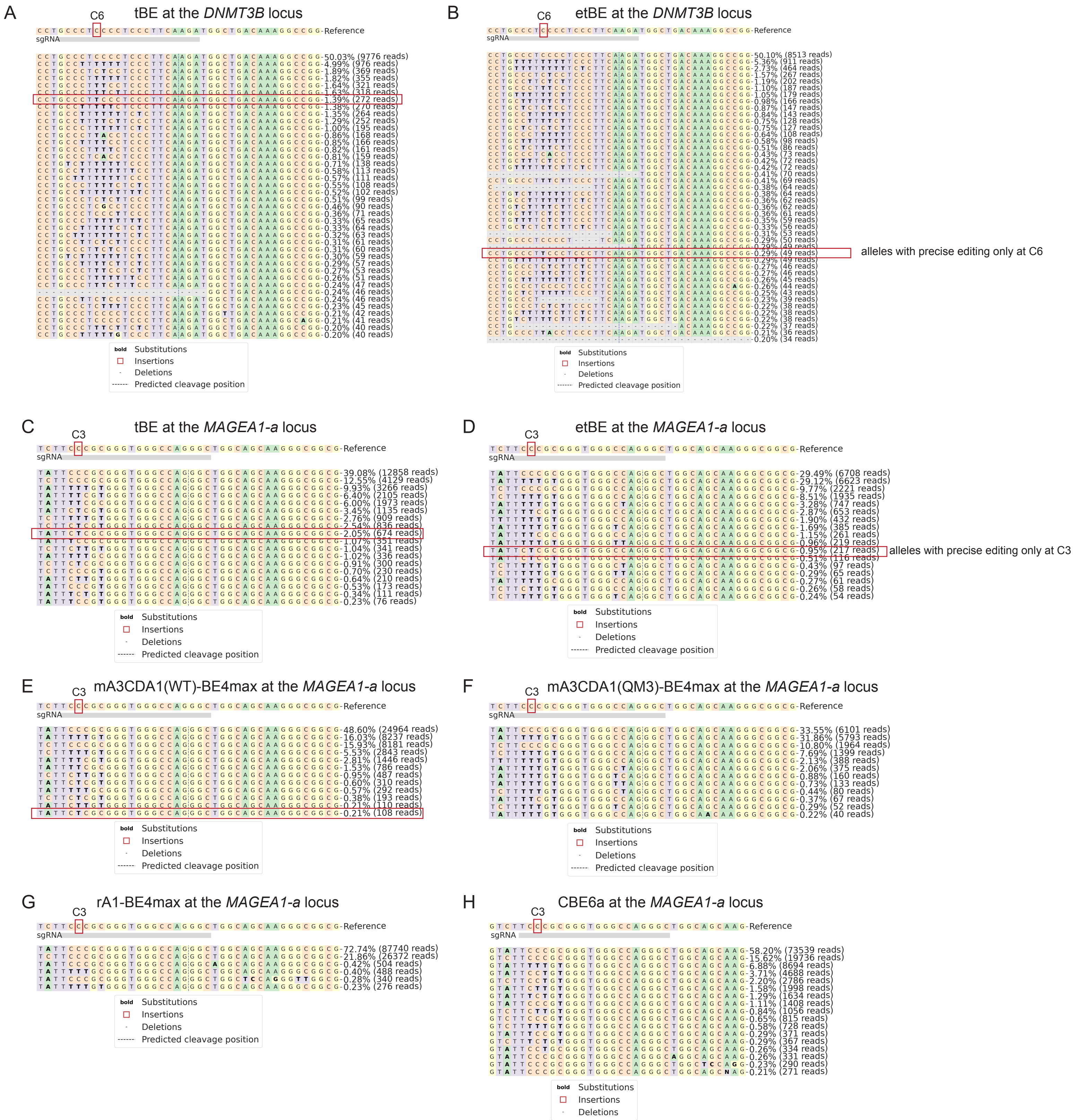

Figure S24. Allele frequency analysis of tBE and etBE at the *DNMT3B* and *MAGEA1-a* endogenous loci. (A and B) Allele frequency tables of tBE (A) and etBE (B) at the *DNMT3B* locus. (C–H) Allele frequency tables of tBE (C), etBE (D), mA3CDA1(WT)-BE4max (E), mA3CDA1(QM3)-BE4max (F), rA1-BE4max (G), and CBE6a (H) at the *MAGEA1-a* locus. Alleles with precise editing only at the intended cytosine position are highlighted in red boxes. Data were analyzed using CRISPResso2 with default settings. For mA3CDA1(QM3), rA1-BE4max, and CBE6a, the allele frequency with only C3 modified is low (less than 0.23%) and is therefore not displayed in the tables.

Supplementary Table 1. Constructs used in this study, including hsgRNA and sgRNA sequences, corresponding PAM sequences, and oligonucleotides for plasmid construction.

Supplementary Table 2. Endogenous genomic loci tested in this study, with corresponding primers and amplicons.

Supplementary Table 3. Off-target loci evaluated in this study, including PAM sequences, mismatches, genomic coordinates, primers, and amplicons.
